# Spatial coupling of enlarged perivascular spaces and white matter lesions across the Alzheimer's disease continuum

**DOI:** 10.3389/fnins.2026.1772024

**Published:** 2026-04-01

**Authors:** Serena Tang, Pamela Thropp, Isabella Hausle, Kyan Younes, Duygu Tosun

**Affiliations:** 1 Berkeley-UCSF Graduate Program in Bioengineering, University of California, Berkeley, Berkeley, CA, United States; 2 Department of Radiology and Biomedical Imaging, University of California, San Francisco, San Francisco, CA, United States; 3 Department of Radiology, Northern California Institute for Research and Education, San Francisco, CA, United States; 4 Department of Neurology, Stanford University School of Medicine, Stanford, CA, United States

**Keywords:** Alzheimer's disease, deep learning, perivascular space, white matter hyperintensities, glymphatic clearance

## Abstract

**Introduction:**

Emerging evidence suggests that impaired waste-clearance systems contribute to Alzheimer's disease pathogenesis, yet the etiology of clearance dysfunction markers, such as enlarged perivascular spaces, remains unclear. Because enlarged perivascular spaces and white matter lesions are both consequences of microvascular injury involving neuroinflammation and impaired cerebrovascular function, we hypothesize that these markers may be spatially coupled through local interstitial fluid stagnation, where impaired perivascular clearance associates with white matter injury.

**Methods:**

We assessed global perivascular space differences and correlations across diagnostic and biomarker-informed groups in the Alzheimer's Disease Neuroimaging Initiative dataset within whole brain, white matter, and basal ganglia regions, as well as within and outside of white matter lesions. To assess the spatial relationships between enlarged perivascular spaces and white matter lesions, we examined perivascular space distribution at distances away from white matter lesions.

**Results:**

Group-wise analyses revealed greater perivascular space counts and volumes within the white matter lesions and the basal ganglia in the mild cognitively impaired versus cognitively unimpaired group. Perivascular space counts and volumes and white matter lesion volumes correlated significantly within basal ganglia and white matter lesion regions across the cohort, with no differences in this relationship across diagnostic groups. Spatial analyses demonstrated greater perivascular space density within 5–15 mm of white matter lesions in mild cognitively impaired-amyloid positive and all amyloid positive groups compared to cognitively unimpaired-amyloid negative groups and all amyloid negative groups respectively, but reduced density ≥30 mm from white matter lesions in the Alzheimer's diagnosed-amyloid positive versus cognitively unimpaired-amyloid negative groups. White matter lesion volume consistently predicted perivascular spaces counts across all distance bins, with associations weakening as distance from white matter lesions increased. These results were all age and sex adjusted, indicating that the observed changes may reflect pathological processes beyond normal aging.

**Discussion:**

These findings demonstrate spatial coupling between enlarged perivascular spaces and white matter lesions across the Alzheimer's disease continuum, with coupling changes emerging early in disease stages, supporting the hypothesis that local perivascular clearance dysfunction and white matter injury represent interacting pathological processes that may serve as early biomarkers of Alzheimer's disease.

## 1 Introduction

Alzheimer's disease (AD) is a multifactorial disorder with etiology that frequently overlaps with other neurodegenerative disorders, such as cerebrovascular disease (CVD; Wardlaw et al., 2020; Scheffer et al., 2021; Attems and Jellinger, 2014), complicating diagnosis and treatment. Emerging evidence suggests that vascular dysfunction may precede AD pathology (Scheffer et al., 2021); however, the mechanisms by which vascular deficits contribute to neurodegeneration remain incompletely characterized. One proposed mechanism involves impaired waste clearance. Studies suggest that the glymphatic system, responsible for clearing cellular waste from the interstitium, is driven by arterial pulsations (Agarwal et al., 2024; Kedarasetti et al., 2022; Mestre et al., 2018). Consequently, deficits in cerebrovascular function may hinder clearance efficiency, promoting the accumulation of neurotoxic proteins such as beta-amyloid (Aß), a hallmark of AD, and contributing to AD pathophysiology (Bacyinski et al., 2017; Iliff et al., 2012; Hablitz and Nedergaard, 2021). To better understand this cascade, it is necessary to examine how vascular biomarkers change over disease stages.

Enlarged perivascular spaces (EPVS) and white matter lesions (WML) are characteristic features of vascular disease that exhibit distinct patterns within AD (Wardlaw et al., 2020; Solé-Guardia et al., 2025; Shirzadi et al., 2023). EPVS are fluid-filled compartments surrounding the brain's microvasculature that serve as the structural conduit for glymphatic flow (Wardlaw et al., 2020; Agarwal et al., 2024; Iliff et al., 2012; Hablitz and Nedergaard, 2021). Recent studies suggest that in the context of reduced CSF flow or cerebrovascular rigidification, PVS enlargement may represent either a compensatory response or mechanical reaction to upstream blockages to maintain flow capacity rather than a purely neurodegenerative feature (Younes et al., 2023; Menze et al., 2024; Brown et al., 2018; Barnes et al., 2022). For instance, histopathological studies have demonstrated that vascular amyloid deposits—a hallmark of cerebral amyloid angiopathy (CAA)—are spatially congruent with EPVS (Perosa et al., 2022), suggesting that local fluid stagnation or drainage obstruction contributes to perivascular dilation. Furthermore, physiological impairments such as reduced cerebrovascular reactivity (Libecap et al., 2023) and increased arterial stiffness (Riba-Llena et al., 2018) have been directly associated with greater EPVS burden in older adults. Thus, vascular impairment likely manifests as increased EPVS burden alongside other markers of injury, such as WML (Menze et al., 2024; Brown et al., 2018; Ballerini et al., 2020; Wardlaw et al., 2015).

In the context of AD, the relationship with EPVS remains complex. Some studies report increased EPVS count and volume with AD progression, particularly in the white matter (WM-EPVS), where they are strongly associated with cognitive impairment (Wang et al., 2022; Hong et al., 2024; Kim et al., 2021; Sepehrband et al., 2021). EPVS burden has also been correlated with amyloid pathology within individuals diagnosed with AD (Perosa et al., 2022; Hong et al., 2024; Kim et al., 2021), supporting a potential link to impaired clearance mechanisms. Conversely, other studies have found no association between EPVS and AD diagnosis or amyloid burden (Younes et al., 2023; Jeong et al., 2022; Banerjee et al., 2017). These discrepancies may stem from inconsistent quantification methods or cohort heterogeneity, which obscure the specific contribution of cerebrovascular pathology to AD (Wardlaw et al., 2020; Francis et al., 2019; Liu et al., 2025).

Despite the mixed findings in AD, EPVS consistently correlates with biomarkers of CVD, particularly WML burden (Menze et al., 2024; Kim et al., 2021; Ong et al., 2025; Liu et al., 2025; Gertje et al., 2020; Farrall and Wardlaw, 2009; Wang et al., 2021). While the etiology of WML is multifactorial and manifests in various neurodegenerative diseases beyond CVD and AD, evidence suggests a shared pathogenesis with EPVS rooted in endothelial dysfunction and blood-brain barrier breakdown (Solé-Guardia et al., 2025; Wardlaw et al., 2015; Farrall and Wardlaw, 2009). Impaired perivascular clearance may lead to local interstitial fluid stagnation (Perosa et al., 2022; Ong et al., 2025; Wang et al., 2021), resulting in perivascular white matter injury that manifests as WML (Libecap et al., 2023). Alternatively, perivascular enlargement may occur secondarily around established ischemic lesions (Younes et al., 2023; Barnes et al., 2022; Wardlaw et al., 2015). Whether clearance failure precedes WML development or vice versa remains unknown; however, both likely participate in a vicious cycle driven by underlying disease pathology (Wardlaw et al., 2020; Gouveia-Freitas and Bastos-Leite, 2021). Characterizing how the EPVS-WML relationship changes across the AD continuum may reveal distinct patterns that help differentiate disease stages and identify stage-specific pathological processes.

A few studies have looked at EPVS–WML relationships across the AD continuum (Younes et al., 2023; Menze et al., 2024; Kim et al., 2021; Ong et al., 2025; Liu et al., 2025; Wu et al., 2025), yet most of these analyses focus on global volumetric associations rather than local topology. Thus, of particular interest is characterizing their spatial associations—if EPVS and WML share mechanistic drivers, they should exhibit spatial clustering; if driven by distinct processes, spatial dissociation may occur. While studies in healthy cohorts indicate that WML predominantly develop in proximity to EPVS (Barnes et al., 2022; Huang et al., 2021; Huo et al., 2023), their spatial relationship within AD remains uncharacterized. We hypothesize that the spatial coupling between EPVS and WML varies across disease stages due to the evolving dominance of distinct pathological mechanisms (Scheffer et al., 2021; Aisen et al., 2024). In early disease stages (e.g., mild cognitive impairment; MCI), we expect a strong spatial correlation, as perivascular clearance failure—potentially driven by arterial stiffening or initial amyloid aggregation—may lead to local interstitial fluid stagnation that promotes white matter injury in the immediate vicinity. Conversely, in advanced AD, the relationship may be altered by severe atrophy or dense amyloid deposition (as seen in CAA), which could lead to a redistribution of EPVS, decoupling EPVS from WML or reducing their proximity. Investigating this stage-dependent topology is critical to elucidating whether these markers reflect a continuous, shared pathogenesis or interacting processes that decouple as neurodegeneration progresses.

In this study, we leveraged deep learning-based segmentation maps to analyze the spatial coupling between EPVS and WML. Using the Alzheimer's Disease Neuroimaging Initiative 3 (ADNI-3) dataset, we evaluated these associations across clinical and biomarker-informed disease stages. To investigate our hypothesis, we aimed to: (1) assess global burden and regional associations of EPVS across clinical diagnoses and amyloid status; (2) determine the global relationship between EPVS and WML burden; (3) quantify the spatial topology of EPVS around WML to determine if they exhibit local spatial coupling that varies by disease stages; and (4) examine whether total WML burden is associated with the spatial distribution of EPVS.

## 2 Methods

### 2.1 Participants

Data used in the preparation of this article were obtained from the Alzheimer's Disease Neuroimaging Initiative (ADNI) database (adni.loni.usc.edu). The primary goal of the ADNI has been to test whether serial magnetic resonance imaging (MRI), positron emission tomography (PET), other biological markers, and clinical and neuropsychological assessment can be combined to measure the progression of mild cognitive impairment (MCI) and early Alzheimer's disease (AD). Each study site obtained respective IRB approval for the ADNI protocols.

For this study, participants from ADNI-3 (*n* = 1,080) who were diagnosed as cognitively unimpaired (CU; *n* = 593), mild cognitively impaired (MCI; *n* = 369), or dementia due to AD (*n* = 118) were included. Data containing their demographic, imaging, genetic, and diagnostic information were obtained from LONI (ida.loni.usc.edu). Inclusion, exclusion, and diagnostic criteria can be found at (Jack et al. 2024b). Notably, individuals were excluded if they had vascular dementia (Hachinski score > 4), thus providing a cohort to study the natural history of AD. For this analysis, individuals were also excluded if they did not have a matching T1 and FLAIR image.

### 2.2 Structural imaging

The T1-weighted (T1w) and FLAIR images used in this study were acquired using the multisite, multi-scanner ADNI-3 protocol (Jack et al., 2024b).

### 2.3 MRI preprocessing

T1w image preprocessing and parcellation were completed using Freesurfer 6.0.0 (Fischl, 2012). First, images were denoised using non-local means denoising using Advanced Normalization Tools (ANTs) (Avants et al., 2011), before input into the *recon-all* module of Freesurfer. This includes motion correction, nonparametric nonuniform normalization (N4) bias field correction, and intensity normalization (where voxels are scaled so the mean intensity of white matter is 110 (Fischl, 2012). Intracranial volumes and regional parcellations were derived from the Desikan-Killany atlas. FLAIR images were N4 bias-field corrected using ANTs before being coregistered using affine transformations to their corresponding T1w images using Freesurfer's *mri_coreg* and *mri_convert* with 6 degrees of freedom.

#### 2.3.1 EPVS segmentation model

To segment EPVS, we employed the nnU-Net model, a self-configuring, deep-learning framework based on U-Net architecture, which has demonstrated state-of-the-art performance across various medical imaging benchmarks (Isensee et al., 2021). Specifically, we utilized the default 3D full-resolution configuration using nnU-Netv2. To generate the ground-truth dataset for training, 60 participants were selected from ADNI-3 (20 CU, 20 MCI, 20 AD) and were manually segmented by a trained rater (S.T.) under the guidance of an experienced behavioral neurologist (K.Y.) using ITK-SNAP, strictly adhering to STRIVE criteria (Potter et al., 2015a). To ensure WML were distinguished from EPVS, coregistered T1w and FLAIR images were viewed in conjunction during manual delineation. The deep learning model was trained using both T1w and FLAIR images as dual-channel inputs, allowing the model to learn to exclude WML based on their distinct intensity characteristics. The dataset was partitioned into a training and validation data set (*n* = 50) and a held-out test data set (*n* = 10). Training was performed over 1,000 epochs using the standard five-fold cross-validation scheme to maximize model robustness (Isensee et al., 2021). We utilized the default nnU-Net data augmentation pipeline (including rotation, scaling, and elastic deformation). Preliminary experiments using intensity-normalized FLAIR inputs, Contrast Limited Adaptive Histogram Equalization (CLAHE), and the ResNet-based nnU-Net variant yielded no significant performance improvements; therefore, the standard nnU-Net architecture was retained for its computational efficiency. No further preprocessing was done to the input images since T1w images from ADNI-3 are 1 × 1 × 1 mm isotropic resolution, intensity normalized, and FLAIR images were registered to T1w images as part of the preprocessing step. Training was conducted on a high-performance computing cluster using NVIDIA A40 GPUs with at least 10GiB of VRAM allocated per fold (approximately 1 min per image pair).

#### 2.3.2 Regional masking and volumetrics

We quantified the segmentation results by extracting EPVS count and volume using a connected components analysis using the Quantitative Imaging Toolbox (QIT; https://cabeen.io/qitwiki; Cabeen et al., 2018). WML segmentation masks were generated using the Lesion Prediction Algorithm (LST toolbox version 3.0.0 for SPM; Schmidt, 2017, Chapter 6.1; Schmidt et al., 2012). To facilitate regional analysis, we generated five binary masks: whole-brain (WB), white matter (WM), basal ganglia (BG), WML, and non-WML. The WM mask was generated from the *wmparc.mgz* output of *recon-all*, while the BG mask was created from the *aparc*+*aseg.mgz* output and included the caudate nucleus, putamen, globus pallidus, nucleus accumbens, and thalamus. Regional EPVS masks were created by calculating the intersection of the global EPVS segmentation with the respective anatomical masks using the QIT *MaskSet* function or Python Nibabel library. Finally, all EPVS and WML volumes were normalized by intracranial volume (ICV) to account for inter-subject head size variability.

#### 2.3.3 Model evaluation

Model accuracy was assessed using Dice similarity coefficients, including both voxel-wise and lesion-wise Dice, as well as precision and recall. Overall scores were calculated based on whole-brain analyses, representing the total overlap between predicted and ground-truth segmentation per image. Voxel-wise Dice calculations were performed by averaging the Dice score per lesion (i.e., true positives are calculated by summing the number of voxels that overlap per lesion, averaging the sum over each image, and then averaging over individuals). Lesion-wise Dice scores were calculated by considering a true positive as a lesion where the model segmented at least one voxel correctly, summing the total lesions captured per image, and averaging across individuals. Additionally, we calculate the Spearman correlation between predicted and ground-truth EPVS counts and volumes using whole-brain segmentation. Given that automated segmentation models often exhibit performance degradation in neurodegenerative cohorts due to atrophy and image heterogeneity, accuracy metrics were also assessed across diagnostic groups (CU, MCI, AD) to evaluate potential bias. Regional performance was validated by assessing accuracy within WM, BG, WML, and non-WML regions.

#### 2.3.4 Spatial quantification of EPVS and WML

**Figure 1:**
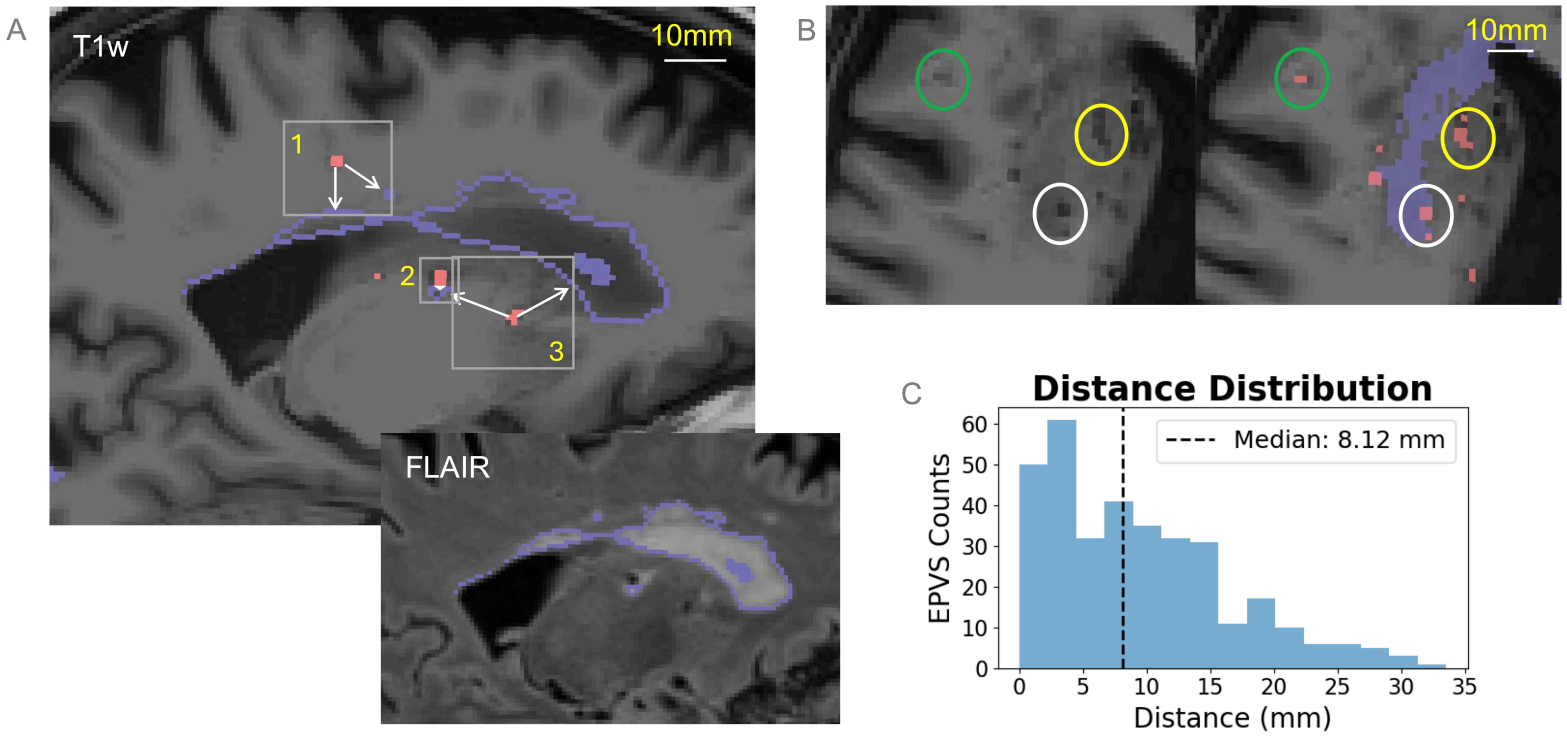
Demonstration of distance calculations. **(A)** A search radius (grey box) is assigned to each EPVS (red) until the neighborhood includes WML voxels (purple). Distance from the EPVS is then calculated to each WML voxel, and the minimum distance is selected for that EPVS (e.g., for EPVS 1, the minimum is taken across the two possible distances, indicated by two white arrows). This process is repeated for each EPVS. Example of the co-registered FLAIR image is shown on the bottom. **(B)** Examples of EPVS that are within, close to and far from WML. The white circle denotes an EPVS within WML; the yellow circle denotes an EPVS close to WML, and green denotes an EPVS far from WML. **(C)** Example distribution of distances from the individual depicted. In this individual, more EPVS are close to WML, and the count of EPVS decreases as the distance from WML increases.

To investigate the spatial relationship between EPVS and WML, we used the T1w images to calculate the minimum Euclidean distance in millimeter (mm) from each EPVS centroid to the nearest WML boundary. First, WML boundary masks were generated by eroding the original WML mask by one voxel using a cross-element kernel (QIT *MaskErode*) and subtracting the eroded mask from the original WML mask. EPVS segmentation masks were processed using the *cc3d* Python package to identify connected components, from which volumes and centroid coordinates were derived. Centroids of EPVS were used as a surrogate measure of their general location. We used a custom iterative search algorithm to determine the minimum distance to a WML lesion. For each EPVS centroid, a cubic search window of increasing radius (initial *r* = 1 voxel) was expanded until it intersected with a voxel in the WML boundary mask. The Euclidean distance between the EPVS centroid and the identified WML boundary voxel was then calculated. This process yielded a quantitative distance metric for every individual EPVS within the non-WML mask. This distance calculation was performed for all segmented EPVS (including those in the BG and WM) to characterize the global topology of perivascular burden relative to lesion boundaries. Consequently, the distance metrics reflect the proximity of any EPVS to any nearest WML, regardless of the tissue class (gray or white matter) in which the EPVS resides. EPVS within WML were excluded in this calculation and considered to be at distance 0 mm. This process is illustrated in Figure 1.

### 2.4 Amyloid PET imaging and analysis

Amyloid PET imaging data were acquired and processed in accordance with the UC Berkeley PET processing protocols (Jagust et al., 2024). PET scans were matched to the closest available MRI within ±9 months. Data included [18F] florbetaben (FBB) and [18F] florbetapir (FBP). Images were smoothed to a resolution of 6 mm, rigidly registered to the corresponding structural MRI, and parcellated using Freesurfer version 7.1 (Jagust et al., 2024). The cortical standard uptake value ratios (SUVRs) were extracted from a composite region of interest including frontal, anterior/posterior cingulate, lateral parietal, and lateral temporal regions. SUVRs were normalized to the whole cerebellum (Jagust et al., 2024). Amyloid positivity was determined based on established SUVR thresholds (1.11 for FBP PET and 1.08 for FBB PET; Josephs et al., 2022).

### 2.5 Genotyping

Apolipoprotein (*APOE*) genotyping was conducted on DNA extracted from blood samples following the standard ADNI-3 protocol (Saykin et al., 2015). Participants were classified based on the presence of the ε4 allele. *APOE*-ε4 status was defined as positive for individuals carrying at least one ε4 allele (heterozygous or homozygous) and negative for non-carriers.

### 2.6 Statistical analysis

#### 2.6.1 Data transformations

We observed that EPVS counts and volumes followed non-normal, right-skewed distributions. To address this, we utilized Generalized Linear Models (GLM) tailored to each data type. For EPVS counts, we utilized Negative Binomial regressions. For the WML region specifically (where ~60% of counts were zero), we employed Zero-Inflated Negative Binomial models. In contrast, for EPVS and WML volumes, we applied a natural log transformation to correct for skewness and heteroscedasticity, followed by GLM with a Gaussian link function. Post-transformation normality was verified using the Breusch-Pagan test and visual inspection of residual plots.

#### 2.6.2 Group-wise global and regional comparisons

Demographic differences across clinical diagnostic groups (CU, MCI, dementia due to AD), amyloid status (Aβ±), and biomarker-informed stages (CUAβ-, CUAβ+, MCIAβ+, ADAβ+) were assessed using Chi-squared tests for categorical variables and Student's *t*-tests for continuous variables. For assessing differences in EPVS counts and volumes across groups, we constructed models with EPVS counts or volumes as the outcome variable and group status as the independent variable.

#### 2.6.3 Global relationship between EPVS and WML

To determine if the relationship between EPVS and WML changes over the disease course, we first assessed the association between EPVS (count and volume) and WML volume within each ROI using the regression models described above. Subsequently, we tested for interaction effects between WML volume and disease status (i.e., clinical diagnosis, amyloid status, or biomarker-informed stage) to determine if the slope of the EPVS-WML relationship differed by group. EPVS counts and EPVS volumes were set as the outcome variables, and WML volume was set as the independent variable. Pairwise contrasts were calculated between sequential disease stages (e.g., CUAβ+ vs. MCIAβ+ and MCIAβ+ vs. ADAβ+).

#### 2.6.4 Spatial distribution of EPVS from WML

We investigated the spatial topology of EPVS relative to WML using two approaches. First, we categorized EPVS based on their proximity to the nearest WML: “within WML” (0 mm), “near WML” ( ≤ 5 mm), and “far from WML” (>5 mm). The 5 mm threshold was chosen to approximate the WML penumbra, a region vulnerable to future lesion growth (Voorter et al., 2025; Maillard et al., 2011; Wang et al., 2023). Second, for a more granular spatial analysis, we binned distances in 5 mm increments (i.e., 0–5 mm, 5–10 mm, etc.) from 0 mm (within WML) to 30 mm, with a final category for distances >30 mm. Negative Binomial regression was used to model EPVS counts within each distance bin against group status.

Additionally, to assess whether the total WML burden influences the spatial distribution of EPVS, we modeled EPVS counts at each distance bin as a function of total WML volume, testing for interactions with groups. Sensitivity analyses included WML log-volume as a covariate to account for potential confounding by lesion extent.

#### 2.6.5 Covariates, effect size calculations, and multiple comparison correction

Age and sex were selected as fixed-effect covariates a priori based on their potential as confounders of both WML and EPVS burden. Genetic risk factors (*APOE*-ε4) and education, although significant between diagnostic groups (Table 1), were not included in the primary models as they are upstream determinants of small vessel disease and could constitute over-adjustment; however, sensitivity analysis with *APOE*-ε4 and education are included in Supplementary Tables S2–S7. To control for Type I error, *p*-values were adjusted for multiple comparisons using the False Discovery Rate (FDR) method (Benjamini-Hochberg) within each family of hypothesis tests. Statistical significance was defined at α = 0.05. In this study, for the Negative Binomial or Zero-Inflated Negative Binomial models, we report the ratio of expected counts (β_exp_), calculated by exponentiating the beta coefficients. These can be interpreted as: for a one-unit change in the dependent variable (or for the group-wise comparisons, a change in the group), the expected count multiplies by a factor given by β_exp_. For the GLM, we report standardized beta coefficients. Confidence intervals (CI) are calculated based on these values. Effect sizes for beta coefficients were considered small at < 0.1, medium at < 0.3, and large at >0.5 for GLM. For β_exp_, a decrease in ratio is β_exp_ < 1, no change in ratio is β_exp_ = 0, and a ratio increase is β_exp_ > 1.

**Table 1:** Demographics and clinical characteristics.

Characteristics	Total	CU	MCI	AD
***N***	1,080	593	369	118
**Age (years; mean** **±SD)**	74.05 ± 8.26	73.02 ± 8.13	74.73 ± 8.11	77.12 ± 8.39
**Sex (*****n*** **females, %)**	566, 52.41%	352, 59.36%	163, 44.17%	51, 43.22%
**Education** **>16 years (*****n*****, %)**	763, 70.65%	447, 75.38%	252, 68.29%	64, 54.24%
**Race (NHW/NHB/HW/A/L/O**, ***n*****, %)**	839, 77.69%	432, 72.85%	308, 83.47%	99, 83.90%
	119, 11.02%	78, 13.15%	31, 8.40%	10, 8.47%
	59, 5.46%	39, 6.58%	17, 4.61%	3, 2.54%
	31, 2.87%	24, 4.05%	4, 1.08%	3, 2.54%
	14, 1.30%	11, 1.85%	3, 0.81%	0, 0.00%
	18, 1.67%	9, 1.52%	6, 1.63%	3, 2.54%
**Amyloid positivity (*****n*** **Aβ+, %)**	361, 38.40%	140, 26.52%	139, 44.55%	82, 82.00%
***APOE*****-ε4 status (*****n*** **E4+, %)**	364, 37.64%	174, 32.40%	127, 37.69%	63, 77.78%
**WML volume (% of ICV** **±SD)**	0.60 ± 0.69%	0.51 ± 0.57%	0.69 ± 0.80%	0.83 ± 0.80%

**Aβ+** **subgroups [*****N*** **(# missing)]**	361 (140)	140 (65)	139 (57)	82 (18)
Age (mean ± SD)	75.92 ± 8.05	75.06 ± 7.87	75.73 ± 7.74	77.70 ± 8.85
Sex (*n* females, %)	184, 50.97%	84, 60.00%	65, 46.76%	35, 42.68%
Education >16 years (*n*, %)	238, 65.93%	103, 73.57%	93, 66.91%	42, 51.22%
Race (NHW/NHB/HW/A/L/O, *n*, %)	297, 82.27%	109, 77.86%	118, 84.89%	70, 85.37%
	33, 9.14%	16, 11.43%	12, 8.63%	5, 6.10%
	15, 4.16%	8, 5.71%	4, 2.88%	3, 3.66%
	6, 1.66%	3, 2.14%	0, 0.00%	3, 3.66%
	1, 0.28%	0, 0.00%	1, 0.72%	0, 0.00%
	9, 2.49%	4, 2.86%	4, 2.88%	1, 1.22%
*APOE*-ε4 status (*n* E4+, %)	264, 73.13%	74, 54.01%	127, 63.50%	63, 67.07%
WML volume (% of ICV ± SD)	0.74 ± 0.83%	0.64 ± 0.66%	0.76 ± 0.95%	0.87 ± 0.88

CU, Cognitively Unimpaired; MCI, Mild Cognitive Impairment; AD, Alzheimer's Disease; Aβ, Amyloid-beta; NHW, Non-Hispanic White; NHB, Non-Hispanic Black; HW, Hispanic White; A, Asian; L, Latino; O, Other; SD, Standard Deviation. 140 individuals were missing amyloid status data; thus, the numbers for the Aβ+ subgroups correspond to a subset of the entire dataset (i.e., 940 individuals).

## 3 Results

### 3.1 Segmentation model evaluation

**Figure 2:**
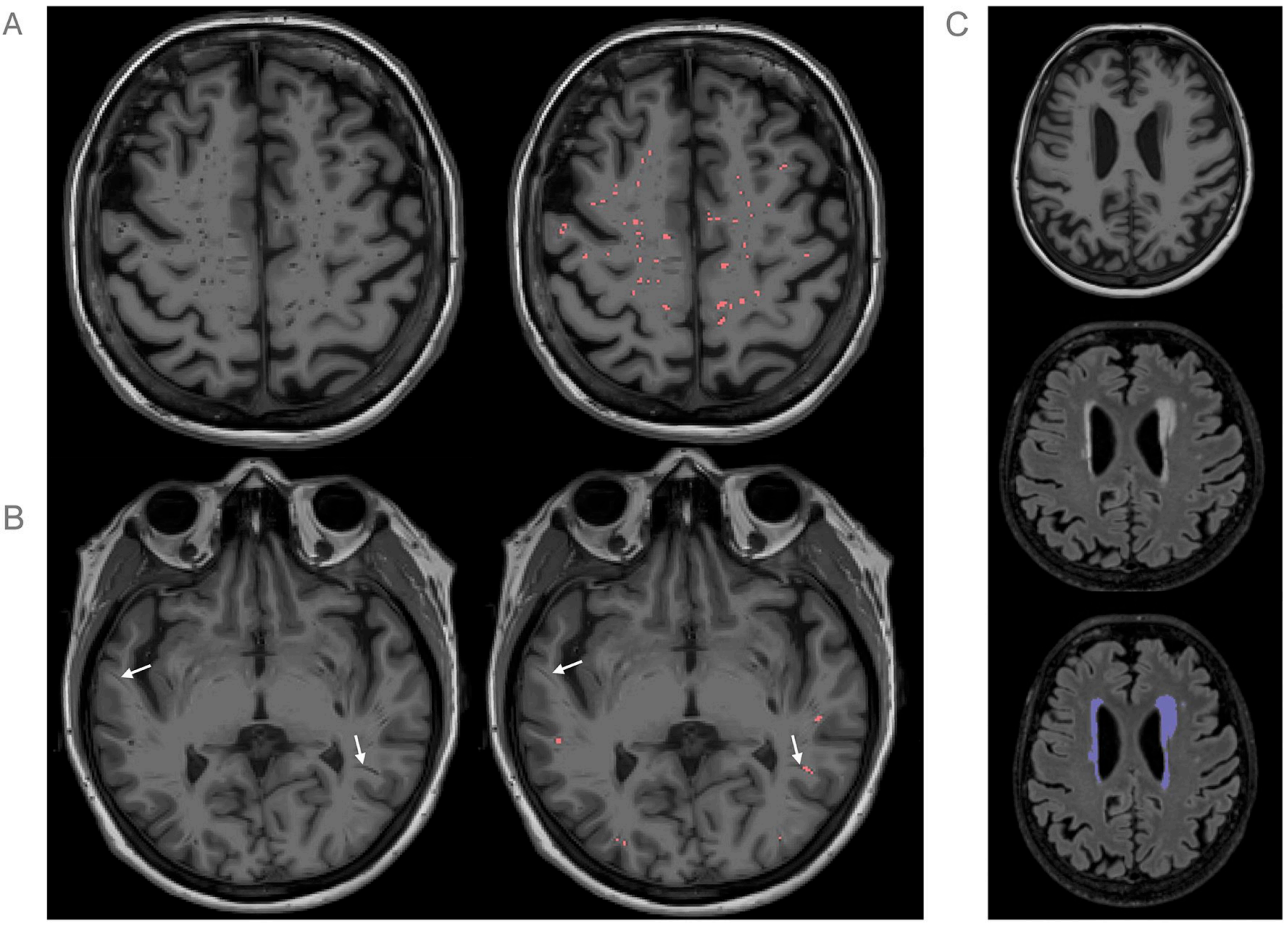
**(A)** Example EPVS segmentation by the model; segmentation is shown in red. Top row shows the image with (right) and without (left) the segmentation mask. The majority of small and large EPVS are segmented successfully in this case. **(B)** Examples of EPVS segmentation errors within the same individual. White arrows point to areas where the model either missed an EPVS (left arrow) or only segmented part of an EPVS (right arrow). **(C)** Example of WML segmentation. Top to bottom: T1, FLAIR, and FLAIR image with WML segmentation (denoted in purple).

The deep learning model achieved robust performance, with a precision of 0.66 ± 0.17, recall of 0.62 ± 0.20, and an overall Dice of 0.61 ± 0.12 (voxel-wise Dice = 0.74 ± 0.07 and lesion-wise Dice of 0.63 ± 0.11). The automated segmentations showed high correlation with ground-truth manual segmentations for both EPVS counts (ρ = 0.85, *p* < 0.001) and volumes (ρ = 0.88, *p* < 0.001). Performance remained consistent across regions and disease stages (Table 2), supporting the validity of the segmentation masks for group-wise comparisons. An example segmentation visualization is shown in Figure 2.

**Table 2:** Accuracy metrics of the EPVS segmentation results from the nn-Unet model.

Dataset	Segmentation metrics					EPVS count correlation		EPVS volume correlation	
	**Precision**	**Recall**	**Overall dice**	**Voxel-wise dice**	**Lesion-wise dice**	**Spearman** ρ **(*****p*****)**	**Lin's CCC**	**Spearman** ρ **(*****p*****)**	**Lin's CCC**
Overall									
Validation set (*n* = 50)	0.66 ± 0.17	0.61 ± 0.16	0.60 ± 0.10	–	–	–	–	–	–
Test set (*n* = 10)	0.66 ± 0.17	0.62 ± 0.20	0.61 ± 0.12	0.74 ± 0.07	0.63 ± 0.11	0.85, *p* = 0.001	0.57	0.88, *p* < 0.001	0.75
Region									
WM (*n* = 10)	0.66 ± 0.22	0.66 ± 0.21	0.63 ± 0.13	0.82 ± 0.10	0.62 ± 0.14	0.97, *p* < 0.001	0.95	0.97, *p* < 0.001	0.88
BG (*n* = 10)	0.65 ± 0.18	0.53 ± 0.23	0.56 ± 0.15	0.72 ± 0.09	0.61 ± 0.12	0.93, *p* < 0.001	0.94	0.96, *p* < 0.001	0.91
WML (*n* = 3)	0.66 ± 0.31	0.51 ± 0.32	0.51 ± 0.25	0.69 ± 0.37	0.41 ± 0.09	0.82, *p* < 0.001	0.88	0.82, *p* < 0.001	0.77
Non-WML (*n* = 10)	0.66 ± 0.16	0.62 ± 0.20	0.61 ± 0.12	0.76 ± 0.06	0.47 ± 0.11	0.97, *p* < 0.001	0.95	0.97, *p* < 0.001	0.88
Diagnostic group									
CU (*n* = 20)	0.74 ± 0.20	0.67 ± 0.21	0.69 ± 0.19	0.81 ± 0.20	0.73 ± 0.15	0.96, *p* < 0.001	0.91	0.94, *p* < 0.001	0.94
MCI (*n* = 20)	0.71 ± 0.20	0.66 ± 0.20	0.67 ± 0.17	0.76 ± 0.19	0.72 ± 0.10	0.98, *p* < 0.001	0.97	0.95, *p* < 0.001	0.81
AD (*n* = 20)	0.74 ± 0.12	0.70 ± 0.11	0.72 ± 0.10	0.84 ± 0.07	0.69 ± 0.20	0.98, *p* < 0.001	0.92	0.98, *p* < 0.001	0.95

Validation accuracy metrics were obtained from the model's output of each of the five training folds' results and averaged across folds. Test set included 10 held-out images. Voxel-wise calculations were made by averaging the Dice score per lesion (i.e., asking “how much of each lesion was the model able to capture?”). Lesion-wise Dice scores were calculated by looking at where the model segmented at least one voxel correctly in each lesion, adding the total lesions captured, and averaging across individuals (i.e., asking “how many lesions did the model detect?”). Accuracy metrics for the diagnostic group were calculated using the model's inference results on the manually segmented dataset (due to the availability of a manually segmented dataset). Accuracy within regions was calculated within the test set only for the most accurate representation of model performance in each region. For the WML region, since there were few images with EPVS within WML, the accuracy metrics were averaged over images that had EPVS in WML regions.

EPVS, Enlarged Perivascular Spaces; CCC, Concordance Correlation Coefficient; WM, White Matter; BG, Basal Ganglia; WMH, White Matter Hyperintensities; CU, Cognitively Unimpaired; MCI, Mild Cognitive Impairment; AD, Alzheimer's Disease.

Values are presented as mean ± standard deviation unless otherwise specified. Spearman correlation coefficients (ρ) are shown with corresponding p-values. Lin's CCC measures agreement between automated and manual segmentation.

### 3.2 Demographic comparisons

In total, there were 1,080 individuals with baseline visit metrics included in this cross-sectional study. Demographic characteristics are reported in Table 1. The study cohort included participants who were cognitively unimpaired and PET amyloid negative (CUAβ–), or along the AD spectrum (CUAβ+, MCIAβ+, ADAβ+); 140 individuals excluded in the amyloid-related analyses because they did not have amyloid PET positivity information. Demographic comparisons across diagnostic groups and amyloid sub-groups showed significant differences in age, sex, race, education, and *APOE*-ε4 status (*p* < 0.001; Table 1, Supplementary Table S1A). Assessment of vascular risk profiles revealed that while the history of hypertension differed between diagnostic groups (*p* < 0.05 for CU vs. MCI and CU vs. AD), concurrent blood pressure measurements taken near the time of scanning did not differ significantly across groups (*p* > 0.05), likely reflecting effective medical management. Detailed comparisons of Modified Hachinski Scores and vascular risk factors are provided in Supplementary Table S1A.

**Figure 3:**
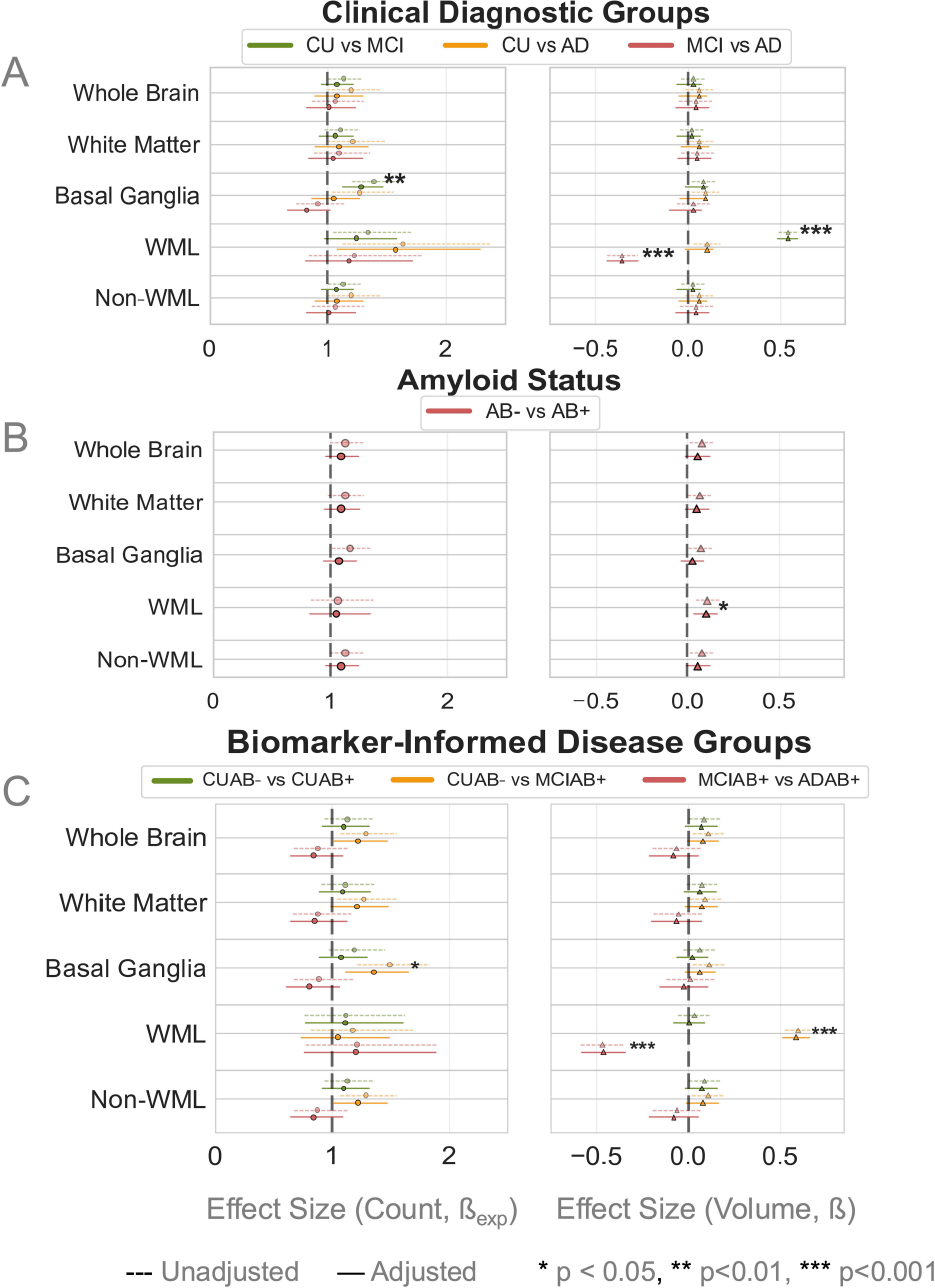
Effect sizes of EPVS counts and volume differences across clinical diagnostic groups **(A)**, amyloid status **(B)**, and biomarker-informed disease groups **(C)**. Each color denotes the comparison performed, as denoted in the figure legends. Significance stars denote the groups that were significantly different from each other in the age and sex adjusted analysis. Significant differences were found within WML and in BG regions: EPVS counts in BG regions were different in CU and MCI groups **(A)** as well as CUAβ– and MCIAβ+ **(C)**, while EPVS volumes were different in WML regions between CU and MCI, MCI and AD **(A)**, Aβ– and Aβ+ **(B)**, and CUAβ– and MCIAβ+, MCIAβ+ and ADAβ+ **(C)**, with less EPVS volume within WML in the AD groups.

**Figure 4:**
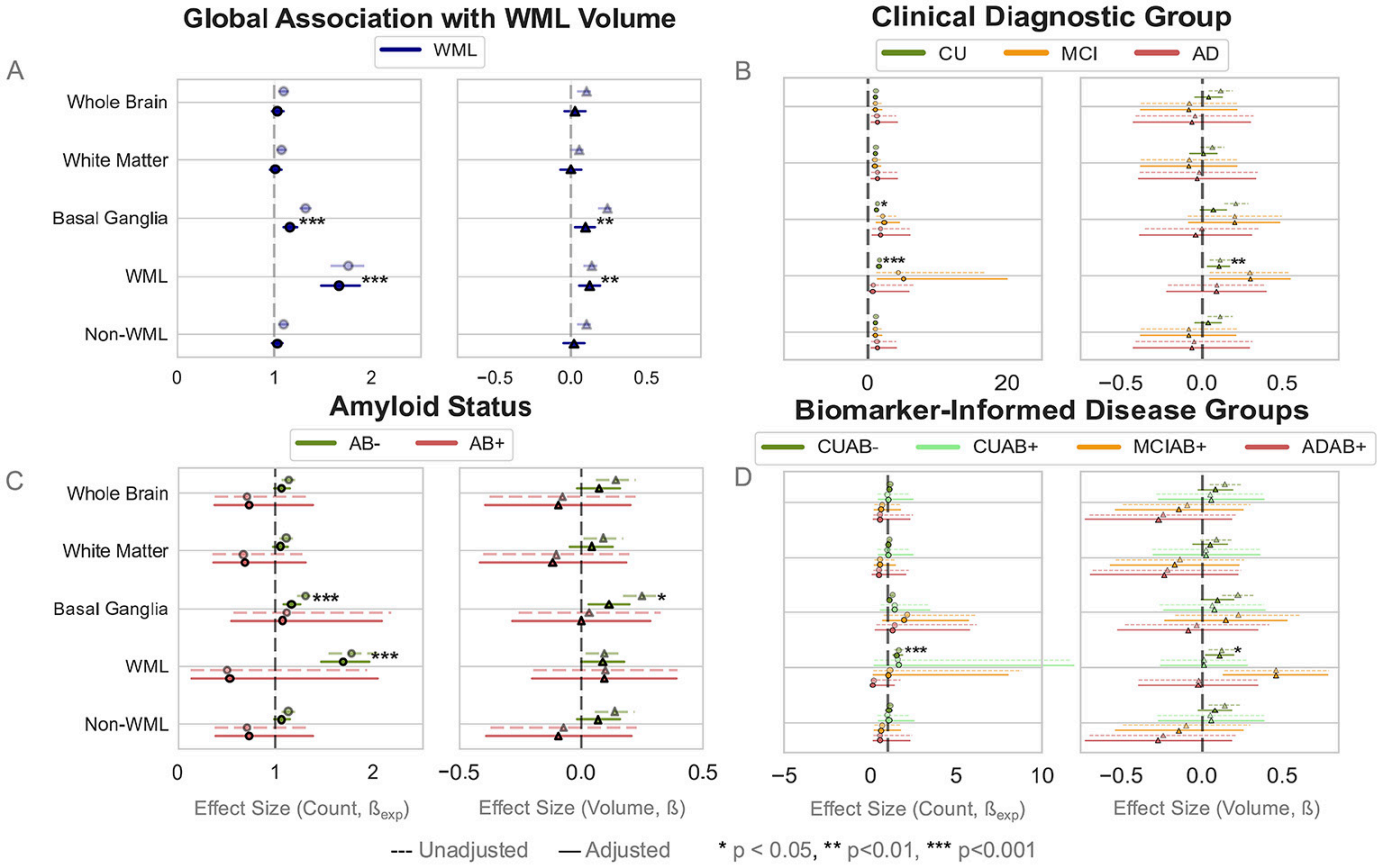
Forest plots show the effect sizes of the within-group correlation between EPVS count or volume and WML and the significance in that group (e.g., the correlation between EPVS and WML in CU individuals). **(A)** There was a significant relationship between the global association of WML volumes and EPVS count and volumes in the BG region and within WML. **(B–D)** Show forest plots for each comparison group, wherein there were mostly significant associations in CU, Aβ–, or CUAβ– groups within BG and WML. No significant interaction effects were observed between groups. Regression plots are provided in Supplementary Figure S2.

**Figure 5:**
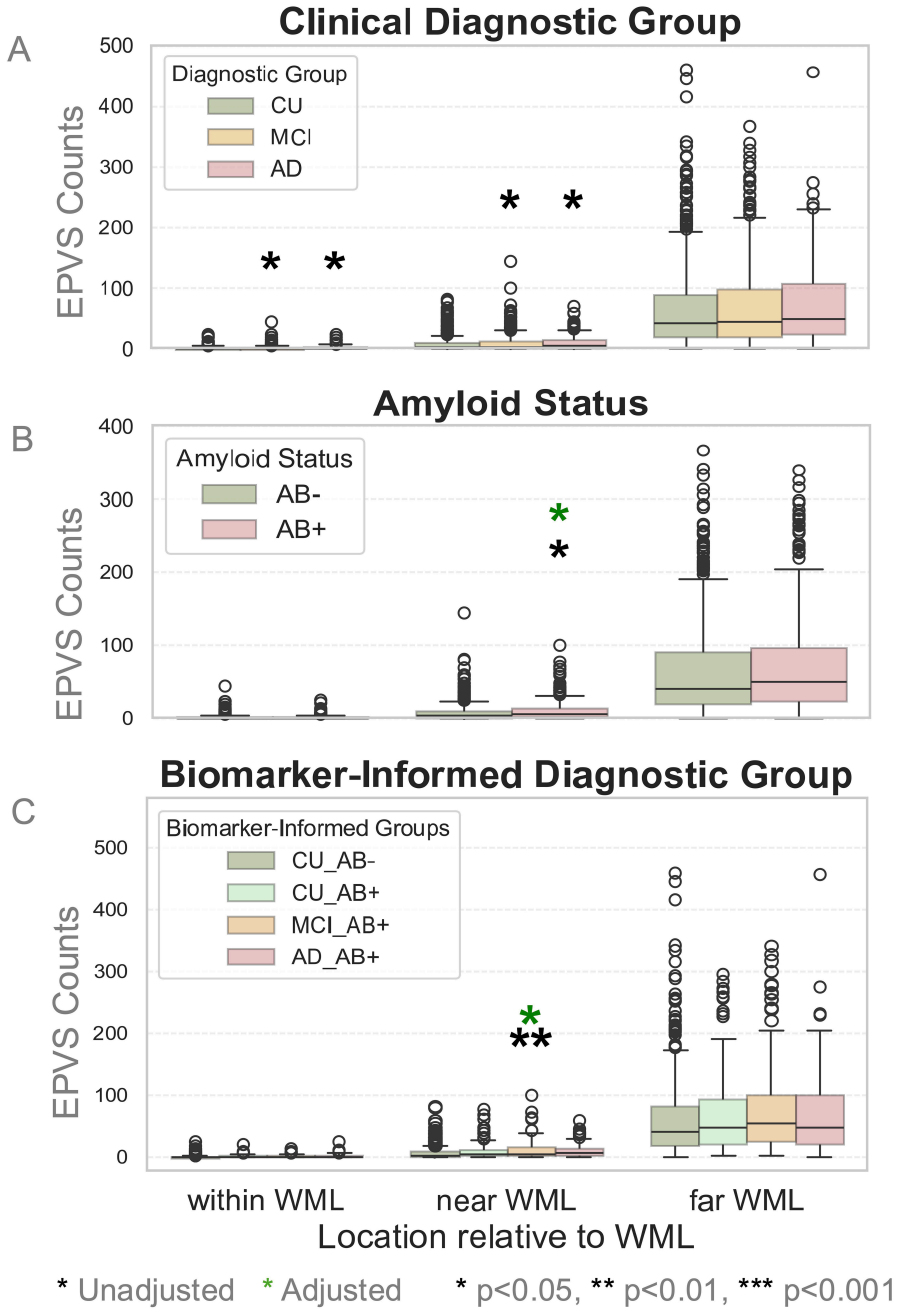
Box plots of EPVS within WML, near WML, and far from WML across **(A)** clinical diagnostic groups, **(B)** amyloid status, and **(C)** biomarker-informed diagnostic groups. Significance stars denote the adjusted significance levels in the difference between CUAβ– and the group that the star is placed above. In **(A)**, there were significant differences in EPVS counts within WML and near WML in both MCI and AD compared to CU, but these differences did not survive age and sex adjustment. In **(B)**, there were significant differences between Aβ+ and Aβ– in the regions near WML, even after age and sex adjustment. In **(C)**, a significant difference was found between MCIAβ+ and CUAβ– even after age and sex adjustment.

**Figure 6:**
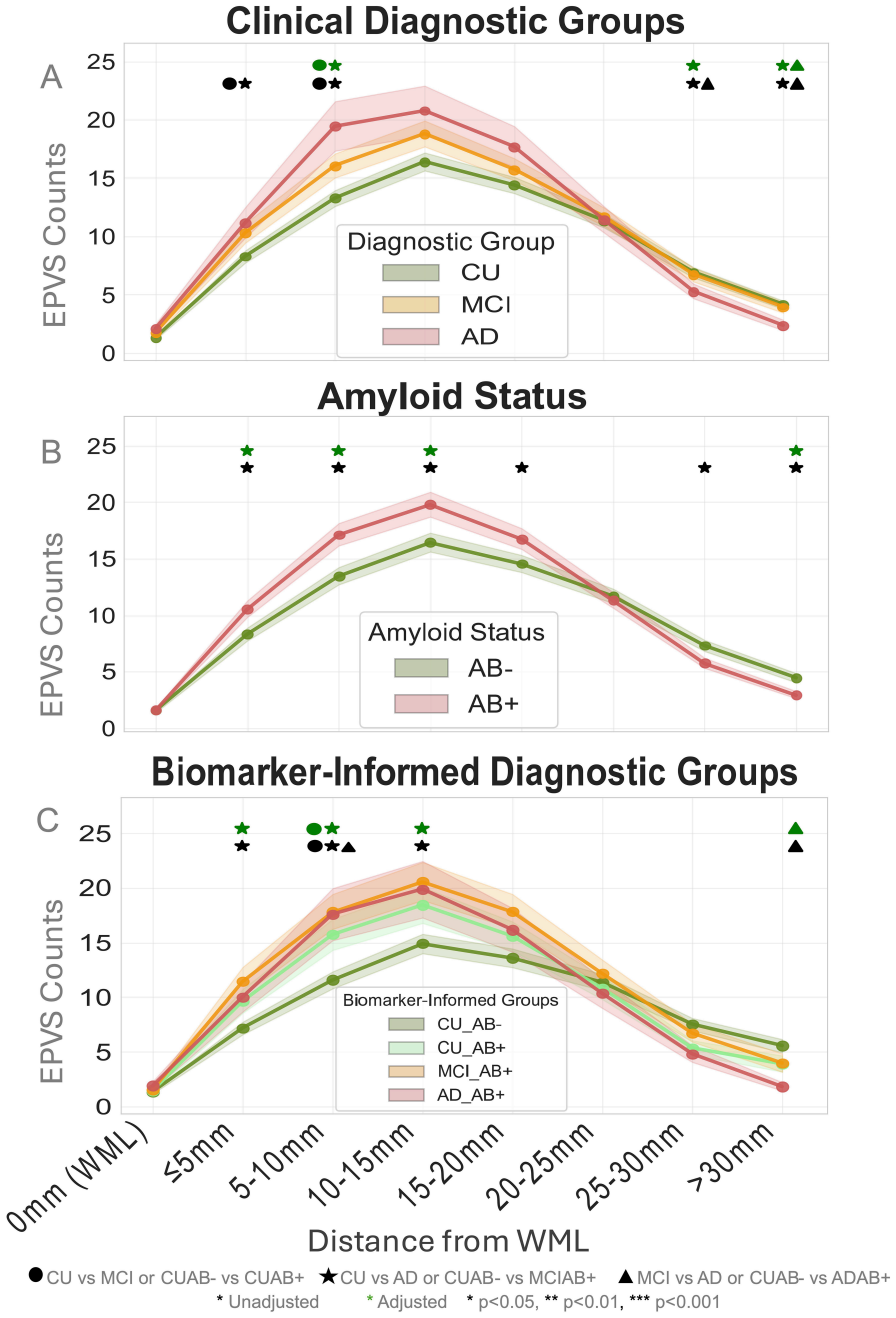
Distributions of EPVS counts per distance bins ranging from 0 mm (in WML) to 30 mm+ in increments of 5 mm between diagnostic groups **(A)**, amyloid status **(B)**, and biomarker-informed diagnostic groups **(C)**. Significant stars show adjusted comparison between subsequent groups and baseline in a particular distance category (i.e., against CU, CUAβ–, or Aβ–; also denoted in the figure legend). There was greater EPVS burden in the 5–15 mm range in the CUAβ+/MCIAβ+/Aβ+ groups and less EPVS burden at >30 mm regions in the AD/ADAβ+/Aβ+ groups. It is interesting to note the upside-down U shape, mostly skewed towards the 5–15 mm mark and exhibiting a characteristic drop off at the 20 mm mark. There were no differences around 15–25 mm.

**Figure 7:**
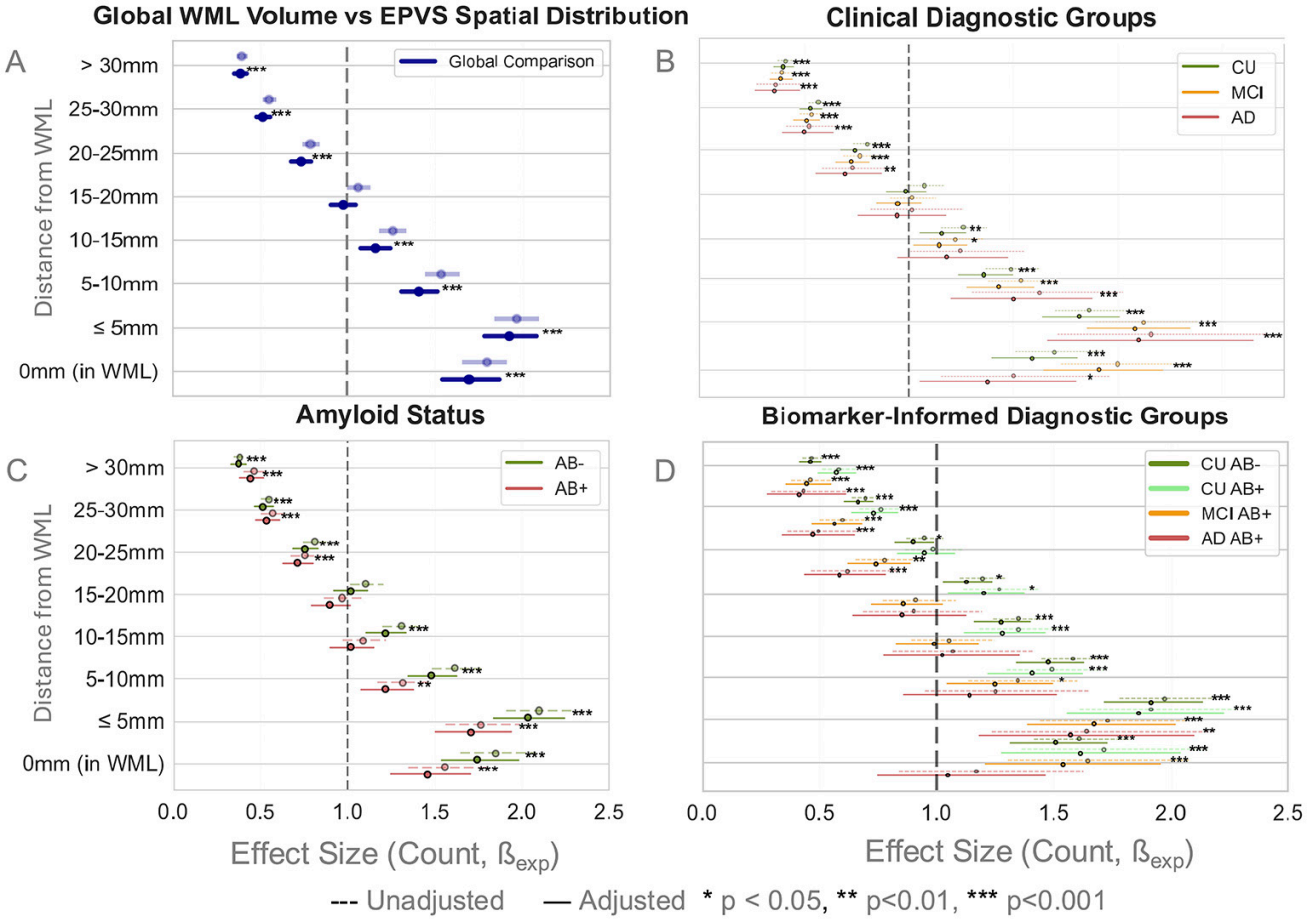
Forest plots show the effect sizes of the coefficients of WML as a function of EPVS counts across distance bins. Significance stars specifically refer to adjusted within-group significance of the association between EPVS counts at a particular distance and WML volume. **(A)** A significant relationship is observed between WML burden and EPVS counts in each distance bin, with decreasing coefficient size as distance increases. **(B–D)** No significant differences are observed across groups in the relationship of WML burden to EPVS counts in any distance bin. However, there were significant associations of WML volume to EPVS counts in each distance bin within the CU, Aβ–, and CUAβ– groups.

For the following comparisons, we report the ratio of expected counts for EPVS counts, standardized beta coefficients for EPVS volumes, and their respective CIs and *p*-values for the adjusted analyses (i.e., correcting for age and sex effects). Results for both unadjusted and adjusted models can be found in Supplementary Tables S2–S7. Visual comparisons of both unadjusted and adjusted are provided in Figures 3–7; significance stars in the figures specifically refer to comparisons or groups that are significant after age and sex adjustment unless stated otherwise.

### 3.3 Group comparisons of EPVS burden

#### 3.3.1 Clinical diagnosis

We first examined global and regional EPVS counts and volumes across clinical and biomarker staging groups; full results can be found in Supplementary Table S2A. In the BG, EPVS counts were significantly higher in MCI participants compared to CU (β_exp_ = 1.23, CI = [1.03, 1.46], *p* < 0.001). Within WML regions, EPVS volumes were higher in MCI compared to CU (β = 0.54, CI = [0.48, 0.59], *p* < 0.001) but, notably, lower in AD compared to MCI (β = −0.36, CI = [−0.44, −0.27], *p* < 0.001). There were significant differences between CU and AD in WML as well, but these differences did not survive adjustment for age and sex. No differences were observed in other regions. Results are illustrated in Figure 3A.

#### 3.3.2 Amyloid status

When stratified by amyloid status, significant differences were restricted to the WML region, where Aβ+ individuals exhibited greater EPVS volume compared to Aβ– individuals (β = 0.36, CI = [0.12, 0.59], *p* = 0.026; Figure 3B; Supplementary Table S2B). No differences were observed in other regions.

#### 3.3.3 Biomarker-informed disease stages

Using biomarker-informed stratification, the MCIAβ+ individuals exhibited greater EPVS counts in the BG (β_exp_ = 1.34, CI = [1.1, 1.62], *p* = 0.05) compared to the CUAβ– individuals as well as greater EPVS volume within WML compared to both CUAβ– (β = 0.58, CI = [0.51,0.65], *p* < 0.001) and CUAβ+ individuals (β = 0.55 CI = [0.45, 0.65], *p* < 0.001; Supplementary Table S2C). Consistent with the clinical group findings, the ADAβ+ individuals showed lower EPVS volumes within WML compared to MCIAβ+ individuals (β = −0.46, CI = [−0.58, −0.35], *p* < 0.001). Results are illustrated in Figure 3C, and full results are in Supplementary Table S2C.

### 3.4 Global relationship between EPVS and WML

We assessed whether the relationship between EPVS and WML burden varied by disease stages. Across the entire cohort, WML volume was positively associated with EPVS counts (β_exp_ = 1.16, CI = [1.09, 1.23], *p* < 0.001) and EPVS volumes (β = 0.09, CI = [0.03, 0.16], *p* = 0.006) in the BG as well as within the WML region itself (count: β_exp_ = 1.67, CI = [1.49, 1.87], *p* < 0.001, volume: β = 0.12, CI = [0.05, 0.19], *p* = 0.001; Figure 4A). While some associations were observed within groups (Figures 4B–D), we observed no significant interaction effects between WML volume and clinical, amyloid, or biomarker-informed groups (Supplementary Tables S3A–D; Supplementary Figure S2).

### 3.5 Spatial topography of EPVS relative to WML

#### 3.5.1 Proximity analysis (within, near, far)

In the proximity analysis, MCIAβ+ individuals exhibited a significantly higher EPVS burden “Near WML” ( ≤ 5 mm) compared to the CUAβ– individuals (β_exp_ = 1.23, CI = [1.03, 1.46], *p* = 0.05). Similarly, Aβ+ individuals showed greater EPVS burden “Near WML” compared to Aβ– individuals (β_exp_ = 1.45, CI = [1.12, 1.88], *p* = 0.02) (Figure 5). However, in the sensitivity analyses adjusting for total WML volume, these specific proximity effects were attenuated and no longer statistically significant (Supplementary Table S4).

#### 3.5.2 Granular distance analysis

##### 3.5.2.1 Across clinical diagnosis

As shown in Figure 6A, a greater number of EPVS were found in the 10 mm bin in both MCI and AD groups compared to CU (MCI: β_exp_ = 1.15, CI = [1.02, 1.39], *p* = 0.04; AD: β_exp_ = 1.29, CI = [1.03, 1.64], *p* = 0.04). However, in more distal regions, there were fewer EPVS found at the 30 mm and >30 mm distance bins in AD compared to CU (30 mm: β_exp_ = 0.69, CI = [0.50, 0.93], *p* = 0.049; >30 mm: β_exp_ = 0.45, CI = [0.30, 0.67], *p* < 0.001) and in MCI compared to CU (β_exp_ = 0.46, CI = [0.28, 0.73], *p* = 0.001).

##### 3.5.2.2 Across amyloid status

Aβ+ individuals had significantly more EPVS at intermediate distances: at 5 mm (β_exp_ = 1.23, CI = [1.06, 1.51], *p* = 0.02), 10 mm (β_exp_ = 1.24, CI = [1.09, 1.48], *p* = 0.004), and 15 mm (β_exp_ = 1.16, CI = [1.05, 1.37], *p* = 0.03) distance bins compared to Aβ– individuals, but fewer EPVS at distal locations (>30 mm bin: β_exp_ = 0.74, CI = [0.51, 0.85], *p* = 0.02); this is illustrated in Figure 6B (Supplementary Table S5B).

##### 3.5.2.3 Across biomarker-informed disease groups

As shown in Figure 6C, the MCIAβ+ individuals showed the greatest differences across all distance bins compared to CUAβ–. Specifically, MCIAβ+ individuals had higher EPVS counts at 5 mm (β_exp_ = 1.45, CI = [1.13, 1.91], *p* = 0.03) and 10 mm (β_exp_ = 1.42, CI = [1.16, 1.79], *p* = 0.01) distance bins compared to CUAβ–. CUAβ+ individuals also had higher EPVS counts at the 10 mm bin compared to CUAβ– (β_exp_ = 1.32, CI = [1.07, 1.65], *p* = 0.03). In contrast, the ADAβ+ individuals had significantly fewer EPVS at distal ranges (>30 mm) compared to CUAβ– (β_exp_ = 0.38, CI = [0.23, 0.60], *p* < 0.001). There were also a greater number of EPVS in ADAβ+ at 10 mm compared to CUAβ– and greater EPVS in MCIAβ+ at 15 mm compared to CUAβ–, as well as fewer EPVS in CUAβ+ and ADAβ+ at 25 mm, but these differences did not survive adjustment for age and sex (Supplementary Table S5C).

Across the group comparisons, in the sensitivity analyses adjusting for total WML volume, the only effect that remained significant was the difference between EPVS counts in Aβ– vs. Aβ+ at 10 mm; otherwise, these proximity effects were attenuated and no longer statistically significant (Supplementary Table S5).

#### 3.5.3 Influence of WML burden on spatial distribution

Total WML volume was significantly associated with EPVS counts across all distance bins except at 20 mm (*p* < 0.001; Figure 7), with the strength of this association diminishing as distance from the lesion increased. This stayed relatively consistent across clinical, amyloid, and biomarker-informed groups (Figure 7). No statistically significant differences were found across group comparisons (Supplementary Table S6).

## 4 Discussion

In this study, we leveraged deep learning-based segmentation to characterize the global and spatial coupling of EPVS and WML across the AD continuum. Following our aims, our primary findings showed: (1) EPVS burden is elevated in MCI and MCIAβ+ individuals compared to CU and CUAβ– groups, particularly within WML and basal ganglia regions; (2) global volumetric associations between EPVS and WML remained stable across groups, (3) local spatial coupling is significantly altered in early AD stages; specifically, MCIAβ+ and Aβ+ individuals exhibited a dense clustering of EPVS within the immediate vicinity of WML (5–10 mm for MCIAβ+, 5–15 mm for Aβ+), whereas AD and Aβ+ individuals showed fewer EPVS in distal white matter (30 mm+ for ADAβ+, 25–30+ mm for Aβ+); and 4) WML volume significantly associates with local EPVS burden, with reduced influence on EPVS burden far away from WML. We confirm our hypothesis that EPVS and WML exhibit varying spatial relationships across disease stages, with the changes manifesting in strong spatial correlations at early disease stages followed by decoupling of EPVS and WML at later disease stages. Taken together, these findings suggest that alterations in glymphatic-vascular coupling may serve as an early, spatially specific biomarker of AD pathology.

Consistent with prior studies, we found no significant differences in whole-brain EPVS burden across diagnostic groups (Younes et al., 2023; Menze et al., 2024; Jeong et al., 2022), suggesting that gross volumetric changes in late-stage disease are largely attributable to demographic factors. However, within WML and BG regions, EPVS burden was significantly higher in MCI/MCIAβ+ vs. CU/CUAβ– but paradoxically lower in AD/ADAβ+ vs. MCI/MCIAβ+. The elevation in MCI suggests that perivascular space enlargement may initially serve as a compensatory response to maintaining glymphatic clearance amidst emerging amyloid pathology or vascular stiffening; this pre-dementia pattern was also found in other studies (Menze et al., 2024; Ong et al., 2025; Goodman et al., 2024).

In contrast, the reduced EPVS burden in the AD phase compared to MCI may reflect the dynamic nature of these biomarkers, where visible enlargement represents an intermediate state of neurovascular failure before potential collapse or occlusion in advanced dementia. This difference may also stem from MCI group heterogeneity (Maillard et al., 2024; Potter et al., 2015b), wherein only 10–15% convert to AD on average (Risacher et al., 2009), or the fact that MCIAβ+ individuals with dementia risk may have vascular co-pathology alongside AD (Goodman et al., 2024), leading to greater EPVS burden compared to AD. A longitudinal study would be needed to untangle these temporal dynamics.

Our finding that these changes are specific to EPVS within WML and the BG region is consistent with existing literature. EPVS within the BG relates more closely to vascular biomarkers (Hong et al., 2024; Wu et al., 2025; Risacher et al., 2009) likely due to their proximity to major lenticulostriate arteries (Wardlaw et al., 2020). The positive association between WML—a predominantly vascular-driven pathology—and BG EPVS confirms the presence of vascular co-pathology in AD and supports the view that vascular contributions to AD pathology, such as those detailed the Vascular Contributions to Cognitive Impairment Dementia (VCID) model (Liu et al., 2021; Zlokovic et al., 2020; Oltmer et al., 2024), is a significant driver of perivascular remodeling even within a clinically diagnosed AD cohort. Even though greater EPVS within WML specifically could be explained by greater WML extent, group comparisons of WML across diagnostic categories (Supplementary Table S7) showed patterns inconsistent with this hypothesis, suggesting that these changes are specific to EPVS within WML.

Although we hypothesized that the global relationship between EPVS and WML might decouple across different disease stages, we observed no significant interaction effects across disease groups. This suggests that at a global scale, the association between EPVS and WML burden remains stable. However, previous studies suggest that EPVS and WML may be driven by AD-specific factors with distinct temporal trajectories (Shirzadi et al., 2023; Liu et al., 2025; Leone et al., 2025). Consequently, global association analyses may be too coarse to capture the localized, time-lagged interactions between fluid stagnation and lesion formation (Menze et al., 2024; Lao et al., 2025; Lee et al., 2016). This limitation necessitates the granular spatial analysis we performed.

A key novelty of this study is the characterization of the spatial coupling between EPVS and WML. We found a higher density of EPVS within 5–15 mm of WML in Aβ+ groups compared to Aβ– groups. When stratified by biomarker stage, this effect was driven primarily by the MCIAβ+ group, which exhibited significantly higher EPVS burden 5–10 mm from lesion boundaries. This distance range corresponds to a region identified in previous studies as the “WML penumbra” (approximately 2–9 mm away from WML) (Promjunyakul et al., 2016), which is characterized by diffuse white matter injury and subtle microstructural damage that often precedes visible lesion growth (Voorter et al., 2025; Maillard et al., 2011; Wang et al., 2023; Promjunyakul et al., 2016). The clustering of EPVS in this penumbral region likely reflects local interstitial fluid stagnation or compensatory drainage attempts in areas of high vascular vulnerability (Barnes et al., 2022; Huang et al., 2021). The fact that this spatial signature is prominent in MCIAβ+ but less distinct in ADAβ+ supports the utility of spatial EPVS metrics as early biomarkers of neurovascular instability. Furthermore, this aligns with histological evidence showing EPVS emerging in areas of amyloid deposition (Perosa et al., 2022), suggesting that amyloid burden may mediate the spatial coupling of EPVS and WML, indicators of clearance failure and white matter injury (Barnes et al., 2022; Hong et al., 2024; Francis et al., 2019; Huang et al., 2021; Huo et al., 2023).

The observation of significantly fewer EPVS at distal ranges (>30 mm) in ADAβ+ compared to CUAβ– is intriguing. One multi-center study reported a pattern of decreased EPVS counts with increased EPVS volume, where fewer but larger diameter EPVS predicted higher dementia risk (Barisano et al., 2025). However, in a supplementary analysis (Supplementary Figure S1), we found that both EPVS count and volume were lower in the ADAβ+ group compared to CUAβ−, a pattern sustained when comparing Aβ+ and Aβ– groups. This suggests a more complex picture that aligns with our global volumetric finding of reduced EPVS in AD compared to MCI. We offer three possible explanations for this decrease. First, cortical and regional atrophy may reduce the tissue volume available to harbor visible PVS. While our results persisted after adjustment for regional BG and WM volumes (Supplementary Table S2), subtle local atrophy not captured by these measures could contribute to these results. Second, those predisposed to AD may have lower baseline EPVS. A recent study on autosomal-dominant AD found lower EPVS count fractions in mutation carriers vs. non-carriers (Leone et al., 2025), suggesting that AD-diagnosed individuals may be more susceptible to pre-existing vascular and glymphatic deficits resulting in less baseline EPVS. Additionally, severe arterial stiffening in late-stage disease may prevent the pulsatile expansion required to maintain a patent, MRI-visible space. Third, perivascular obstruction by protein aggregation may render EPVS invisible on MRI despite underlying pathology. While the Intramural Periarterial Drainage (IPAD) and glymphatic hypotheses (Agarwal et al., 2024; Carare et al., 2008; Hawkes et al., 2014; Morris et al., 2014) suggest that amyloid deposition obstructs clearance pathways, the radiological manifestation likely evolves with disease severity. In early stages, partial obstruction may lead to fluid backup and visible PVS dilation (as seen in our MCI group). However, in advanced AD, dense accumulation of amyloid-beta within the perivascular space (such as in CAA) may displace the interstitial fluid necessary for MRI visibility, causing a pathologically compromised vessel to appear normal. Supporting this, we found fewer distal EPVS in Aβ+ vs. Aβ– participants (Figure 5B, Supplementary Table 5B). While histological studies (Perosa et al., 2022) reveal amyloid deposits within EPVS using ultrahigh resolution imaging, clinical-resolution MRI captures only macroscopic changes, potentially explaining these differences. However, this interpretation remains speculative; longitudinal studies are needed to fully untangle these patterns in AD progression.

Some of the distance differences between groups may be driven by age-related processes. In the 0 mm (within WML) to 10 mm regions, there were differences in CU vs. MCI or CU vs. AD groups; however, these differences did not survive adjustment for age and sex. Previous studies have found age-related changes in both EPVS and WML (Barisano et al., 2025; Lynch et al., 2023; Erten-Lyons et al., 2013), and these results may be an indication of their spatial relationship in the aging process. This is also consistent with previous studies that have examined the topological relationship between EPVS and WML (Barnes et al., 2022; Huo et al., 2023; Jack et al., 2024a).

We also examined whether total WML burden influenced the spatial distribution of EPVS. We found that WML volume was strongly associated with EPVS counts across all distance bins, though the strength of this association diminished as the distance from the lesion increased (Figure 7). This implies that WMLs might exert a field of influence—likely related to localized edema or fluid stagnation—that promotes EPVS formation in their immediate vicinity. Importantly, this relationship did not differ significantly across disease groups, suggesting that the physical coupling between lesion load and perivascular space enlargement is a fundamental feature of vascular co-pathology that persists regardless of AD diagnosis (Hong et al., 2024; Gouveia-Freitas and Bastos-Leite, 2021; Wang et al., 2021; Maillard et al., 2024; Potter et al., 2015b; Risacher et al., 2009; Liu et al., 2021; Zlokovic et al., 2020; Hawkes et al., 2014; Morris et al., 2014).

Our study has several limitations. First, EPVS segmentations were generated by an automated deep learning model, which, while demonstrating robust performance (Table 2), remains a surrogate for gold standard labels. Second, we included EPVS within WML in our training data to capture the full continuum of fluid pathology. While this allows for a more complete assessment, distinguishing EPVS from WML is radiologically challenging; our accuracy metrics for segmentation within WML is lower compared to other regions (Table 2) and has potential to be confounded by segmentations of visually similar structures such as lacunes (Wardlaw et al., 2020). We used dual-channel (T1w and FLAIR) inputs to maximize contrast, but results for this region remain exploratory. Third, the strong correlation between total WML volume and EPVS count introduces potential confounding in the spatial analysis—larger WMLs naturally have larger penumbras. While we attempted to correct for this by including WML volume as a covariate, this may over-adjust given the mechanistic link between these biomarkers. Notably, WML volumes were significantly greater in both MCI and AD groups compared to CU and MCI respectively—if the spatial extent of WML was the only feature that contributes to EPVS clustering near WML, then we should see more EPVS near WML following this pattern, but our results indicated a more complex relationship. Nevertheless, future studies might utilize EPVS density (count per unit volume of the distance shell) to better untangle these effects and include analyses of the distances for each individual cluster of WML (Barnes et al., 2022).

It is also important to acknowledge that MRI-visible EPVS and WML are surrogate markers rather than histological ground truths of specific pathophysiological mechanisms. These features are dynamic and multifactorial, potentially influenced by unmeasured factors beyond cerebral small vessel disease, such as neuroinflammation, transient fluid shifts, and axonal degeneration. WML, for example, is implicated in various diseases including stroke, multiple sclerosis, and AD independent of vascular factors (Shirzadi et al., 2023; Lee et al., 2016). Although ADNI excludes individuals with vascular dementia (Hachinski score > 4) at screening, the exclusion criteria is not comprehensive, limiting interpretation of the spatial associations observed.

While this study focused on AD pathology, the combination of vascular dysfunction and neurodegeneration frequently aligns with the broader framework of VCID. Mixed pathology is common along the AD continuum, and VCID mechanisms—such as arteriolosclerosis and blood-brain barrier leakage—likely contribute to the observed EPVS-WML coupling (Zlokovic et al., 2020). The strong spatial association between EPVS and WML in the BG suggests that small vessel disease pathology remains a potent driver of perivascular remodeling even in amyloid-positive individuals (Potter et al., 2015b; Risacher et al., 2009; Liu et al., 2021; Zlokovic et al., 2020; Oltmer et al., 2024). Future studies utilizing cohorts with pure AD pathology (e.g., autosomal dominant AD) vs. those with predominant vascular risk factors are needed to unravel the specific contributions of amyloid toxicity vs. ischemic injury to these spatial patterns. Including additional structures related to cognitive impairment, such as the hippocampus, may also strengthen the ability to differentiate pathological processes; in this study, our segmentation method showed reduced reliability (Dice < 0.5, Supplementary Table S1B) in anatomical regions with high partial volume effects and complex geometry and therefore excluded these additional structures.

Finally, future studies should integrate blood-based biomarkers to better contextualize the spatial coupling of EPVS and WML within the chronological framework of AD progression. Recent evidence suggests that plasma biomarkers, such as Glial Fibrillary Acidic Protein (GFAP) and Neurofilament Light chain (NfL), are sensitive to early neurovascular unit dysfunction and axonal injury (Wang et al., 2025; Raghavan et al., 2025). Correlating these fluid markers with the specific spatial topology of EPVS—particularly the clustering observed in the WML penumbra—could help determine whether perivascular space enlargement is a marker of downstream neurodegeneration or a secondary consequence of early vascular injury. Such multimodal analyses would be instrumental in validating spatial EPVS metrics as non-invasive biomarkers for early risk stratification.

## 5 Conclusion

Our study demonstrates that the spatial associations between EPVS and WML vary across the AD continuum. The specific enrichment of EPVS in the WML penumbra region in early-stage AD points to a critical window of neurovascular dysfunction. These findings suggest that spatial metrics may offer better sensitivity over global volume measures for detecting early glymphatic failure and investigating the neurovascular mechanisms driving cognitive decline.

## Data Availability

Data, including clinical, biofluid, genetic, imaging, and neuropathology results from ADNI participants, are shared with approved researchers through the LONI Image and Data Archive (IDA), a secure research data repository. Segmentation data used in this study may be available upon request.
