# Supplementary material for: Spatial coupling of enlarged perivascular spaces and white matter lesions across the Alzheimer's disease continuum
Source: Front Neurosci. 2026 Apr 1;20:1772024. doi: 10.3389/fnins.2026.1772024 (PMC13079328; doi:10.3389/fnins.2026.1772024)
Supplement: Supplementary file 1 [file Table_1.docx]

**Supplementary material**

**Table S1A. Demographics and Clinical Characteristics (with p-values for comparisons)**

|  | **Total** | **CU** | **MCI** | **AD** | **p-values** |
| --- | --- | --- | --- | --- | --- |
| **N** | 1080 | 593 | 369 | 118 | - |
| **Age (mean,std)** | 74.05, 8.26 | 73.02, 8.13 | 74.73, 8.11 | 77.12, 8.39 | a: p=0.000 b: p=0.002 c: p=0.006 |
| **Sex (n females, %)** | 566, 52.41% | 352, 59.36% | 163, 44.17% | 51, 43.22% | a: p=0.002 b: p=0.000 c: p=0.940 |
| **Education (>16) (n high, %)** | 763, 70.65% | 447, 75.38% | 252, 68.29% | 64, 54.24% | a: p=0.000 b: p=0.020 c: p=0.008 |
| **Race (NHW/NHB/HB/A/L/O, n, %)** | 839 (77.69%), 119 (11.02%), 59 (5.46%), 31 (2.87%), 14 (1.30%), 18 (1.67%), | 432 (72.85%), 78 (13.15%), 39 (6.58%), 24 (4.05%), 11 (1.85%), 9 (1.52%), | 308 (83.47%), 31 (8.40%), 17 (4.61%), 4 (1.08%), 3 (0.81%), 6 (1.63%), | 99 (83.90%), 10 (8.47%), 3 (2.54%), 3 (2.54%), 0.0 (0.00%), 3 (2.54%), | a: p=0.092 b: p=0.003 c: p=0.605 |
| **Modified Hachinski Score (0,1,2,3,4,5,6; n, %)** | 551 (51.02%), 458 (42.41%), 41 (3.80%), 21 (1.94%), 7 (0.65%), 1 (0.09%), 1 (0.09%), | 332 (55.99%), 238 (40.13%), 15 (2.53%), 7 (1.18%), 1 (0.17%), 0.0 (0.00%), 0.0 (0.00%), | 167 (45.26%), 170 (46.07%), 16 (4.34%), 9 (2.44%), 5 (1.36%), 1 (0.27%), 1 (0.27%), | 52 (44.07%), 50 (42.37%), 10 (8.47%), 5 (4.24%), 1 (0.85%), 0.0 (0.00%), 0.0 (0.00%), | a: p=0.001 b: p=0.003 c: p=0.544 |
| **Hypertension (n positive, %)** | 458, 42.41% | 235, 39.63% | 181, 49.05% | 62, 52.54% | a: p=0.013 b: p=0.005 c: p=0.579 |
| **Blood Pressure (sys>130 or dia>80, n, %)** | 656, 60.74% | 363, 61.21% | 223, 60.43% | 70, 59.32% | a: p=0.778 b: p=0.862 c: p=0.915 |
| **Amyloid positivity (n AB+, %)** | 361, 38.40% | 140, 26.52% | 139, 44.55% | 82, 82.00% | a: p=0.000 b: p=0.000 c: p=0.000 |
| **APOE4 Status (n E4+, %)** | 364, 37.64% | 174, 32.40% | 127, 37.69% | 63, 77.78% | a: p=0.000 b: p=0.055 c: p=0.000 |
| **Aβ+ Subgroups** |  | **CU_AB+** | **MCI_AB+** | **AD_AB+** | **p-values** |
| **N** |  | 140 | 139 | 82 | - |
| **Age (mean,std)** |  | 75.06, 7.87 | 75.73, 7.74 | 77.70, 8.85 | CUAß- vs CUAß+: p=0.005 CUAß- vs MCIAß+: p=0.000 CUAß- vs AD: p=0.000 CUAß+ vs MCIAß+: p=0.475 CUAß+ vs ADAß+: p=0.021 MCI vs ADAß+: p=0.085 |
| **Sex (n females, %)** |  | 84, 60.00% | 65, 46.76% | 35, 42.68% | CUAß- vs CUAß+: p=0.675 CUAß- vs MCIAß+: p=0.038 CUAß- vs ADAß+: p=0.020 CUAß+ vs MCIAß+: p=0.036 CUAß+ vs ADAß+: p=0.018 MCIAß+ vs ADAß+: p=0.654 |
| **Education (>16) (n high, %)** |  | 103, 73.57% | 93, 66.91% | 42, 51.22% | CUAß- vs CUAß+: p=0.475 CUAß- vs MCIAß+: p=0.025 CUAß- vs ADAß+: p=0.000 CUAß+ vs MCIAß+: p=0.277 CUAß+ vs ADAß+: p=0.001 MCIAß+ vs ADAß+: p=0.030 |
| **Race (NHW/NHB/HB/A/L/O, n, %)** |  | 109 (28.09%), 16 (4.12%), 8 (2.06%), 3 (0.77%), 0.0 (0.00%), 4 (1.03%), | 118 (84.89%), 12 (8.63%), 4 (2.88%), 0.0 (0.00%), 1 (0.72%), 4 (2.88%), | 70 (85.37%), 5 (6.10%), 3 (3.66%), 3 (3.66%), 0.0 (0.00%), 1 (1.22%), | CUAß- vs CUAß+: p=0.329 CUAß- vs MCI: p=0.063 CUAß- vs ADAß+: p=0.287 CUAß+ vs MCIAß+: p=0.521 CUAß+ vs ADAß+: p=0.491 MCIAß+ vs ADAß+: p=0.772 |
| **Modified Hachinski Score (0,1,2,3,4,5,6; n, %)** |  | 72 (51.43%), 62 (44.29%), 5 (3.57%), 1 (0.71%), 0.0 (0.00%), 0.0 (0.00%), 0.0 (0.00%), | 74 (53.24%), 55 (39.57%), 4 (2.88%), 5 (3.60%), 1 (0.72%), 0.0 (0.00%), 0.0 (0.00%), | 39 (47.56%), 34 (41.46%), 5 (6.10%), 4 (4.88%), 0.0 (0.00%), 0.0 (0.00%), 0.0 (0.00%), | CUAß- vs CUAß+: p=0.280 CUAß- vs MCIAß+: p=0.201 CUAß- vs ADAß+: p=0.018 CUAß+ vs MCIAß+: p=0.377 CUAß+ vs ADAß+: p=0.175 MCIAß+ vs ADAß+: p=0.648 |
| **Hypertension (n positive, %)** |  | 64, 45.71% | 58, 41.73% | 42, 51.22% | CUAß- vs CUAß+: p=0.082 CUAß- vs MCIAß+: p=0.361 CUAß- vs ADAß+: p=0.022 CUAß+ vs MCIAß+: p=0.582 CUAß+ vs ADAß+: p=0.514 MCIAß+ vs ADAß+: p=0.219 |
| **Blood Pressure (sys>130 or dia>80, n, %)** |  | 84, 60.00% | 78, 56.12% | 53, 64.63% | CUAß- vs CUAß+: p=0.901 CUAß- vs MCIAß+: p=0.355 CUAß- vs ADAß+: p=0.634 CUAß+ vs MCIAß+: p=0.592 CUAß+ vs ADAß+: p=0.587 MCIAß+ vs ADAß+: p=0.270 |
| **APOE4 Status (n E4+, %)** |  | 74, 54.01% | 127, 63.50% | 63, 67.07% | CUAß- vs CUAß+: p=0.000 CUAß- vs MCIAß+: p=0.000 CUAß- vs ADAß+: p=0.000 CUAß+ vs MCIAß+: p=0.141 CUAß+ vs ADAß+: p=0.079 MCIAß+ vs ADAß+: p=0.697 |

*Abbreviations: CU = Cognitively Unimpaired; MCI = Mild Cognitive Impairment; AD = Alzheimer's Disease; Aβ = Amyloid-beta; NHW = Non-Hispanic White; NHB = Non-Hispanic Black; HW = Hispanic White; A = Asian; L = Latino; O = Other; EPVS = Enlarged Perivascular Spaces; WMH = White Matter Hyperintensities; SD = Standard Deviation.*

*Note: p-values represent pairwise comparisons. For main diagnostic groups: a = CU vs MCI, b = CU vs AD, c = MCI vs AD. All pairwise comparisons are listed when applicable. Multiple-comparisons p-values are FDR adjusted.*

**Supplementary table S1B. Model Performance Metrics**

|  | **Segmentation Metrics** | | | | | **EPVS Count Correlation** | | **EPVS Volume Correlation** | |
| --- | --- | --- | --- | --- | --- | --- | --- | --- | --- |
| **Group** | **Precision** | **Recall** | **Overall Dice** | **Voxel-Wise Dice** | **Lesion-Wise Dice** | **Spearman ρ (p)** | **Lin's CCC** | **Spearman ρ (p)** | **Lin's CCC** |
| **Overall Dataset** |  |  |  |  |  |  |  |  |  |
| Validation Set (n=50) | 0.66 ± 0.17 | 0.61 ± 0.16 | 0.60 ± 0.10 | - | - | - | - | - | - |
| Test Set (n=10) | 0.66 ± 0.17 | 0.62 ± 0.20 | 0.61 ± 0.12 | 0.74 ± 0.07 | 0.63 ± 0.11 | 0.85, p=0.001 | 0.57 | 0.88, p<0.001 | 0.75 |
| **Region** |  |  |  |  |  |  |  |  |  |
| WM (n=10) | 0.66 ± 0.22 | 0.66 ± 0.21 | 0.63 ± 0.13 | 0.82 ± 0.10 | 0.62 ± 0.14 | 0.97, p<0.001 | 0.95 | 0.97, p<0.001 | 0.88 |
| BG (n=10) | 0.65 ± 0.18 | 0.53 ± 0.23 | 0.56 ± 0.15 | 0.72 ± 0.09 | 0.61 ± 0.12 | 0.93, p<0.001 | 0.94 | 0.96, p<0.001 | 0.91 |
| WML (n=3) | 0.66 ± 0.31 | 0.51 ± 0.32 | 0.51 ± 0.25 | 0.69 ± 0.37 | 0.41 ± 0.09 | 0.82, p<0.001 | 0.88 | 0.82, p<0.001 | 0.77 |
| nonWML (n=10) | 0.66 ± 0.16 | 0.62 ± 0.20 | 0.61 ± 0.12 | 0.76 ± 0.06 | 0.47 ± 0.11 | 0.97, p<0.001 | 0.95 | 0.97, p<0.001 | 0.88 |
| Hippocampus (n=4) | 0.00 | 0.00 | 0.00 | - | - | - | - | - | - |
| **Diagnostic Group** |  |  |  |  |  |  |  |  |  |
| CU (n=20) | 0.74 ± 0.20 | 0.67 ± 0.21 | 0.69 ± 0.19 | 0.81 ± 0.20 | 0.73 ± 0.15 | 0.96, p<0.001 | 0.91 | 0.94, p<0.001 | 0.94 |
| MCI (n=10) | 0.71 ± 0.20 | 0.66 ± 0.20 | 0.67 ± 0.17 | 0.76 ± 0.19 | 0.72 ± 0.10 | 0.98, p<0.001 | 0.97 | 0.95, p<0.001 | 0.81 |
| AD (n=10) | 0.74 ± 0.12 | 0.70 ± 0.11 | 0.72 ± 0.10 | 0.84 ± 0.07 | 0.69 ± 0.20 | 0.98, p<0.001 | 0.92 | 0.98, p<0.001 | 0.95 |

*Abbreviations: EPVS = Enlarged Perivascular Spaces; CCC = Concordance Correlation Coefficient; WM = White Matter; BG = Basal Ganglia; WMH = White Matter Hyperintensities; CU = Cognitively Unimpaired; MCI = Mild Cognitive Impairment; AD = Alzheimer's Disease.*

*Note: Values are presented as mean ± standard deviation unless otherwise specified. Spearman correlation coefficients (ρ) are shown with corresponding p-values. Lin's Concordance Correlation Coefficient (CCC) measures agreement between automated and manual segmentation.*

**The following convention is used across Supplementary tables 2-8:**

Subscripts of wm=white matter, gm = basal ganglia, wml or nonwml = white matter lesion; otherwise, whole brain region. ES = effect size. Adj = adjusted for age and sex, unadj = unadjusted, i.e. not adjusted for age and sex. CI = confidence interval. Sig = significance. Icvnorm = EPVS volume, pvscount = EPVS count. P= p-value

Diagnostic group comparisons include sensitivity analysis for tissue volume (wm+bg), denoted as “vol_adj”, as well as APOE-e4 and education adjusted, denoted as such (education = edu). Distance analysis is WML volume adjusted (wml vol adj).

**S2A: Diagnostic Group Comparisons**

| **Measure** | **Comparison** | **ES Unadj** | **CI Unadj lower** | **CI Unadj upper** | **p Unadj** | **ES Adj** | **CI Adj** | **CI Adj upper** | **p Adj** | **FDR p Unadj** | **FDR p Adj** | **FDR p (vol adj)** | **FDR p (APOEe4 + edu adj)** |
| --- | --- | --- | --- | --- | --- | --- | --- | --- | --- | --- | --- | --- | --- |
| pvscount | CU vs MCI | 1.13 | 1.00 | 1.28 | 0.06 | 1.07 | 0.95 | 1.22 | 0.27 | 0.14 | 0.77 | 0.40 | 0.63 |
| pvscount | CU vs AD | 1.20 | 0.99 | 1.44 | 0.06 | 1.07 | 0.89 | 1.30 | 0.46 | 0.14 | 0.77 | 0.44 | 0.83 |
| pvscount | MCI vs AD | 1.06 | 0.86 | 1.30 | 0.58 | 1.01 | 0.82 | 1.23 | 0.95 | 0.58 | 0.95 | 0.44 | 0.83 |
| pvscountwm | CU vs MCI | 1.11 | 0.97 | 1.27 | 0.13 | 1.06 | 0.93 | 1.21 | 0.40 | 0.22 | 0.77 | 0.44 | 0.83 |
| pvscountwm | CU vs AD | 1.21 | 0.99 | 1.48 | 0.06 | 1.09 | 0.89 | 1.34 | 0.40 | 0.14 | 0.77 | 0.44 | 0.83 |
| pvscountwm | MCI vs AD | 1.09 | 0.88 | 1.35 | 0.43 | 1.04 | 0.84 | 1.29 | 0.72 | 0.50 | 0.88 | 0.44 | 0.83 |
| pvscountgm | CU vs MCI | 1.39 | 1.20 | 1.59 | 3.73e-6 | 1.23 | 1.03 | 1.46 | 2.31e-4 | 3.73e-5 | 2.31e-3 | 8.16e-4 | 0.00 |
| pvscountgm | CU vs AD | 1.27 | 1.00 | 1.55 | 0.02 | 1.05 | 0.86 | 1.27 | 0.64 | 0.08 | 0.85 | 0.07 | 0.83 |
| pvscountgm | MCI vs AD | 0.91 | 0.73 | 1.14 | 0.43 | 0.82 | 0.66 | 1.01 | 0.06 | 0.50 | 0.39 | 0.60 | 0.27 |
| pvscountwmh | CU vs MCI | 1.34 | 1.00 | 1.71 | 0.02 | 1.24 | 0.97 | 1.58 | 0.09 | 0.08 | 0.43 | 0.33 | 0.63 |
| pvscountwmh | CU vs AD | 1.63 | 1.10 | 2.38 | 0.01 | 1.57 | 1.10 | 2.29 | 0.02 | 0.05 | 0.15 | 0.07 | 0.27 |
| pvscountwmh | MCI vs AD | 1.22 | 0.84 | 1.79 | 0.30 | 1.18 | 0.81 | 1.72 | 0.39 | 0.45 | 0.77 | 0.16 | 0.83 |
| pvscountnonwmh | CU vs MCI | 1.13 | 1.00 | 1.28 | 0.06 | 1.07 | 0.95 | 1.21 | 0.28 | 0.14 | 0.77 | 0.40 | 0.63 |
| pvscountnonwmh | CU vs AD | 1.20 | 0.99 | 1.44 | 0.06 | 1.07 | 0.89 | 1.30 | 0.46 | 0.14 | 0.77 | 0.44 | 0.83 |
| pvscountnonwmh | MCI vs AD | 1.06 | 0.86 | 1.30 | 0.58 | 1.01 | 0.82 | 1.23 | 0.95 | 0.58 | 0.95 | 0.44 | 0.83 |
| icvnormlog | CU vs MCI | 0.03 | -0.04 | 0.09 | 0.40 | 0.03 | -0.06 | 0.07 | 0.87 | 0.50 | 0.95 | 0.60 | 0.83 |
| icvnormlog | CU vs AD | 0.06 | -0.01 | 0.13 | 0.11 | 0.06 | -0.05 | 0.10 | 0.53 | 0.21 | 0.81 | 0.40 | 0.97 |
| icvnormlog | MCI vs AD | 0.04 | -0.05 | 0.13 | 0.36 | 0.04 | -0.07 | 0.11 | 0.65 | 0.48 | 0.85 | 0.40 | 0.83 |
| icvnormwmlog | CU vs MCI | 0.02 | -0.04 | 0.08 | 0.56 | 0.02 | -0.06 | 0.07 | 0.90 | 0.58 | 0.95 | 0.66 | 0.83 |
| icvnormwmlog | CU vs AD | 0.06 | -0.01 | 0.13 | 0.11 | 0.06 | -0.04 | 0.11 | 0.35 | 0.21 | 0.77 | 0.40 | 0.83 |
| icvnormwmlog | MCI vs AD | 0.05 | -0.04 | 0.14 | 0.27 | 0.05 | -0.06 | 0.12 | 0.45 | 0.43 | 0.77 | 0.40 | 0.83 |
| icvnormgmlog | CU vs MCI | 0.08 | 0.02 | 0.15 | 8.78e-3 | 0.08 | -0.01 | 0.11 | 0.12 | 0.05 | 0.46 | 0.14 | 0.63 |
| icvnormgmlog | CU vs AD | 0.09 | 0.02 | 0.17 | 0.01 | 0.09 | -0.04 | 0.10 | 0.42 | 0.06 | 0.77 | 0.12 | 0.83 |
| icvnormgmlog | MCI vs AD | 0.03 | -0.06 | 0.12 | 0.54 | 0.03 | -0.10 | 0.07 | 0.74 | 0.58 | 0.88 | 0.44 | 0.94 |
| icvnormwmhlog | CU vs MCI | 0.54 | 0.49 | 0.60 | 5.13e-89 | 0.54 | 0.48 | 0.59 | 2.22e-85 | 1.54e-87 | 6.65e-84 | 6.04e-85 | 0.00 |
| icvnormwmhlog | CU vs AD | 0.10 | 0.03 | 0.18 | 5.80e-3 | 0.10 | -0.01 | 0.13 | 0.11 | 0.04 | 0.46 | 0.11 | 0.63 |
| icvnormwmhlog | MCI vs AD | -0.36 | -0.44 | -0.28 | 2.95e-17 | -0.36 | -0.44 | -0.27 | 9.61e-17 | 4.42e-16 | 1.44e-15 | 1.39e-13 | 0.00 |
| icvnormnonwmhlog | CU vs MCI | 0.03 | -0.04 | 0.09 | 0.42 | 0.03 | -0.06 | 0.07 | 0.90 | 0.50 | 0.95 | 0.62 | 0.83 |
| icvnormnonwmhlog | CU vs AD | 0.06 | -0.01 | 0.13 | 0.12 | 0.06 | -0.05 | 0.10 | 0.54 | 0.21 | 0.81 | 0.40 | 0.97 |
| icvnormnonwmhlog | MCI vs AD | 0.04 | -0.05 | 0.13 | 0.35 | 0.04 | -0.07 | 0.11 | 0.64 | 0.48 | 0.85 | 0.40 | 0.83 |

**S2B: Amyloid group comparison**

| **Measure** | **Comparison** | **ES Unadj** | **CI Unadj lower** | **CI Unadj upper** | **p Unadj** | **ES Adj** | **CI Adj lower** | **CI Adj upper** | **p Adj** | **FDR p Unadj** | **FDR p Adj** | **FDR p (vol adj)** | **FDR p (APOEe4 + edu adj)** |
| --- | --- | --- | --- | --- | --- | --- | --- | --- | --- | --- | --- | --- | --- |
| pvscount | AB- vs AB+ | 1.13 | 0.99 | 1.28 | 0.07 | 1.09 | 0.96 | 1.24 | 0.18 | 0.08 | 0.30 | 0.18 | 0.32 |
| pvscountwm | AB- vs AB+ | 1.12 | 0.98 | 1.29 | 0.10 | 1.09 | 0.95 | 1.25 | 0.21 | 0.11 | 0.30 | 0.21 | 0.32 |
| pvscountgm | AB- vs AB+ | 1.17 | 1.01 | 1.34 | 0.03 | 1.07 | 0.94 | 1.22 | 0.31 | 0.07 | 0.39 | 0.13 | 0.32 |
| pvscountwmh | AB- vs AB+ | 1.06 | 0.83 | 1.36 | 0.63 | 1.05 | 0.82 | 1.33 | 0.70 | 0.63 | 0.70 | 0.41 | 0.72 |
| pvscountnonwmh | AB- vs AB+ | 1.13 | 0.99 | 1.28 | 0.07 | 1.09 | 0.96 | 1.24 | 0.18 | 0.08 | 0.30 | 0.18 | 0.32 |
| icvnormlog | AB- vs AB+ | 0.08 | -0.01 | 0.36 | 0.02 | 0.05 | -0.03 | 0.30 | 0.10 | 0.07 | 0.30 | 0.13 | 0.32 |
| icvnormwmlog | AB- vs AB+ | 0.06 | -0.01 | 0.37 | 0.05 | 0.05 | -0.04 | 0.32 | 0.13 | 0.08 | 0.30 | 0.17 | 0.32 |
| icvnormgmlog | AB- vs AB+ | 0.07 | -0.04 | 0.37 | 0.03 | 0.02 | -0.11 | 0.23 | 0.48 | 0.07 | 0.53 | 0.21 | 0.48 |
| icvnormwmhlog | AB- vs AB+ | 0.11 | 0.03 | 0.62 | 1.11e-3 | 0.36 | 0.12 | 0.59 | 2.65e-3 | 0.01 | 0.03 | 0.02 | 0.06 |
| icvnormnonwmhlog | AB- vs AB+ | 0.08 | -9.90e-3 | 0.37 | 0.02 | 0.05 | -0.03 | 0.31 | 0.10 | 0.07 | 0.30 | 0.13 | 0.32 |

**S2C: Biomarker-informed group comparison**

| **Measure** | **Comparison** | **ES Unadj** | **CI unadj lower** | **CI unadj upper** | **p Unadj** | **ES Adj** | **CI adj lower** | **CI adj upper** | **p Adj** | **FDR p Unadj** | **FDR p Adj** | **FDR p (vol adj)** | **FDR p (APOEe4 + edu adj)** |
| --- | --- | --- | --- | --- | --- | --- | --- | --- | --- | --- | --- | --- | --- |
| pvscount | CU_AB- vs CU_AB+ | 1.12 | 1.00 | 1.00 | 0.24 | 1.09 | 0.95 | 1.22 | 0.37 | 0.54 | 0.68 | 0.74 | 0.52 |
| pvscount | CU_AB- vs MCI_AB+ | 1.27 | 0.99 | 0.99 | 0.01 | 1.20 | 0.91 | 1.34 | 0.05 | 0.10 | 0.50 | 0.27 | 0.31 |
| pvscount | CU_AB- vs AD_AB+ | 1.12 | 0.86 | 0.86 | 0.34 | 0.99 | 0.89 | 1.35 | 0.96 | 0.56 | 0.99 | 0.89 | 0.75 |
| pvscount | CU_AB+ vs MCI_AB+ | 1.14 | 0.97 | 0.97 | 0.23 | 1.11 | 0.93 | 1.22 | 0.35 | 0.54 | 0.68 | 0.58 | 0.69 |
| pvscount | CU_AB+ vs AD_AB+ | 1.00 | 0.99 | 0.99 | 0.99 | 0.93 | 0.89 | 1.35 | 0.60 | 0.99 | 0.88 | 0.84 | 0.60 |
| pvscount | MCI_AB+ vs AD_AB+ | 0.88 | 0.88 | 0.88 | 0.33 | 0.85 | 0.89 | 1.38 | 0.22 | 0.56 | 0.68 | 0.74 | 0.41 |
| pvscountwm | CU_AB- vs CU_AB+ | 1.10 | 1.21 | 1.21 | 0.34 | 1.08 | 1.13 | 1.44 | 0.45 | 0.56 | 0.72 | 0.76 | 0.60 |
| pvscountwm | CU_AB- vs MCI_AB+ | 1.26 | 1.03 | 1.03 | 0.03 | 1.19 | 1.06 | 1.54 | 0.08 | 0.14 | 0.60 | 0.28 | 0.41 |
| pvscountwm | CU_AB- vs AD_AB+ | 1.11 | 0.73 | 0.73 | 0.41 | 0.99 | 0.87 | 1.31 | 0.96 | 0.56 | 0.99 | 0.99 | 0.76 |
| pvscountwm | CU_AB+ vs MCI_AB+ | 1.14 | 1.04 | 1.04 | 0.27 | 1.11 | 0.96 | 1.56 | 0.36 | 0.55 | 0.68 | 0.63 | 0.69 |
| pvscountwm | CU_AB+ vs AD_AB+ | 1.01 | 1.12 | 1.12 | 0.95 | 0.95 | 1.12 | 2.42 | 0.70 | 0.98 | 0.90 | 0.84 | 0.68 |
| pvscountwm | MCI_AB+ vs AD_AB+ | 0.88 | 0.84 | 0.84 | 0.39 | 0.86 | 1.00 | 2.15 | 0.29 | 0.56 | 0.68 | 0.76 | 0.41 |
| pvscountgm | CU_AB- vs CU_AB+ | 1.17 | 1.00 | 1.00 | 0.12 | 1.06 | 0.95 | 1.22 | 0.52 | 0.38 | 0.79 | 0.85 | 0.61 |
| pvscountgm | CU_AB- vs MCI_AB+ | 1.48 | 0.99 | 0.99 | 0.00 | 1.34 | 1.10 | 1.62 | 0.00 | 0.00 | 0.05 | 0.02 | 0.01 |
| pvscountgm | CU_AB- vs AD_AB+ | 1.32 | 0.86 | 0.86 | 0.03 | 1.04 | 0.89 | 1.35 | 0.71 | 0.14 | 0.90 | 0.27 | 0.56 |
| pvscountgm | CU_AB+ vs MCI_AB+ | 1.26 | -0.09 | -0.09 | 0.06 | 1.21 | -0.12 | 0.21 | 0.11 | 0.23 | 0.60 | 0.13 | 0.43 |
| pvscountgm | CU_AB+ vs AD_AB+ | 1.12 | -0.05 | -0.05 | 0.40 | 0.98 | -0.10 | 0.41 | 0.89 | 0.56 | 0.99 | 0.37 | 0.86 |
| pvscountgm | MCI_AB+ vs AD_AB+ | 0.89 | -0.14 | -0.14 | 0.42 | 0.81 | -0.10 | 0.43 | 0.12 | 0.56 | 0.60 | 0.85 | 0.41 |
| pvscountwmh | CU_AB- vs CU_AB+ | 1.11 | -0.13 | -0.13 | 0.58 | 1.09 | -0.14 | 0.23 | 0.65 | 0.71 | 0.90 | 0.76 | 0.76 |
| pvscountwmh | CU_AB- vs MCI_AB+ | 1.17 | -0.05 | -0.05 | 0.38 | 1.04 | -0.11 | 0.46 | 0.85 | 0.56 | 0.99 | 0.84 | 0.75 |
| pvscountwmh | CU_AB- vs AD_AB+ | 1.43 | -0.13 | -0.13 | 0.13 | 1.42 | -0.10 | 0.49 | 0.14 | 0.40 | 0.60 | 0.28 | 0.32 |
| pvscountwmh | CU_AB+ vs MCI_AB+ | 1.05 | 0.06 | 0.06 | 0.79 | 0.99 | 0.01 | 0.33 | 0.97 | 0.84 | 0.99 | 0.94 | 0.91 |
| pvscountwmh | CU_AB+ vs AD_AB+ | 1.31 | 0.07 | 0.07 | 0.25 | 1.26 | 0.03 | 0.51 | 0.33 | 0.54 | 0.68 | 0.48 | 0.74 |
| pvscountwmh | MCI_AB+ vs AD_AB+ | 1.22 | -0.20 | -0.20 | 0.39 | 1.21 | -0.14 | 0.38 | 0.41 | 0.56 | 0.70 | 0.58 | 0.89 |
| pvscountnonwmh | CU_AB- vs CU_AB+ | 1.12 | 1.89 | 1.89 | 0.24 | 1.09 | 1.89 | 2.31 | 0.37 | 0.54 | 0.68 | 0.74 | 0.52 |
| pvscountnonwmh | CU_AB- vs MCI_AB+ | 1.27 | 0.07 | 0.07 | 0.01 | 1.20 | 0.03 | 0.39 | 0.05 | 0.10 | 0.50 | 0.27 | 0.31 |
| pvscountnonwmh | CU_AB- vs AD_AB+ | 1.12 | -2.27 | -2.27 | 0.35 | 0.99 | -2.15 | -1.28 | 0.96 | 0.56 | 0.99 | 0.89 | 0.75 |
| pvscountnonwmh | CU_AB+ vs MCI_AB+ | 1.14 | -0.10 | -0.10 | 0.23 | 1.11 | -0.12 | 0.21 | 0.35 | 0.54 | 0.68 | 0.58 | 0.69 |
| pvscountnonwmh | CU_AB+ vs AD_AB+ | 1.00 | -0.05 | -0.05 | 0.99 | 0.93 | -0.10 | 0.41 | 0.60 | 0.99 | 0.88 | 0.84 | 0.60 |
| pvscountnonwmh | MCI_AB+ vs AD_AB+ | 0.88 | -0.14 | -0.14 | 0.33 | 0.85 | -0.10 | 0.43 | 0.22 | 0.56 | 0.68 | 0.74 | 0.41 |
| icvnormlog | CU_AB- vs CU_AB+ | 0.08 | 0.04 | 0.20 | 0.08 | 0.06 | -0.03 | 0.15 | 0.16 | 0.28 | 0.60 | 0.45 | 0.41 |
| icvnormlog | CU_AB- vs MCI_AB+ | 0.10 | 1.00 | 1.00 | 0.02 | 0.07 | 0.95 | 1.22 | 0.11 | 0.14 | 0.60 | 0.27 | 0.41 |
| icvnormlog | CU_AB- vs AD_AB+ | 0.04 | 0.99 | 0.99 | 0.40 | 0.00 | 0.91 | 1.34 | 0.93 | 0.56 | 0.99 | 0.84 | 0.86 |
| icvnormlog | CU_AB+ vs MCI_AB+ | 0.03 | 0.86 | 0.86 | 0.61 | 0.02 | 0.89 | 1.35 | 0.74 | 0.71 | 0.90 | 0.76 | 0.96 |
| icvnormlog | CU_AB+ vs AD_AB+ | -0.03 | 0.97 | 0.97 | 0.62 | -0.07 | 0.93 | 1.22 | 0.32 | 0.71 | 0.68 | 0.76 | 0.43 |
| icvnormlog | MCI_AB+ vs AD_AB+ | -0.06 | 0.99 | 0.99 | 0.38 | -0.08 | 0.89 | 1.35 | 0.26 | 0.56 | 0.68 | 0.76 | 0.41 |
| icvnormwmlog | CU_AB- vs CU_AB+ | 0.06 | 0.88 | 0.88 | 0.14 | 0.06 | 0.89 | 1.38 | 0.21 | 0.43 | 0.68 | 0.49 | 0.41 |
| icvnormwmlog | CU_AB- vs MCI_AB+ | 0.08 | 1.21 | 1.21 | 0.05 | 0.06 | 1.13 | 1.44 | 0.15 | 0.22 | 0.60 | 0.28 | 0.41 |
| icvnormwmlog | CU_AB- vs AD_AB+ | 0.03 | 1.03 | 1.03 | 0.48 | 0.00 | 1.06 | 1.54 | 0.99 | 0.61 | 0.99 | 0.85 | 0.78 |
| icvnormwmlog | CU_AB+ vs MCI_AB+ | 0.03 | 0.73 | 0.73 | 0.66 | 0.02 | 0.87 | 1.31 | 0.74 | 0.73 | 0.90 | 0.82 | 0.96 |
| icvnormwmlog | CU_AB+ vs AD_AB+ | -0.03 | 1.04 | 1.04 | 0.68 | -0.05 | 0.96 | 1.56 | 0.43 | 0.75 | 0.72 | 0.76 | 0.56 |
| icvnormwmlog | MCI_AB+ vs AD_AB+ | -0.05 | 1.12 | 1.12 | 0.45 | -0.06 | 1.12 | 2.42 | 0.36 | 0.59 | 0.68 | 0.76 | 0.52 |
| icvnormgmlog | CU_AB- vs CU_AB+ | 0.05 | 0.84 | 0.84 | 0.22 | 0.02 | 1.00 | 2.15 | 0.69 | 0.54 | 0.90 | 0.97 | 0.86 |
| icvnormgmlog | CU_AB- vs MCI_AB+ | 0.11 | 1.00 | 1.00 | 0.01 | 0.06 | 0.95 | 1.22 | 0.16 | 0.10 | 0.60 | 0.28 | 0.41 |
| icvnormgmlog | CU_AB- vs AD_AB+ | 0.10 | 0.99 | 0.99 | 0.02 | 0.03 | 0.91 | 1.34 | 0.45 | 0.14 | 0.72 | 0.28 | 0.60 |
| icvnormgmlog | CU_AB+ vs MCI_AB+ | 0.07 | 0.86 | 0.86 | 0.27 | 0.05 | 0.89 | 1.35 | 0.39 | 0.55 | 0.69 | 0.36 | 0.69 |
| icvnormgmlog | CU_AB+ vs AD_AB+ | 0.08 | -0.09 | -0.09 | 0.25 | 0.03 | -0.12 | 0.21 | 0.68 | 0.54 | 0.90 | 0.37 | 0.89 |
| icvnormgmlog | MCI_AB+ vs AD_AB+ | 0.01 | -0.05 | -0.05 | 0.87 | -0.02 | -0.10 | 0.41 | 0.71 | 0.92 | 0.90 | 0.85 | 0.75 |
| icvnormwmhlog | CU_AB- vs CU_AB+ | 0.03 | -0.14 | -0.14 | 0.49 | 0.00 | -0.10 | 0.43 | 0.95 | 0.61 | 0.99 | 0.94 | 0.96 |
| icvnormwmhlog | CU_AB- vs MCI_AB+ | 0.59 | -0.13 | -0.13 | 0.00 | 0.58 | -0.14 | 0.23 | 0.00 | 0.00 | 0.00 | 0.00 | 0.00 |
| icvnormwmhlog | CU_AB- vs AD_AB+ | 0.09 | -0.05 | -0.05 | 0.05 | 0.05 | -0.11 | 0.46 | 0.31 | 0.22 | 0.68 | 0.28 | 0.41 |
| icvnormwmhlog | CU_AB+ vs MCI_AB+ | 0.55 | -0.13 | -0.13 | 0.00 | 0.55 | -0.10 | 0.49 | 0.00 | 0.00 | 0.00 | 0.00 | 0.00 |
| icvnormwmhlog | CU_AB+ vs AD_AB+ | 0.08 | 0.06 | 0.06 | 0.21 | 0.07 | 0.01 | 0.33 | 0.33 | 0.54 | 0.68 | 0.28 | 0.74 |
| icvnormwmhlog | MCI_AB+ vs AD_AB+ | -0.47 | 0.07 | 0.07 | 0.00 | -0.46 | -0.58 | -0.35 | 0.00 | 0.00 | 0.00 | 0.00 | 0.00 |
| icvnormnonwmhlog | CU_AB- vs CU_AB+ | 0.08 | -0.20 | -0.20 | 0.08 | 0.06 | -0.14 | 0.38 | 0.15 | 0.28 | 0.60 | 0.45 | 0.41 |
| icvnormnonwmhlog | CU_AB- vs MCI_AB+ | 0.10 | 1.89 | 1.89 | 0.02 | 0.07 | 1.89 | 2.31 | 0.11 | 0.14 | 0.60 | 0.27 | 0.41 |
| icvnormnonwmhlog | CU_AB- vs AD_AB+ | 0.04 | 0.07 | 0.07 | 0.41 | 0.00 | 0.03 | 0.39 | 0.93 | 0.56 | 0.99 | 0.84 | 0.86 |
| icvnormnonwmhlog | CU_AB+ vs MCI_AB+ | 0.03 | -2.27 | -2.27 | 0.63 | 0.02 | -2.15 | -1.28 | 0.75 | 0.71 | 0.90 | 0.76 | 0.96 |
| icvnormnonwmhlog | CU_AB+ vs AD_AB+ | -0.03 | -0.10 | -0.10 | 0.61 | -0.07 | -0.12 | 0.21 | 0.32 | 0.71 | 0.68 | 0.76 | 0.43 |
| icvnormnonwmhlog | MCI_AB+ vs AD_AB+ | -0.06 | -0.05 | -0.05 | 0.38 | -0.08 | -0.10 | 0.41 | 0.26 | 0.56 | 0.68 | 0.76 | 0.41 |

**S3A: EPVS WMH All cohort association**

| **Lesion Type** | **Adjustment** | **p WMH** | **ES WMH** | **ES CI Lower WMH** | **ES CI Upper WMH** | **p Age** | **ES Age** | **ES CI Lower Age** | **ES CI Upper Age** | **p Sex** | **ES Sex** | **ES CI Lower Sex** | **ES CI Upper Sex** | **FDR p WMH** | **FDR p Age** | **FDR p Sex** | **P WMH (APOEe4 + edu adj)** |
| --- | --- | --- | --- | --- | --- | --- | --- | --- | --- | --- | --- | --- | --- | --- | --- | --- | --- |
| pvscount | unadjusted | 1.81e-4 | 1.10 | 1.05 | 1.15 | - | - | - | - | - | - | - | - | 5.18e-4 | - | - | 0.00 |
| icvnormlog | unadjusted | 5.51e-4 | 0.10 | 0.05 | 0.16 | - | - | - | - | - | - | - | - | 1.10e-3 | - | - | 0.00 |
| pvscountwm | unadjusted | 4.53e-3 | 1.07 | 1.02 | 1.13 | - | - | - | - | - | - | - | - | 6.98e-3 | - | - | 0.01 |
| icvnormwmlog | unadjusted | 0.07 | 0.06 | -4.12e-3 | 0.12 | - | - | - | - | - | - | - | - | 0.10 | - | - | 0.10 |
| pvscountgm | unadjusted | 4.88e-26 | 1.32 | 1.25 | 1.39 | - | - | - | - | - | - | - | - | 9.76e-25 | - | - | 0.00 |
| icvnormgmlog | unadjusted | 4.91e-16 | 0.24 | 0.18 | 0.30 | - | - | - | - | - | - | - | - | 2.45e-15 | - | - | 0.00 |
| pvscountwmh | unadjusted | 5.72e-25 | 1.76 | 1.58 | 1.96 | - | - | - | - | - | - | - | - | 5.72e-24 | - | - | 0.00 |
| icvnormwmhlog | unadjusted | 3.66e-6 | 0.14 | 0.08 | 0.20 | - | - | - | - | - | - | - | - | 1.22e-5 | - | - | 0.00 |
| pvscountnonwmh | unadjusted | 2.12e-4 | 1.10 | 1.04 | 1.15 | - | - | - | - | - | - | - | - | 5.31e-4 | - | - | 0.00 |
| icvnormnonwmhlog | unadjusted | 8.09e-4 | 0.10 | 0.04 | 0.16 | - | - | - | - | - | - | - | - | 1.47e-3 | - | - | 0.00 |
| pvscount | adjusted | 0.32 | 1.03 | 0.97 | 1.09 | 4.43e-5 | 1.02 | 1.01 | 1.03 | 4.69e-3 | 1.19 | 1.06 | 1.35 | 0.42 | 7.55e-5 | 0.02 | 0.25 |
| icvnormlog | adjusted | 0.44 | 0.03 | -0.04 | 0.09 | 3.89e-5 | 0.14 | 0.08 | 0.21 | 0.10 | 0.05 | -9.42e-3 | 0.11 | 0.52 | 7.55e-5 | 0.14 | 0.25 |
| pvscountwm | adjusted | 0.68 | 1.01 | 0.96 | 1.07 | 2.06e-4 | 1.02 | 1.01 | 1.02 | 3.41e-3 | 1.20 | 1.06 | 1.36 | 0.72 | 2.95e-4 | 0.02 | 0.50 |
| icvnormwmlog | adjusted | 0.96 | -1.60e-3 | -0.07 | 0.07 | 3.29e-3 | 0.10 | 0.03 | 0.17 | 0.16 | 0.04 | -0.02 | 0.10 | 0.96 | 4.12e-3 | 0.19 | 0.74 |
| pvscountgm | adjusted | 6.94e-7 | 1.16 | 1.09 | 1.23 | 1.64e-19 | 1.04 | 1.03 | 1.05 | 0.17 | 1.09 | 0.96 | 1.24 | 2.77e-6 | 1.64e-18 | 0.19 | 0.00 |
| icvnormgmlog | adjusted | 4.13e-3 | 0.09 | 0.03 | 0.16 | 1.19e-17 | 0.28 | 0.22 | 0.35 | 0.33 | 0.03 | -0.03 | 0.09 | 6.88e-3 | 5.93e-17 | 0.33 | 0.00 |
| pvscountwmh | adjusted | 6.29e-18 | 1.67 | 1.49 | 1.87 | 0.07 | 1.01 | 1.00 | 1.03 | 0.02 | 1.30 | 1.05 | 1.62 | 4.19e-17 | 0.08 | 0.04 | 0.00 |
| icvnormwmhlog | adjusted | 4.59e-4 | 0.12 | 0.05 | 0.19 | 0.52 | 0.02 | -0.05 | 0.09 | 0.09 | 0.05 | -7.69e-3 | 0.11 | 1.02e-3 | 0.52 | 0.14 | 0.00 |
| pvscountnonwmh | adjusted | 0.34 | 1.03 | 0.97 | 1.09 | 4.14e-5 | 1.02 | 1.01 | 1.03 | 4.46e-3 | 1.19 | 1.06 | 1.35 | 0.42 | 7.55e-5 | 0.02 | 0.25 |
| icvnormnonwmhlog | adjusted | 0.49 | 0.02 | -0.04 | 0.09 | 4.53e-5 | 0.14 | 0.07 | 0.21 | 0.09 | 0.05 | -8.87e-3 | 0.11 | 0.54 | 7.55e-5 | 0.14 | 0.26 |

**S3B1: EPVS WMH correlation in diagnostic groups: Main Effects**

| **Lesion Type** | **Adjustment** | **p WMH** | **ES WMH** | **ES CI Lower WMH** | **ES CI Upper WMH** | **p MCI** | **ES MCI** | **ES CI Lower MCI** | **ES CI Upper MCI** | **p AD** | **ES AD** | **ES CI Lower AD** | **ES CI Upper AD** | **FDR p WMH** | **FDR p MCI** | **FDR p AD** | **FDR p MCI (APOE e4 + edu adj)** | **FDR p AD (APOEe4 + edu adj)** |
| --- | --- | --- | --- | --- | --- | --- | --- | --- | --- | --- | --- | --- | --- | --- | --- | --- | --- | --- |
| pvscount | unadjusted | 4.93e-3 | 1.09 | 1.03 | 1.16 | 0.99 | 1.00 | 0.53 | 1.89 | 0.72 | 1.24 | 0.38 | 4.04 | 0.01 | 0.99 | 0.76 | 0.99 | 0.86 |
| icvnormlog | unadjusted | 3.04e-3 | 0.11 | 0.04 | 0.19 | 0.60 | -0.08 | -0.38 | 0.22 | 0.81 | -0.05 | -0.41 | 0.32 | 7.86e-3 | 0.60 | 0.99 | 0.61 | 0.99 |
| pvscountwm | unadjusted | 0.03 | 1.07 | 1.01 | 1.14 | 0.80 | 0.92 | 0.49 | 1.74 | 0.70 | 1.26 | 0.39 | 4.09 | 0.04 | 0.99 | 0.76 | 0.99 | 0.86 |
| icvnormwmlog | unadjusted | 0.10 | 0.06 | -0.01 | 0.14 | 0.60 | -0.08 | -0.38 | 0.22 | 0.92 | -0.02 | -0.39 | 0.35 | 0.15 | 0.60 | 0.99 | 0.61 | 0.99 |
| pvscountgm | unadjusted | 4.15e-12 | 1.26 | 1.18 | 1.35 | 0.03 | 2.11 | 1.09 | 4.08 | 0.35 | 1.79 | 0.53 | 6.00 | 2.08e-11 | 0.09 | 0.76 | 0.10 | 0.86 |
| icvnormgmlog | unadjusted | 2.12e-8 | 0.21 | 0.14 | 0.28 | 0.18 | 0.20 | -0.09 | 0.49 | 0.99 | -1.89e-3 | -0.36 | 0.35 | 2.12e-7 | 0.44 | 0.99 | 0.46 | 0.99 |
| pvscountwmh | unadjusted | 1.43e-12 | 1.63 | 1.42 | 1.86 | 0.04 | 4.26 | 1.08 | 16.73 | 0.76 | 0.71 | 0.08 | 6.53 | 1.43e-11 | 0.09 | 0.76 | 0.10 | 0.86 |
| icvnormwmhlog | unadjusted | 5.60e-4 | 0.11 | 0.05 | 0.18 | 0.02 | 0.30 | 0.04 | 0.55 | 0.55 | 0.09 | -0.21 | 0.40 | 2.80e-3 | 0.11 | 0.99 | 0.21 | 0.99 |
| pvscountnonwmh | unadjusted | 5.39e-3 | 1.09 | 1.03 | 1.16 | 0.99 | 1.00 | 0.53 | 1.89 | 0.73 | 1.23 | 0.38 | 4.01 | 0.01 | 0.99 | 0.76 | 0.99 | 0.86 |
| icvnormnonwmhlog | unadjusted | 3.84e-3 | 0.11 | 0.04 | 0.19 | 0.59 | -0.08 | -0.38 | 0.22 | 0.79 | -0.05 | -0.41 | 0.31 | 7.86e-3 | 0.60 | 0.99 | 0.61 | 0.99 |
| pvscount | adjusted | 0.45 | 1.03 | 0.96 | 1.10 | 0.98 | 1.01 | 0.53 | 1.91 | 0.70 | 1.26 | 0.39 | 4.09 | 0.52 | 0.99 | 0.76 | 0.99 | 0.86 |
| icvnormlog | adjusted | 0.35 | 0.04 | -0.04 | 0.12 | 0.58 | -0.08 | -0.38 | 0.21 | 0.74 | -0.06 | -0.42 | 0.30 | 0.42 | 0.60 | 0.99 | 0.61 | 0.99 |
| pvscountwm | adjusted | 0.71 | 1.01 | 0.95 | 1.08 | 0.83 | 0.93 | 0.49 | 1.77 | 0.69 | 1.27 | 0.39 | 4.14 | 0.71 | 0.99 | 0.76 | 0.99 | 0.86 |
| icvnormwmlog | adjusted | 0.83 | 8.95e-3 | -0.07 | 0.09 | 0.58 | -0.08 | -0.38 | 0.22 | 0.87 | -0.03 | -0.39 | 0.33 | 0.83 | 0.60 | 0.99 | 0.61 | 0.99 |
| pvscountgm | adjusted | 6.44e-3 | 1.10 | 1.03 | 1.19 | 0.01 | 2.28 | 1.18 | 4.41 | 0.35 | 1.78 | 0.53 | 6.00 | 0.01 | 0.09 | 0.76 | 0.10 | 0.86 |
| icvnormgmlog | adjusted | 0.08 | 0.07 | -8.75e-3 | 0.15 | 0.16 | 0.20 | -0.08 | 0.48 | 0.82 | -0.04 | -0.38 | 0.30 | 0.14 | 0.44 | 0.99 | 0.46 | 0.99 |
| pvscountwmh | adjusted | 1.06e-8 | 1.52 | 1.32 | 1.75 | 0.02 | 5.04 | 1.28 | 19.88 | 0.68 | 0.63 | 0.07 | 5.80 | 3.53e-8 | 0.09 | 0.76 | 0.10 | 0.86 |
| icvnormwmhlog | adjusted | 3.93e-3 | 0.10 | 0.03 | 0.17 | 0.02 | 0.30 | 0.05 | 0.55 | 0.57 | 0.09 | -0.22 | 0.40 | 7.86e-3 | 0.11 | 0.99 | 0.42 | 0.99 |
| pvscountnonwmh | adjusted | 0.47 | 1.03 | 0.96 | 1.10 | 0.98 | 1.01 | 0.53 | 1.91 | 0.71 | 1.25 | 0.38 | 4.07 | 0.52 | 0.99 | 0.76 | 0.99 | 0.86 |
| icvnormnonwmhlog | adjusted | 0.38 | 0.04 | -0.05 | 0.12 | 0.57 | -0.08 | -0.38 | 0.21 | 0.72 | -0.07 | -0.43 | 0.30 | 0.42 | 0.60 | 0.99 | 0.61 | 0.99 |

**S3B2: EPVS WMH correlation: diagnostic group interaction effects**

| **Lesion Type** | **Adjustment** | **p WMH×MCI** | **ES WMH×MCI** | **ES CI Lower WMH×MCI** | **ES CI Upper WMH×MCI** | **p WMH×AD** | **ES WMH×AD** | **ES CI Lower WMH×AD** | **ES CI Upper WMH×AD** | **FDR p WMH×MCI** | **FDR p WMH×AD** | **FDR p WMHxMCI (APOEe4 + edu adj)** | **FDR p WMHxAD (APOEe4 + edu adj)** |
| --- | --- | --- | --- | --- | --- | --- | --- | --- | --- | --- | --- | --- | --- |
| pvscount | unadjusted | 0.76 | 0.98 | 0.88 | 1.10 | 0.86 | 1.02 | 0.82 | 1.27 | 0.86 | 0.88 | 0.76 | 0.88 |
| icvnormlog | unadjusted | 0.53 | -0.07 | -0.43 | 0.29 | 0.69 | -0.09 | -0.39 | 0.20 | 0.56 | 0.84 | 0.54 | 0.96 |
| pvscountwm | unadjusted | 0.59 | 0.97 | 0.87 | 1.08 | 0.88 | 1.02 | 0.81 | 1.27 | 0.86 | 0.88 | 0.74 | 0.88 |
| icvnormwmlog | unadjusted | 0.54 | -0.06 | -0.42 | 0.30 | 0.75 | -0.09 | -0.39 | 0.20 | 0.56 | 0.84 | 0.54 | 0.96 |
| pvscountgm | unadjusted | 0.10 | 1.10 | 0.98 | 1.23 | 0.40 | 1.10 | 0.88 | 1.39 | 0.26 | 0.88 | 0.27 | 0.88 |
| icvnormgmlog | unadjusted | 0.30 | -0.04 | -0.39 | 0.31 | 0.84 | 0.15 | -0.13 | 0.44 | 0.56 | 0.84 | 0.54 | 0.96 |
| pvscountwmh | unadjusted | 0.04 | 1.29 | 1.01 | 1.66 | 0.61 | 0.89 | 0.58 | 1.37 | 0.19 | 0.88 | 0.22 | 0.88 |
| icvnormwmhlog | unadjusted | 0.05 | 0.07 | -0.23 | 0.38 | 0.64 | -0.25 | -0.49 | 2.85e-3 | 0.27 | 0.84 | 0.26 | 0.96 |
| pvscountnonwmh | unadjusted | 0.76 | 0.98 | 0.88 | 1.10 | 0.87 | 1.02 | 0.81 | 1.27 | 0.86 | 0.88 | 0.76 | 0.88 |
| icvnormnonwmhlog | unadjusted | 0.53 | -0.08 | -0.44 | 0.28 | 0.67 | -0.09 | -0.39 | 0.20 | 0.56 | 0.84 | 0.54 | 0.96 |
| pvscount | adjusted | 0.86 | 0.99 | 0.89 | 1.11 | 0.78 | 1.03 | 0.83 | 1.29 | 0.86 | 0.88 | 0.74 | 0.88 |
| icvnormlog | adjusted | 0.56 | -0.08 | -0.44 | 0.28 | 0.67 | -0.09 | -0.38 | 0.21 | 0.56 | 0.84 | 0.54 | 0.96 |
| pvscountwm | adjusted | 0.69 | 0.98 | 0.88 | 1.09 | 0.80 | 1.03 | 0.82 | 1.29 | 0.86 | 0.88 | 0.74 | 0.88 |
| icvnormwmlog | adjusted | 0.56 | -0.06 | -0.42 | 0.30 | 0.75 | -0.09 | -0.38 | 0.21 | 0.56 | 0.84 | 0.54 | 0.96 |
| pvscountgm | adjusted | 0.06 | 1.12 | 1.00 | 1.25 | 0.34 | 1.12 | 0.89 | 1.41 | 0.19 | 0.88 | 0.27 | 0.88 |
| icvnormgmlog | adjusted | 0.24 | -0.06 | -0.40 | 0.28 | 0.74 | 0.17 | -0.11 | 0.44 | 0.56 | 0.84 | 0.54 | 0.96 |
| pvscountwmh | adjusted | 0.02 | 1.34 | 1.05 | 1.72 | 0.55 | 0.88 | 0.57 | 1.34 | 0.19 | 0.88 | 0.22 | 0.88 |
| icvnormwmhlog | adjusted | 0.05 | 0.07 | -0.23 | 0.37 | 0.66 | -0.24 | -0.49 | 3.78e-3 | 0.27 | 0.84 | 0.18 | 0.96 |
| pvscountnonwmh | adjusted | 0.85 | 0.99 | 0.89 | 1.11 | 0.79 | 1.03 | 0.82 | 1.29 | 0.86 | 0.88 | 0.74 | 0.88 |
| icvnormnonwmhlog | adjusted | 0.56 | -0.08 | -0.44 | 0.28 | 0.65 | -0.09 | -0.38 | 0.21 | 0.56 | 0.84 | 0.54 | 0.96 |

**S3C1: amyloid main effects**

| **Lesion Type** | **Adjustment** | **p WMH** | **ES WMH** | **ES CI Lower WMH** | **ES CI Upper WMH** | **p AB** | **ES AB** | **ES CI Lower AB** | **ES CI Upper AB** | **FDR p WMH** | **FDR p AB** | **FDR p WMH (APOEe4 + edu adj)** |
| --- | --- | --- | --- | --- | --- | --- | --- | --- | --- | --- | --- | --- |
| pvscount | unadjusted | 2.53e-4 | 1.13 | 1.06 | 1.21 | 0.29 | 0.71 | 0.37 | 1.34 | 8.38e-4 | 0.78 | 0.00 |
| icvnormlog | unadjusted | 5.83e-4 | 0.14 | 0.06 | 0.22 | 0.63 | -0.07 | -0.37 | 0.22 | 1.46e-3 | 0.80 | 0.00 |
| pvscountwm | unadjusted | 2.62e-3 | 1.11 | 1.04 | 1.19 | 0.23 | 0.67 | 0.35 | 1.28 | 5.25e-3 | 0.78 | 0.01 |
| icvnormwmlog | unadjusted | 0.03 | 0.09 | 0.01 | 0.17 | 0.51 | -0.10 | -0.40 | 0.20 | 0.04 | 0.78 | 0.04 |
| pvscountgm | unadjusted | 5.16e-14 | 1.31 | 1.22 | 1.40 | 0.75 | 1.12 | 0.57 | 2.19 | 5.16e-13 | 0.88 | 0.00 |
| icvnormgmlog | unadjusted | 4.65e-10 | 0.25 | 0.17 | 0.33 | 0.82 | 0.03 | -0.26 | 0.32 | 2.33e-9 | 0.89 | 0.00 |
| pvscountwmh | unadjusted | 3.56e-16 | 1.78 | 1.55 | 2.04 | 0.32 | 0.50 | 0.13 | 1.94 | 7.12e-15 | 0.78 | 0.00 |
| icvnormwmhlog | unadjusted | 0.02 | 0.10 | 0.02 | 0.18 | 0.50 | 0.10 | -0.19 | 0.39 | 0.03 | 0.78 | 0.03 |
| pvscountnonwmh | unadjusted | 2.93e-4 | 1.13 | 1.06 | 1.21 | 0.29 | 0.71 | 0.37 | 1.35 | 8.38e-4 | 0.78 | 0.00 |
| icvnormnonwmhlog | unadjusted | 7.99e-4 | 0.14 | 0.06 | 0.22 | 0.64 | -0.07 | -0.37 | 0.23 | 1.78e-3 | 0.80 | 0.00 |
| pvscount | adjusted | 0.09 | 1.06 | 0.99 | 1.15 | 0.33 | 0.73 | 0.38 | 1.38 | 0.12 | 0.78 | 0.12 |
| icvnormlog | adjusted | 0.10 | 0.07 | -0.01 | 0.16 | 0.54 | -0.09 | -0.39 | 0.20 | 0.12 | 0.78 | 0.12 |
| pvscountwm | adjusted | 0.21 | 1.05 | 0.97 | 1.13 | 0.26 | 0.69 | 0.36 | 1.31 | 0.22 | 0.78 | 0.24 |
| icvnormwmlog | adjusted | 0.34 | 0.04 | -0.05 | 0.13 | 0.45 | -0.12 | -0.41 | 0.18 | 0.34 | 0.78 | 0.35 |
| pvscountgm | adjusted | 9.82e-5 | 1.16 | 1.08 | 1.25 | 0.84 | 1.07 | 0.55 | 2.09 | 3.93e-4 | 0.89 | 0.00 |
| icvnormgmlog | adjusted | 6.67e-3 | 0.12 | 0.03 | 0.20 | 0.99 | 2.00e-3 | -0.28 | 0.28 | 0.01 | 0.99 | 0.01 |
| pvscountwmh | adjusted | 1.37e-12 | 1.69 | 1.46 | 1.96 | 0.36 | 0.53 | 0.14 | 2.04 | 9.16e-12 | 0.78 | 0.00 |
| icvnormwmhlog | adjusted | 0.04 | 0.09 | 2.40e-3 | 0.18 | 0.53 | 0.10 | -0.20 | 0.39 | 0.06 | 0.78 | 0.06 |
| pvscountnonwmh | adjusted | 0.10 | 1.06 | 0.99 | 1.14 | 0.33 | 0.73 | 0.38 | 1.39 | 0.12 | 0.78 | 0.12 |
| icvnormnonwmhlog | adjusted | 0.11 | 0.07 | -0.02 | 0.16 | 0.55 | -0.09 | -0.39 | 0.21 | 0.13 | 0.78 | 0.13 |

**S3C2: amyloid interaction effects**

| **Lesion Type** | **Adjustment** | **p WMH×AB** | **ES WMH×AB** | **ES CI Lower WMH×AB** | **ES CI Upper WMH×AB** | **FDR p WMH×AB** | **FDR p WMHxAB (APOEe4 + edu adj)** |
| --- | --- | --- | --- | --- | --- | --- | --- |
| pvscount | unadjusted | 0.18 | 0.92 | 0.83 | 1.04 | 0.57 | 0.52 |
| icvnormlog | unadjusted | 0.39 | -0.13 | -0.42 | 0.16 | 0.57 | 0.56 |
| pvscountwm | unadjusted | 0.13 | 0.92 | 0.82 | 1.03 | 0.57 | 0.52 |
| icvnormwmlog | unadjusted | 0.31 | -0.15 | -0.45 | 0.14 | 0.57 | 0.56 |
| pvscountgm | unadjusted | 0.82 | 1.01 | 0.90 | 1.14 | 0.97 | 0.98 |
| icvnormgmlog | unadjusted | 0.93 | 0.01 | -0.27 | 0.30 | 0.97 | 0.98 |
| pvscountwmh | unadjusted | 0.37 | 0.89 | 0.70 | 1.14 | 0.57 | 0.56 |
| icvnormwmhlog | unadjusted | 0.92 | 0.01 | -0.27 | 0.30 | 0.97 | 0.98 |
| pvscountnonwmh | unadjusted | 0.18 | 0.93 | 0.83 | 1.04 | 0.57 | 0.52 |
| icvnormnonwmhlog | unadjusted | 0.39 | -0.13 | -0.42 | 0.17 | 0.57 | 0.56 |
| pvscount | adjusted | 0.21 | 0.93 | 0.83 | 1.04 | 0.57 | 0.52 |
| icvnormlog | adjusted | 0.34 | -0.14 | -0.43 | 0.15 | 0.57 | 0.52 |
| pvscountwm | adjusted | 0.15 | 0.92 | 0.82 | 1.03 | 0.57 | 0.52 |
| icvnormwmlog | adjusted | 0.28 | -0.16 | -0.46 | 0.13 | 0.57 | 0.52 |
| pvscountgm | adjusted | 0.88 | 1.01 | 0.90 | 1.14 | 0.97 | 0.98 |
| icvnormgmlog | adjusted | 0.97 | -5.68e-3 | -0.28 | 0.27 | 0.97 | 0.98 |
| pvscountwmh | adjusted | 0.40 | 0.90 | 0.70 | 1.15 | 0.57 | 0.56 |
| icvnormwmhlog | adjusted | 0.95 | 9.89e-3 | -0.28 | 0.30 | 0.97 | 1.00 |
| pvscountnonwmh | adjusted | 0.22 | 0.93 | 0.83 | 1.04 | 0.57 | 0.52 |
| icvnormnonwmhlog | adjusted | 0.35 | -0.14 | -0.43 | 0.15 | 0.57 | 0.52 |

**S3D1 biomarker group main effects**

| **Lesion Type** | **Adjustment** | **p WMH** | **ES WMH** | **ES CI Lower WMH** | **ES CI Upper WMH** | **p CU AB+** | **ES CU AB+** | **ES CI Lower CU AB+** | **ES CI Upper CU AB+** | **p MCI AB+** | **ES MCI AB+** | **ES CI Lower MCI AB+** | **ES CI Upper MCI AB+** | **p AD AB+** | **ES AD AB+** | **ES CI Lower AD AB+** | **ES CI Upper AD AB+** | **FDR p WMH** | **FDR p CU AB+** | **FDR p MCI AB+** | **FDR p AD AB+** | **FDR p CUAB+ (APOEe4 + edu adj)** | **FDR p MCIAB+(APOEe4 + edu adj)** | **FDR p ADAB+ (APOEe4 + edu adj)** |
| --- | --- | --- | --- | --- | --- | --- | --- | --- | --- | --- | --- | --- | --- | --- | --- | --- | --- | --- | --- | --- | --- | --- | --- | --- |
| pvscount | unadjusted | 7.29e-3 | 1.12 | 1.03 | 1.21 | 0.94 | 0.97 | 0.41 | 2.28 | 0.39 | 0.63 | 0.23 | 1.78 | 0.44 | 0.56 | 0.13 | 2.44 | 0.02 | 0.95 | 0.62 | 0.62 | 1.00 | 0.57 | 0.62 |
| icvnormlog | unadjusted | 4.66e-3 | 0.14 | 0.04 | 0.24 | 0.78 | 0.05 | -0.28 | 0.38 | 0.64 | -0.10 | -0.49 | 0.30 | 0.30 | -0.24 | -0.70 | 0.21 | 0.02 | 0.95 | 0.71 | 0.62 | 1.00 | 0.71 | 0.62 |
| pvscountwm | unadjusted | 0.03 | 1.10 | 1.01 | 1.19 | 0.92 | 0.96 | 0.40 | 2.26 | 0.23 | 0.53 | 0.19 | 1.50 | 0.36 | 0.50 | 0.11 | 2.19 | 0.05 | 0.95 | 0.62 | 0.62 | 1.00 | 0.57 | 0.62 |
| icvnormwmlog | unadjusted | 0.08 | 0.09 | -9.58e-3 | 0.19 | 0.90 | 0.02 | -0.31 | 0.35 | 0.50 | -0.14 | -0.54 | 0.26 | 0.35 | -0.22 | -0.68 | 0.24 | 0.11 | 0.95 | 0.62 | 0.62 | 1.00 | 0.65 | 0.62 |
| pvscountgm | unadjusted | 5.29e-7 | 1.24 | 1.14 | 1.35 | 0.49 | 1.38 | 0.56 | 3.43 | 0.17 | 2.10 | 0.73 | 6.09 | 0.69 | 1.36 | 0.30 | 6.17 | 5.29e-6 | 0.95 | 0.62 | 0.86 | 1.00 | 0.57 | 0.86 |
| icvnormgmlog | unadjusted | 6.17e-6 | 0.22 | 0.13 | 0.32 | 0.72 | 0.06 | -0.26 | 0.38 | 0.26 | 0.22 | -0.16 | 0.61 | 0.88 | -0.03 | -0.48 | 0.41 | 3.08e-5 | 0.95 | 0.62 | 0.91 | 1.00 | 0.57 | 0.91 |
| pvscountwmh | unadjusted | 8.28e-9 | 1.61 | 1.37 | 1.89 | 0.65 | 1.59 | 0.22 | 11.65 | 0.92 | 1.11 | 0.14 | 8.78 | 0.13 | 0.16 | 0.02 | 1.71 | 1.66e-7 | 0.95 | 0.97 | 0.62 | 1.00 | 0.92 | 0.62 |
| icvnormwmhlog | unadjusted | 3.45e-3 | 0.12 | 0.04 | 0.20 | 0.95 | 8.57e-3 | -0.26 | 0.28 | 5.14e-3 | 0.46 | 0.14 | 0.78 | 0.91 | -0.02 | -0.39 | 0.35 | 0.01 | 0.95 | 0.06 | 0.91 | 1.00 | 0.10 | 0.91 |
| pvscountnonwmh | unadjusted | 8.03e-3 | 1.12 | 1.03 | 1.21 | 0.95 | 0.97 | 0.41 | 2.30 | 0.39 | 0.64 | 0.23 | 1.79 | 0.42 | 0.55 | 0.12 | 2.40 | 0.02 | 0.95 | 0.62 | 0.62 | 1.00 | 0.57 | 0.62 |
| icvnormnonwmhlog | unadjusted | 5.79e-3 | 0.14 | 0.04 | 0.24 | 0.76 | 0.05 | -0.28 | 0.38 | 0.63 | -0.10 | -0.49 | 0.30 | 0.29 | -0.24 | -0.70 | 0.21 | 0.02 | 0.95 | 0.71 | 0.62 | 1.00 | 0.71 | 0.62 |
| pvscount | adjusted | 0.21 | 1.06 | 0.97 | 1.16 | 0.92 | 1.05 | 0.44 | 2.47 | 0.34 | 0.61 | 0.21 | 1.71 | 0.39 | 0.52 | 0.12 | 2.30 | 0.24 | 0.95 | 0.62 | 0.62 | 1.00 | 0.57 | 0.62 |
| icvnormlog | adjusted | 0.13 | 0.08 | -0.02 | 0.19 | 0.75 | 0.05 | -0.27 | 0.38 | 0.48 | -0.14 | -0.54 | 0.25 | 0.24 | -0.27 | -0.72 | 0.18 | 0.17 | 0.95 | 0.62 | 0.62 | 1.00 | 0.57 | 0.62 |
| pvscountwm | adjusted | 0.35 | 1.04 | 0.95 | 1.14 | 0.95 | 1.03 | 0.43 | 2.43 | 0.21 | 0.51 | 0.18 | 1.45 | 0.31 | 0.47 | 0.11 | 2.05 | 0.37 | 0.95 | 0.62 | 0.62 | 1.00 | 0.57 | 0.62 |
| icvnormwmlog | adjusted | 0.37 | 0.05 | -0.06 | 0.15 | 0.88 | 0.03 | -0.31 | 0.36 | 0.40 | -0.17 | -0.57 | 0.23 | 0.31 | -0.24 | -0.70 | 0.22 | 0.37 | 0.95 | 0.62 | 0.62 | 1.00 | 0.57 | 0.62 |
| pvscountgm | adjusted | 0.03 | 1.11 | 1.01 | 1.21 | 0.47 | 1.40 | 0.57 | 3.45 | 0.22 | 1.94 | 0.67 | 5.65 | 0.77 | 1.26 | 0.28 | 5.72 | 0.05 | 0.95 | 0.62 | 0.90 | 1.00 | 0.57 | 0.86 |
| icvnormgmlog | adjusted | 0.06 | 0.10 | -5.63e-3 | 0.20 | 0.63 | 0.08 | -0.24 | 0.39 | 0.45 | 0.15 | -0.23 | 0.52 | 0.69 | -0.09 | -0.52 | 0.35 | 0.10 | 0.95 | 0.62 | 0.86 | 1.00 | 0.65 | 0.86 |
| pvscountwmh | adjusted | 8.74e-7 | 1.53 | 1.29 | 1.82 | 0.63 | 1.63 | 0.22 | 11.84 | 0.98 | 1.03 | 0.13 | 8.01 | 0.09 | 0.13 | 0.01 | 1.34 | 5.83e-6 | 0.95 | 0.98 | 0.62 | 1.00 | 0.92 | 0.62 |
| icvnormwmhlog | adjusted | 0.01 | 0.11 | 0.02 | 0.19 | 0.94 | 0.01 | -0.26 | 0.28 | 5.68e-3 | 0.46 | 0.13 | 0.78 | 0.89 | -0.03 | -0.40 | 0.35 | 0.03 | 0.95 | 0.06 | 0.91 | 1.00 | 0.13 | 0.91 |
| pvscountnonwmh | adjusted | 0.22 | 1.06 | 0.97 | 1.15 | 0.91 | 1.05 | 0.45 | 2.49 | 0.35 | 0.61 | 0.22 | 1.71 | 0.38 | 0.51 | 0.12 | 2.26 | 0.25 | 0.95 | 0.62 | 0.62 | 1.00 | 0.57 | 0.62 |
| icvnormnonwmhlog | adjusted | 0.15 | 0.08 | -0.03 | 0.18 | 0.73 | 0.06 | -0.27 | 0.39 | 0.47 | -0.14 | -0.54 | 0.25 | 0.24 | -0.27 | -0.73 | 0.18 | 0.18 | 0.95 | 0.62 | 0.62 | 1.00 | 0.57 | 0.62 |

**S3D2 biomarker group interaction effects**

| **Lesion Type** | **Adjustment** | **p WMH×CU AB+** | **ES WMH×CU AB+** | **ES CI Lower WMH×CU AB+** | **ES CI Upper WMH×CU AB+** | **p WMH×MCI AB+** | **ES WMH×MCI AB+** | **ES CI Lower WMH×MCI AB+** | **ES CI Upper WMH×MCI AB+** | **p WMH×AD AB+** | **ES WMH×AD AB+** | **ES CI Lower WMH×AD AB+** | **ES CI Upper WMH×AD AB+** | **FDR p WMH×CU AB+** | **FDR p WMH×MCI AB+** | **FDR p WMH×AD AB+** | **FDR p WMH×CUAB+ (APOEe4+edu adj)** | **FDR p WMH× MCIAB+(APOEe4+edu adj)** | **FDR p WMH×ADAB+(APOEe4+edu adj)** |
| --- | --- | --- | --- | --- | --- | --- | --- | --- | --- | --- | --- | --- | --- | --- | --- | --- | --- | --- | --- |
| pvscount | unadjusted | 0.79 | 0.98 | 0.85 | 1.14 | 0.20 | 0.89 | 0.74 | 1.07 | 0.40 | 0.89 | 0.67 | 1.18 | 0.99 | 0.56 | 0.58 | 0.99 | 0.20 | 0.40 |
| icvnormlog | unadjusted | 0.98 | -4.29e-3 | -0.33 | 0.32 | 0.41 | -0.16 | -0.55 | 0.22 | 0.28 | -0.25 | -0.70 | 0.20 | 0.99 | 0.56 | 0.58 | 0.99 | 0.41 | 0.28 |
| pvscountwm | unadjusted | 0.79 | 0.98 | 0.85 | 1.13 | 0.11 | 0.86 | 0.71 | 1.03 | 0.32 | 0.87 | 0.65 | 1.15 | 0.99 | 0.56 | 0.58 | 0.99 | 0.11 | 0.32 |
| icvnormwmlog | unadjusted | 0.88 | -0.03 | -0.35 | 0.30 | 0.31 | -0.20 | -0.59 | 0.19 | 0.32 | -0.23 | -0.68 | 0.22 | 0.99 | 0.56 | 0.58 | 0.99 | 0.31 | 0.32 |
| pvscountgm | unadjusted | 0.56 | 1.05 | 0.90 | 1.22 | 0.34 | 1.10 | 0.91 | 1.33 | 0.79 | 1.04 | 0.78 | 1.39 | 0.99 | 0.56 | 0.85 | 0.99 | 0.34 | 0.79 |
| icvnormgmlog | unadjusted | 0.81 | 0.04 | -0.28 | 0.36 | 0.38 | 0.17 | -0.21 | 0.55 | 0.75 | -0.07 | -0.51 | 0.37 | 0.99 | 0.56 | 0.85 | 0.99 | 0.38 | 0.75 |
| pvscountwmh | unadjusted | 0.60 | 1.10 | 0.77 | 1.58 | 0.87 | 1.03 | 0.70 | 1.51 | 0.10 | 0.69 | 0.44 | 1.08 | 0.99 | 0.91 | 0.58 | 0.99 | 0.87 | 0.10 |
| icvnormwmhlog | unadjusted | 0.96 | 7.25e-3 | -0.26 | 0.27 | 0.48 | -0.11 | -0.43 | 0.20 | 0.85 | -0.04 | -0.40 | 0.33 | 0.99 | 0.57 | 0.85 | 0.99 | 0.48 | 0.85 |
| pvscountnonwmh | unadjusted | 0.80 | 0.98 | 0.85 | 1.14 | 0.20 | 0.89 | 0.74 | 1.07 | 0.39 | 0.88 | 0.67 | 1.17 | 0.99 | 0.56 | 0.58 | 0.99 | 0.20 | 0.39 |
| icvnormnonwmhlog | unadjusted | 0.99 | -1.22e-3 | -0.33 | 0.33 | 0.40 | -0.17 | -0.55 | 0.22 | 0.28 | -0.25 | -0.70 | 0.20 | 0.99 | 0.56 | 0.58 | 0.99 | 0.40 | 0.28 |
| pvscount | adjusted | 0.95 | 1.00 | 0.86 | 1.15 | 0.19 | 0.88 | 0.73 | 1.06 | 0.40 | 0.89 | 0.67 | 1.17 | 0.99 | 0.56 | 0.58 | 0.99 | 0.15 | 0.43 |
| icvnormlog | adjusted | 0.97 | 5.92e-3 | -0.32 | 0.33 | 0.31 | -0.20 | -0.59 | 0.19 | 0.25 | -0.26 | -0.71 | 0.18 | 0.99 | 0.56 | 0.58 | 0.99 | 0.23 | 0.25 |
| pvscountwm | adjusted | 0.93 | 0.99 | 0.86 | 1.15 | 0.10 | 0.86 | 0.71 | 1.03 | 0.31 | 0.86 | 0.65 | 1.15 | 0.99 | 0.56 | 0.58 | 0.99 | 0.08 | 0.35 |
| icvnormwmlog | adjusted | 0.91 | -0.02 | -0.35 | 0.31 | 0.25 | -0.23 | -0.62 | 0.16 | 0.30 | -0.24 | -0.69 | 0.21 | 0.99 | 0.56 | 0.58 | 0.99 | 0.20 | 0.30 |
| pvscountgm | adjusted | 0.51 | 1.05 | 0.90 | 1.23 | 0.42 | 1.08 | 0.89 | 1.31 | 0.76 | 1.05 | 0.78 | 1.40 | 0.99 | 0.56 | 0.85 | 0.99 | 0.54 | 0.73 |
| icvnormgmlog | adjusted | 0.66 | 0.07 | -0.24 | 0.38 | 0.57 | 0.11 | -0.26 | 0.48 | 0.63 | -0.10 | -0.53 | 0.32 | 0.99 | 0.63 | 0.84 | 0.99 | 0.68 | 0.61 |
| pvscountwmh | adjusted | 0.59 | 1.10 | 0.77 | 1.58 | 0.91 | 1.02 | 0.70 | 1.49 | 0.07 | 0.66 | 0.42 | 1.03 | 0.99 | 0.91 | 0.58 | 0.99 | 0.91 | 0.13 |
| icvnormwmhlog | adjusted | 0.93 | 0.01 | -0.26 | 0.28 | 0.47 | -0.12 | -0.43 | 0.20 | 0.83 | -0.04 | -0.41 | 0.33 | 0.99 | 0.57 | 0.85 | 0.99 | 0.31 | 0.84 |
| pvscountnonwmh | adjusted | 0.96 | 1.00 | 0.86 | 1.15 | 0.19 | 0.88 | 0.73 | 1.06 | 0.39 | 0.88 | 0.66 | 1.17 | 0.99 | 0.56 | 0.58 | 0.99 | 0.15 | 0.42 |
| icvnormnonwmhlog | adjusted | 0.96 | 8.88e-3 | -0.32 | 0.33 | 0.30 | -0.20 | -0.59 | 0.18 | 0.25 | -0.26 | -0.71 | 0.18 | 0.99 | 0.56 | 0.58 | 0.99 | 0.23 | 0.24 |

**S4A: Proximity analysis: diagnostic group**

| **Measure** | **Measure Label** | **Comparison** | **ES Unadj** | **CI unadj** | **p Unadj** | **ES Adj** | **CI Adj** | **p Adj** | **FDR p Unadj** | **FDR p Adj** | **FDR p (WML vol adj)** | **FDR p (APOEe4+**  **edu adj)** |
| --- | --- | --- | --- | --- | --- | --- | --- | --- | --- | --- | --- | --- |
| pvscountwmh | Within WMH | CU vs MCI | 1.31 | 0.98,1.65 | 0.03 | 1.27 | 0.93,1.55 | 0.16 | 0.10 | 0.24 | 0.64 | 0.25 |
| pvscountwmh | Within WMH | CU vs AD | 1.58 | 1.02,2.21 | 0.02 | 1.53 | 1,2.2 | 0.05 | 0.10 | 0.14 | 0.64 | 0.09 |
| pvscountwmh | Within WMH | MCI vs AD | 1.20 | 0.79,1.75 | 0.34 | 1.17 | 0.8,1.74 | 0.40 | 0.41 | 0.40 | 0.64 | 0.54 |
| close_pvs_count | Near WMH | CU vs MCI | 1.24 | 1.03,1.51 | 0.02 | 1.19 | 1,1.46 | 0.05 | 0.03 | 0.09 | 0.96 | 0.06 |
| close_pvs_count | Near WMH | CU vs AD | 1.35 | 1.06,1.85 | 0.03 | 1.22 | 0.98,1.72 | 0.06 | 0.03 | 0.09 | 0.96 | 0.06 |
| close_pvs_count | Near WMH | MCI vs AD | 1.09 | 0.83,1.51 | 0.58 | 1.04 | 0.80,1.45 | 0.60 | 0.46 | 0.60 | 0.96 | 0.65 |
| far_pvs_count | Far from WMH | CU vs MCI | 1.10 | 0.96,1.26 | 0.16 | 1.05 | 0.93,1.22 | 0.36 | 0.45 | 0.88 | 0.59 | 0.72 |
| far_pvs_count | Far from WMH | CU vs AD | 1.16 | 0.90,1.34 | 0.12 | 1.07 | 0.83,1.24 | 0.88 | 0.52 | 0.88 | 0.59 | 0.96 |
| far_pvs_count | Far from WMH | MCI vs AD | 1.06 | 0.8,1.24 | 0.60 | 1.01 | 0.76,1.18 | 0.65 | 0.97 | 0.88 | 0.83 | 0.72 |

**S4B: Proximity analysis: amyloid**

| **Measure** | **Measure Label** | **Comparison** | **ES Unadj** | **CI Unadj** | **p Unadj** | **ES Adj** | **CI Adj** | **p Adj** | **FDR p Unadj** | **FDR p Adj** | **FDR p (WML vol adj)** | **FDR p**  **(APOEe4 +**  **edu adj vol)** |
| --- | --- | --- | --- | --- | --- | --- | --- | --- | --- | --- | --- | --- |
| pvscountwmh | Within WMH | AB- vs AB+ | 1.03 | 0.80,1.32 | 0.82 | 1.05 | 0.82,1.33 | 0.71 | 0.82 | 0.71 | 0.58 | 0.86 |
| close_pvs_count | Near WMH | AB- vs AB+ | 1.26 | 1.06,1.51 | 0.01 | 1.23 | 1.03,1.46 | 0.02 | 0.01 | 0.02 | 0.19 | 0.03 |
| far_pvs_count | Far from WMH | AB- vs AB+ | 1.08 | 0.95,1.24 | 0.23 | 1.06 | 0.93,1.21 | 0.33 | 0.19 | 0.33 | 0.29 | 0.44 |

**S4C: Proximity analysis: biomarker**

| **Measure** | **Measure Label** | **Comparison** | **ES Unadj** | **CI Unadj** | **p Unadj** | **ES Adj** | **CI Adj** | **p Adj** | **FDR p Unadj** | **FDR p Adj** | **FDR p (WMH vol adj)** | **FDR p (APOEe4+edu adj)** |
| --- | --- | --- | --- | --- | --- | --- | --- | --- | --- | --- | --- | --- |
| pvscountwmh | Within WML | CU_AB- vs CU_AB+ | 1.10 | 0.75,1.61 | 0.61 | 1.10 | 0.761,1.57 | 0.63 | 0.73 | 0.94 | 0.86 | 0.96 |
| pvscountwmh | Within WML | CU_AB- vs MCI_AB+ | 1.16 | 0.81,1.67 | 0.41 | 1.03 | 0.729,1.46 | 0.86 | 0.62 | 0.96 | 0.86 | 0.96 |
| pvscountwmh | Within WML | CU_AB- vs AD_AB+ | 1.44 | 0.89,2.26 | 0.12 | 1.43 | 0.893,2.25 | 0.14 | 0.62 | 0.82 | 0.86 | 0.96 |
| pvscountwmh | Within WML | CU_AB+ vs MCI_AB+ | 1.06 | 0.71,1.56 | 0.78 | 0.99 | 0.669,1.47 | 0.96 | 0.79 | 0.96 | 0.86 | 0.96 |
| pvscountwmh | Within WML | CU_AB+ vs AD_AB+ | 1.30 | 0.79,2.1 | 0.28 | 1.24 | 0.749,2.03 | 0.41 | 0.62 | 0.82 | 0.86 | 0.96 |
| pvscountwmh | Within WML | MCI_AB+ vs AD_AB+ | 1.23 | 0.77,1.92 | 0.36 | 1.22 | 0.771,1.9 | 0.41 | 0.62 | 0.82 | 0.86 | 0.96 |
| close_pvs_count | Near WML | CU_AB- vs CU_AB+ | 1.34 | 1.02,1.76 | 0.03 | 1.29 | 0.985,1.66 | 0.06 | 0.10 | 0.19 | 0.51 | 0.40 |
| close_pvs_count | Near WML | CU_AB- vs MCI_AB+ | 1.59 | 1.23,2.11 | 6.94e-4 | 1.45 | 1.13,1.91 | 0.00 | 0.00 | 0.03 | 0.45 | 0.27 |
| close_pvs_count | Near WML | CU_AB- vs AD_AB+ | 1.39 | 0.99,1.94 | 0.05 | 1.24 | 0.896,1.72 | 0.19 | 0.11 | 0.39 | 0.77 | 0.56 |
| close_pvs_count | Near WML | CU_AB+ vs MCI_AB+ | 1.19 | 0.89,1.6 | 0.24 | 1.13 | 0.854,1.53 | 0.37 | 0.34 | 0.45 | 0.59 | 0.59 |
| close_pvs_count | Near WML | CU_AB+ vs AD_AB+ | 1.04 | 0.74,1.44 | 0.82 | 0.98 | 0.7,1.36 | 0.89 | 0.84 | 0.89 | 0.51 | 0.56 |
| close_pvs_count | Near WML | MCI_AB+ vs AD_AB+ | 0.87 | 0.61,1.21 | 0.43 | 0.85 | 0.606,1.17 | 0.31 | 0.47 | 0.45 | 0.45 | 0.30 |
| far_pvs_count | Far from WML | CU_AB- vs CU_AB+ | 1.08 | 0.90,1.31 | 0.40 | 1.06 | 0.889,1.28 | 0.49 | 0.53 | 0.73 | 0.83 | 0.56 |
| far_pvs_count | Far from WML | CU_AB- vs MCI_AB+ | 1.22 | 1.03,1.49 | 0.03 | 1.17 | 0.982,1.42 | 0.08 | 0.15 | 0.47 | 0.46 | 0.38 |
| far_pvs_count | Far from WML | CU_AB- vs AD_AB+ | 1.09 | 0.87 | 0.45 | 0.98 | 0.78 | 0.88 | 0.54 | 0.88 | 0.97 | 0.66 |
| far_pvs_count | Far from WML | CU_AB+ vs MCI_AB+ | 1.13 | 0.91 | 0.26 | 1.10 | 0.89 | 0.37 | 0.54 | 0.73 | 0.73 | 0.56 |
| far_pvs_count | Far from WML | CU_AB+ vs AD_AB+ | 1.01 | 0.78 | 0.94 | 0.95 | 0.73 | 0.68 | 0.94 | 0.81 | 0.83 | 0.56 |
| far_pvs_count | Far from WML | MCI_AB+ vs AD_AB+ | 0.89 | 0.69 | 0.39 | 0.86 | 0.66 | 0.28 | 0.54 | 0.73 | 0.73 | 0.56 |

**S5A: Granular distance analysis: diagnostic group**

| **Measure** | **Measure Label** | **Comparison** | **ES Unadj** | **CI Unadj** | **p Unadj** | **ES adj** | **CI Adj** | **P adj** | **FDR p unadj** | **FDR p adj** | **FDR p**  **(WMH vol adj)** | **FDR p**  **(APOEe4 + edu adj** |
| --- | --- | --- | --- | --- | --- | --- | --- | --- | --- | --- | --- | --- |
| pvscountwmh | 0.00 | CU vs MCI | 1.31 | 0.985,1.65 | 0.03 | 1.20 | 0.931,1.55 | 0.16 | 0.10 | 0.24 | 0.64 | 0.25 |
| pvscountwmh | 0.00 | CU vs AD | 1.58 | 1.02,2.21 | 0.02 | 1.49 | 1,2.2 | 0.05 | 0.10 | 0.14 | 0.64 | 0.09 |
| pvscountwmh | 0.00 | MCI vs AD | 1.20 | 0.795,1.75 | 0.34 | 1.18 | 0.8,1.74 | 0.40 | 0.41 | 0.40 | 0.64 | 0.54 |
| count5 | <=5mm | CU vs MCI | 1.24 | 1.03,1.51 | 0.02 | 1.21 | 1,1.46 | 0.05 | 0.03 | 0.09 | 0.96 | 0.06 |
| count5 | <=5mm | CU vs AD | 1.35 | 1.06,1.85 | 0.03 | 1.30 | 0.989,1.72 | 0.06 | 0.03 | 0.09 | 0.96 | 0.06 |
| count5 | <=5mm | MCI vs AD | 1.09 | 0.832,1.51 | 0.57 | 1.08 | 0.807,1.46 | 0.59 | 0.45 | 0.59 | 0.96 | 0.64 |
| count10 | 5-10mm | CU vs MCI | 1.21 | 1.05,1.45 | 0.01 | 1.19 | 1.02,1.39 | 0.03 | 0.01 | 0.04 | 0.50 | 0.08 |
| count10 | 5-10mm | CU vs AD | 1.46 | 1.14,1.83 | 7.13e-4 | 1.30 | 1.03,1.64 | 0.03 | 0.01 | 0.04 | 0.50 | 0.08 |
| count10 | 5-10mm | MCI vs AD | 1.21 | 0.909,1.51 | 0.12 | 1.11 | 0.869,1.43 | 0.40 | 0.22 | 0.40 | 0.55 | 0.53 |
| count15 | 10-15mm | CU vs MCI | 1.15 | 0.999,1.32 | 0.05 | 1.11 | 0.966,1.27 | 0.14 | 0.08 | 0.43 | 0.72 | 0.61 |
| count15 | 10-15mm | CU vs AD | 1.27 | 1,1.53 | 0.02 | 1.11 | 0.899,1.36 | 0.34 | 0.08 | 0.51 | 0.72 | 0.75 |
| count15 | 10-15mm | MCI vs AD | 1.11 | 0.86,1.35 | 0.35 | 1.01 | 0.805,1.26 | 0.95 | 0.51 | 0.95 | 0.90 | 0.82 |
| count20 | 15-20mm | CU vs MCI | 1.09 | 0.947,1.28 | 0.22 | 1.06 | 0.917,1.24 | 0.41 | 0.31 | 0.64 | 0.48 | 0.75 |
| count20 | 15-20mm | CU vs AD | 1.23 | 0.95,1.49 | 0.05 | 1.10 | 0.876,1.37 | 0.42 | 0.31 | 0.64 | 0.48 | 0.75 |
| count20 | 15-20mm | MCI vs AD | 1.13 | 0.84,1.37 | 0.31 | 1.03 | 0.813,1.32 | 0.78 | 0.53 | 0.78 | 0.48 | 0.98 |
| count25 | 20-25mm | CU vs MCI | 1.03 | 0.871,1.21 | 0.73 | 0.99 | 0.838,1.17 | 0.92 | 0.74 | 0.92 | 0.82 | 0.95 |
| count25 | 20-25mm | CU vs AD | 1.01 | 0.709,1.16 | 0.96 | 0.88 | 0.682,1.13 | 0.31 | 0.67 | 0.47 | 0.82 | 0.60 |
| count25 | 20-25mm | MCI vs AD | 0.98 | 0.679,1.15 | 0.86 | 0.87 | 0.665,1.13 | 0.30 | 0.67 | 0.47 | 0.82 | 0.60 |
| count30 | 25-30mm | CU vs MCI | 0.97 | 0.794,1.2 | 0.76 | 0.92 | 0.751,1.13 | 0.44 | 0.80 | 0.44 | 0.85 | 0.50 |
| count30 | 25-30mm | CU vs AD | 0.76 | 0.49,0.895 | 0.06 | 0.69 | 0.504,0.933 | 0.02 | 0.02 | 0.05 | 0.85 | 0.09 |
| count30 | 25-30mm | MCI vs AD | 0.79 | 0.484,0.956 | 0.14 | 0.74 | 0.523,1.04 | 0.09 | 0.04 | 0.13 | 0.85 | 0.10 |
| count30plus | >30mm | CU vs MCI | 0.95 | 0.721,1.23 | 0.67 | 0.95 | 0.725,1.23 | 0.68 | 0.67 | 0.68 | 0.49 | 0.79 |
| count30plus | >30mm | CU vs AD | 0.57 | 0.266,0.589 | 2.67e-3 | 0.45 | 0.302,0.67 | 0.00 | 0.00 | 0.00 | 0.34 | 0.00 |
| count30plus | >30mm | MCI vs AD | 0.60 | 0.263,0.67 | 0.02 | 0.46 | 0.287,0.734 | 0.00 | 0.00 | 0.00 | 0.34 | 0.00 |

**S5B: Granular distance analysis: amyloid**

| **Measure** | **Measure Label** | **Comparison** | **ES Unadj** | **CI Unadj** | **ES Adj** | **CI Adj** | **FDR p Unadj** | **FDR p Adj** | **FDR p (WMH vol adj)** | **FDR p**  **(APOEe4+edu adj)** |
| --- | --- | --- | --- | --- | --- | --- | --- | --- | --- | --- |
| pvscountwmh | 0.00 | AB- vs AB+ | 1.03 | 0.81, 1.32 | 1.05 | 0.81, 1.32 | 0.82 | 0.70 | 0.58 | 0.86 |
| count5 | 5-10mm | AB- vs AB+ | 1.27 | 1.06, 1.51 | 1.23 | 1.06, 1.51 | 0.01 | 0.02 | 0.19 | 0.03 |
| count10 | 5-10mm | AB- vs AB+ | 1.27 | 1.09, 1.48 | 1.24 | 1.09, 1.48 | 1.89e-3 | 4.38e-3 | 0.03 | 0.02 |
| count15 | 10-15mm | AB- vs AB+ | 1.20 | 1.05, 1.37 | 1.16 | 1.05, 1.37 | 6.93e-3 | 0.03 | 0.05 | 0.06 |
| count20 | 15-20mm | AB- vs AB+ | 1.15 | 1, 1.33 | 1.12 | 1, 1.33 | 0.06 | 0.11 | 0.09 | 0.14 |
| count25 | 20-25mm | AB- vs AB+ | 0.97 | 0.82, 1.13 | 0.97 | 0.82, 1.13 | 0.66 | 0.70 | 0.55 | 0.91 |
| count30 | 25-30mm | AB- vs AB+ | 0.78 | 0.65, 0.96 | 0.82 | 0.65, 0.96 | 0.02 | 0.05 | 0.94 | 0.09 |
| count30plus | >30mm | AB- vs AB+ | 0.66 | 0.51, 0.85 | 0.74 | 0.51, 0.85 | 1.23e-3 | 0.02 | 0.59 | 0.05 |

**S5C: Granular distance analysis: biomarker-informed**

| **Measure** | **Measure Label** | **Comparison** | **ES Unadj** | **CI Unadj** | **p Unadj** | **ES Adj** | **CI Adj** | **P adj** | **FDR p unadj** | **FDR p adj** | **FDR p (WMH vol adj)** | **FDR p (APOEe4+edu adj)** |
| --- | --- | --- | --- | --- | --- | --- | --- | --- | --- | --- | --- | --- |
| pvscountwmh | 0.00 | CU_AB- vs CU_AB+ | 1.10 | 0.758,1.61 | 0.61 | 1.10 | 0.761,1.57 | 0.63 | 0.73 | 0.94 | 0.86 | 0.86 |
| pvscountwmh | 0.00 | CU_AB- vs MCI_AB+ | 1.16 | 0.811,1.67 | 0.41 | 1.03 | 0.729,1.46 | 0.86 | 0.62 | 0.96 | 0.86 | 0.86 |
| pvscountwmh | 0.00 | CU_AB- vs AD_AB+ | 1.44 | 0.897,2.26 | 0.12 | 1.43 | 0.893,2.25 | 0.14 | 0.62 | 0.82 | 0.86 | 0.21 |
| pvscountwmh | 0.00 | CU_AB+ vs MCI_AB+ | 1.06 | 0.713,1.56 | 0.78 | 0.99 | 0.669,1.47 | 0.96 | 0.79 | 0.96 | 0.86 | 0.86 |
| pvscountwmh | 0.00 | CU_AB+ vs AD_AB+ | 1.30 | 0.793,2.1 | 0.28 | 1.24 | 0.749,2.03 | 0.41 | 0.62 | 0.82 | 0.86 | 0.86 |
| pvscountwmh | 0.00 | MCI_AB+ vs AD_AB+ | 1.23 | 0.779,1.92 | 0.36 | 1.22 | 0.771,1.9 | 0.41 | 0.62 | 0.82 | 0.86 | 0.86 |
| count5 | <=5mm | CU_AB- vs CU_AB+ | 1.34 | 1.03,1.76 | 0.03 | 1.29 | 0.987,1.67 | 0.06 | 0.10 | 0.19 | 0.50 | 0.09 |
| count5 | <=5mm | CU_AB- vs MCI_AB+ | 1.59 | 1.23,2.11 | 6.94e-4 | 1.45 | 1.13,1.91 | 0.00 | 0.00 | 0.03 | 0.45 | 0.01 |
| count5 | <=5mm | CU_AB- vs AD_AB+ | 1.40 | 0.996,1.94 | 0.05 | 1.24 | 0.896,1.73 | 0.19 | 0.11 | 0.39 | 0.77 | 0.08 |
| count5 | <=5mm | CU_AB+ vs MCI_AB+ | 1.19 | 0.893,1.6 | 0.25 | 1.13 | 0.853,1.53 | 0.37 | 0.35 | 0.45 | 0.59 | 0.44 |
| count5 | <=5mm | CU_AB+ vs AD_AB+ | 1.04 | 0.744,1.44 | 0.82 | 0.98 | 0.699,1.36 | 0.89 | 0.84 | 0.89 | 0.50 | 1.00 |
| count5 | <=5mm | MCI_AB+ vs AD_AB+ | 0.88 | 0.62,1.21 | 0.43 | 0.85 | 0.606,1.17 | 0.31 | 0.48 | 0.45 | 0.45 | 0.44 |
| count10 | 5-10mm | CU_AB- vs CU_AB+ | 1.36 | 1.09,1.72 | 7.63e-3 | 1.32 | 1.07,1.65 | 0.01 | 0.01 | 0.03 | 0.18 | 0.05 |
| count10 | 5-10mm | CU_AB- vs MCI_AB+ | 1.54 | 1.23,1.94 | 1.79e-4 | 1.42 | 1.16,1.79 | 0.00 | 0.00 | 0.01 | 0.11 | 0.01 |
| count10 | 5-10mm | CU_AB- vs AD_AB+ | 1.52 | 1.14,2.01 | 3.81e-3 | 1.32 | 1.01,1.74 | 0.04 | 0.01 | 0.09 | 0.77 | 0.07 |
| count10 | 5-10mm | CU_AB+ vs MCI_AB+ | 1.13 | 0.88,1.46 | 0.33 | 1.08 | 0.851,1.4 | 0.49 | 0.50 | 0.74 | 0.77 | 0.68 |
| count10 | 5-10mm | CU_AB+ vs AD_AB+ | 1.12 | 0.824,1.49 | 0.46 | 1.04 | 0.768,1.39 | 0.83 | 0.59 | 0.83 | 0.77 | 0.88 |
| count10 | 5-10mm | MCI_AB+ vs AD_AB+ | 0.99 | 0.729,1.32 | 0.93 | 0.95 | 0.699,1.26 | 0.66 | 0.89 | 0.79 | 0.77 | 0.68 |
| count15 | 10-15mm | CU_AB- vs CU_AB+ | 1.24 | 1.02,1.53 | 0.04 | 1.18 | 0.984,1.45 | 0.07 | 0.06 | 0.22 | 0.73 | 0.22 |
| count15 | 10-15mm | CU_AB- vs MCI_AB+ | 1.38 | 1.15,1.7 | 1.37e-3 | 1.28 | 1.07,1.58 | 0.01 | 0.01 | 0.05 | 0.21 | 0.08 |
| count15 | 10-15mm | CU_AB- vs AD_AB+ | 1.33 | 1.04,1.71 | 0.02 | 1.15 | 0.901,1.48 | 0.26 | 0.06 | 0.51 | 0.73 | 0.25 |
| count15 | 10-15mm | CU_AB+ vs MCI_AB+ | 1.12 | 0.891,1.4 | 0.34 | 1.08 | 0.867,1.35 | 0.48 | 0.50 | 0.60 | 0.73 | 0.64 |
| count15 | 10-15mm | CU_AB+ vs AD_AB+ | 1.08 | 0.812,1.4 | 0.60 | 1.00 | 0.754,1.31 | 0.95 | 0.74 | 0.95 | 0.73 | 0.64 |
| count15 | 10-15mm | MCI_AB+ vs AD_AB+ | 0.97 | 0.728,1.25 | 0.80 | 0.92 | 0.694,1.19 | 0.50 | 0.74 | 0.60 | 0.73 | 0.45 |
| count20 | 15-20mm | CU_AB- vs CU_AB+ | 1.15 | 0.933,1.43 | 0.20 | 1.10 | 0.899,1.37 | 0.33 | 0.41 | 0.55 | 0.83 | 0.46 |
| count20 | 15-20mm | CU_AB- vs MCI_AB+ | 1.31 | 1.07,1.63 | 0.01 | 1.25 | 1.03,1.56 | 0.03 | 0.06 | 0.17 | 0.31 | 0.12 |
| count20 | 15-20mm | CU_AB- vs AD_AB+ | 1.19 | 0.912,1.54 | 0.20 | 1.05 | 0.809,1.37 | 0.70 | 0.41 | 0.80 | 0.93 | 0.47 |
| count20 | 15-20mm | CU_AB+ vs MCI_AB+ | 1.14 | 0.895,1.47 | 0.28 | 1.12 | 0.88,1.44 | 0.34 | 0.42 | 0.55 | 0.83 | 0.47 |
| count20 | 15-20mm | CU_AB+ vs AD_AB+ | 1.04 | 0.765,1.38 | 0.82 | 0.97 | 0.714,1.3 | 0.80 | 0.85 | 0.80 | 0.83 | 0.47 |
| count20 | 15-20mm | MCI_AB+ vs AD_AB+ | 0.91 | 0.67,1.2 | 0.50 | 0.88 | 0.65,1.17 | 0.36 | 0.56 | 0.55 | 0.83 | 0.46 |
| count25 | 20-25mm | CU_AB- vs CU_AB+ | 0.95 | 0.767,1.22 | 0.67 | 0.95 | 0.764,1.21 | 0.75 | 0.78 | 0.75 | 0.81 | 0.91 |
| count25 | 20-25mm | CU_AB- vs MCI_AB+ | 1.07 | 0.862,1.37 | 0.56 | 1.05 | 0.843,1.34 | 0.60 | 0.78 | 0.72 | 0.76 | 0.74 |
| count25 | 20-25mm | CU_AB- vs AD_AB+ | 0.91 | 0.683,1.21 | 0.51 | 0.84 | 0.627,1.13 | 0.26 | 0.78 | 0.72 | 0.76 | 0.74 |
| count25 | 20-25mm | CU_AB+ vs MCI_AB+ | 1.13 | 0.853,1.48 | 0.40 | 1.11 | 0.845,1.46 | 0.45 | 0.78 | 0.72 | 0.76 | 0.74 |
| count25 | 20-25mm | CU_AB+ vs AD_AB+ | 0.95 | 0.682,1.3 | 0.78 | 0.91 | 0.645,1.25 | 0.53 | 0.78 | 0.72 | 0.79 | 0.74 |
| count25 | 20-25mm | MCI_AB+ vs AD_AB+ | 0.85 | 0.607,1.16 | 0.32 | 0.83 | 0.589,1.14 | 0.24 | 0.78 | 0.72 | 0.76 | 0.74 |
| count30 | 25-30mm | CU_AB- vs CU_AB+ | 0.71 | 0.55,0.952 | 0.01 | 0.73 | 0.564,0.973 | 0.03 | 0.06 | 0.09 | 0.34 | 0.17 |
| count30 | 25-30mm | CU_AB- vs MCI_AB+ | 0.89 | 0.685,1.19 | 0.41 | 0.89 | 0.684,1.19 | 0.46 | 0.54 | 0.46 | 0.75 | 0.63 |
| count30 | 25-30mm | CU_AB- vs AD_AB+ | 0.64 | 0.453,0.901 | 0.01 | 0.64 | 0.443,0.908 | 0.01 | 0.06 | 0.08 | 0.34 | 0.17 |
| count30 | 25-30mm | CU_AB+ vs MCI_AB+ | 1.25 | 0.887,1.76 | 0.20 | 1.21 | 0.863,1.7 | 0.27 | 0.31 | 0.40 | 0.34 | 0.39 |
| count30 | 25-30mm | CU_AB+ vs AD_AB+ | 0.90 | 0.592,1.32 | 0.60 | 0.86 | 0.562,1.29 | 0.45 | 0.54 | 0.46 | 0.75 | 0.32 |
| count30 | 25-30mm | MCI_AB+ vs AD_AB+ | 0.72 | 0.467,1.07 | 0.12 | 0.74 | 0.472,1.11 | 0.14 | 0.21 | 0.28 | 0.34 | 0.17 |
| count30plus | >30mm | CU_AB- vs CU_AB+ | 0.70 | 0.494,1.01 | 0.05 | 0.73 | 0.516,1.04 | 0.08 | 0.09 | 0.13 | 0.69 | 0.32 |
| count30plus | >30mm | CU_AB- vs MCI_AB+ | 0.71 | 0.498,1.04 | 0.07 | 0.78 | 0.544,1.12 | 0.18 | 0.10 | 0.22 | 0.44 | 0.34 |
| count30plus | >30mm | CU_AB- vs AD_AB+ | 0.33 | 0.207,0.517 | 1.70e-6 | 0.38 | 0.235,0.603 | 0.00 | 0.00 | 0.00 | 0.43 | 0.00 |
| count30plus | >30mm | CU_AB+ vs MCI_AB+ | 1.02 | 0.641,1.61 | 0.93 | 1.16 | 0.737,1.81 | 0.53 | 0.94 | 0.53 | 0.44 | 0.67 |
| count30plus | >30mm | CU_AB+ vs AD_AB+ | 0.47 | 0.273,0.782 | 4.86e-3 | 0.62 | 0.349,1.07 | 0.09 | 0.01 | 0.13 | 0.44 | 0.19 |
| count30plus | >30mm | MCI_AB+ vs AD_AB+ | 0.46 | 0.26,0.795 | 6.45e-3 | 0.53 | 0.296,0.931 | 0.03 | 0.01 | 0.08 | 0.43 | 0.07 |

**S6A: EPVS WMH correlation across distance bins: all cohort**

| **Category** | **Adjustment** | **Distance** | **Effect Size** | **ES CI Lower** | **ES CI Upper** | **p-value** | **FDR p-value** | **FDR p-value (APOEe4+edu edj)** |
| --- | --- | --- | --- | --- | --- | --- | --- | --- |
| pvscountwmh | unadjusted | 0.00 | 1.80 | 1.65 | 1.96 | 1.69e-41 | 3.87e-41 | 0.00 |
| count5 | unadjusted | ≤ <=5mm | 1.97 | 1.84 | 2.10 | 2.87e-85 | 1.53e-84 | 0.00 |
| count10 | unadjusted | 5-10mm | 1.54 | 1.44 | 1.64 | 6.18e-39 | 1.24e-38 | 0.00 |
| count15 | unadjusted | 10-15mm | 1.26 | 1.18 | 1.34 | 1.69e-12 | 2.07e-12 | 0.00 |
| count20 | unadjusted | 15-20mm | 1.06 | 1.00 | 1.13 | 0.07 | 0.07 | 0.07 |
| count25 | unadjusted | 20-25mm | 0.79 | 0.74 | 0.84 | 6.31e-13 | 8.41e-13 | 0.00 |
| count30 | unadjusted | 25-30mm | 0.55 | 0.52 | 0.59 | 3.37e-64 | 1.35e-63 | 0.00 |
| count30plus | unadjusted | > 30mm | 0.40 | 0.37 | 0.43 | 6.18e-119 | 9.89e-118 | 0.00 |
| pvscountwmh | adjusted | 0.00 | 1.69 | 1.54 | 1.87 | 5.56e-27 | 9.88e-27 | 0.00 |
| count5 | adjusted | ≤ <=5mm | 1.92 | 1.78 | 2.08 | 4.39e-63 | 1.40e-62 | 0.00 |
| count10 | adjusted | 5-10mm | 1.41 | 1.31 | 1.52 | 2.67e-20 | 4.27e-20 | 0.00 |
| count15 | adjusted | 10-15mm | 1.16 | 1.08 | 1.25 | 5.74e-5 | 6.56e-5 | 0.00 |
| count20 | adjusted | 15-20mm | 0.97 | 0.91 | 1.05 | 0.46 | 0.46 | 0.37 |
| count25 | adjusted | 20-25mm | 0.73 | 0.68 | 0.79 | 1.25e-16 | 1.81e-16 | 0.00 |
| count30 | adjusted | 25-30mm | 0.52 | 0.48 | 0.56 | 3.20e-62 | 8.54e-62 | 0.00 |
| count30plus | adjusted | > 30mm | 0.39 | 0.35 | 0.42 | 9.20e-101 | 7.36e-100 | 0.00 |

**S6B1: EPVS WMH correlation across distance bins: diagnostic group, interaction effects**

| **Category** | **Group** | **Adjustment** | **Distance** | **ES** | **ES CI Lower** | **ES CI Upper** | **p-value** | **FDR p-value** | **FDR p-value (APOEe4+edu adj)** |
| --- | --- | --- | --- | --- | --- | --- | --- | --- | --- |
| pvscountwmh | CU | unadjusted | 0.00 | 1.70 | 1.51 | 1.91 | 3.11e-18 | 8.30e-18 | 0.00 |
| pvscountwmh | MCI | unadjusted | 0.00 | 2.00 | 1.73 | 2.31 | 0.00 | 0.00 | 0.00 |
| pvscountwmh | AD | unadjusted | 0.00 | 1.50 | 1.15 | 1.96 | 2.76e-3 | 3.48e-3 | 0.00 |
| count5 | CU | unadjusted | ≤ <=5mm | 1.86 | 1.70 | 2.04 | 2.88e-39 | 1.15e-38 | 0.00 |
| count5 | MCI | unadjusted | ≤ <=5mm | 2.12 | 1.89 | 2.38 | 0.00 | 0.00 | 0.00 |
| count5 | AD | unadjusted | ≤ <=5mm | 2.16 | 1.71 | 2.72 | 6.70e-11 | 1.34e-10 | 0.00 |
| count10 | CU | unadjusted | 5-10mm | 1.49 | 1.36 | 1.63 | 1.05e-18 | 3.15e-18 | 0.00 |
| count10 | MCI | unadjusted | 5-10mm | 1.54 | 1.38 | 1.71 | 8.22e-15 | 1.97e-14 | 0.00 |
| count10 | AD | unadjusted | 5-10mm | 1.63 | 1.30 | 2.03 | 1.60e-5 | 2.26e-5 | 0.00 |
| count15 | CU | unadjusted | 10-15mm | 1.26 | 1.15 | 1.38 | 2.20e-7 | 3.78e-7 | 0.00 |
| count15 | MCI | unadjusted | 10-15mm | 1.22 | 1.10 | 1.36 | 2.47e-4 | 3.29e-4 | 0.00 |
| count15 | AD | unadjusted | 10-15mm | 1.25 | 1.00 | 1.55 | 0.05 | 0.05 | 0.05 |
| count20 | CU | unadjusted | 15-20mm | 1.08 | 0.98 | 1.17 | 0.11 | 0.12 | 0.12 |
| count20 | MCI | unadjusted | 15-20mm | 1.02 | 0.91 | 1.13 | 0.78 | 0.82 | 0.82 |
| count20 | AD | unadjusted | 15-20mm | 1.01 | 0.81 | 1.26 | 0.90 | 0.90 | 0.90 |
| count25 | CU | unadjusted | 20-25mm | 0.80 | 0.73 | 0.87 | 6.77e-7 | 1.08e-6 | 0.00 |
| count25 | MCI | unadjusted | 20-25mm | 0.76 | 0.68 | 0.85 | 9.43e-7 | 1.42e-6 | 0.00 |
| count25 | AD | unadjusted | 20-25mm | 0.73 | 0.58 | 0.91 | 5.38e-3 | 6.45e-3 | 0.01 |
| count30 | CU | unadjusted | 25-30mm | 0.57 | 0.52 | 0.62 | 5.67e-33 | 1.94e-32 | 0.00 |
| count30 | MCI | unadjusted | 25-30mm | 0.53 | 0.47 | 0.60 | 0.00 | 0.00 | 0.00 |
| count30 | AD | unadjusted | 25-30mm | 0.52 | 0.41 | 0.66 | 1.11e-7 | 2.05e-7 | 0.00 |
| count30plus | CU | unadjusted | > 30mm | 0.41 | 0.37 | 0.45 | 1.59e-62 | 7.65e-62 | 0.00 |
| count30plus | MCI | unadjusted | > 30mm | 0.39 | 0.34 | 0.45 | 0.00 | 0.00 | 0.00 |
| count30plus | AD | unadjusted | > 30mm | 0.36 | 0.27 | 0.48 | 7.42e-12 | 1.62e-11 | 0.00 |
| pvscountwmh | CU | adjusted | 0.00 | 1.59 | 1.40 | 1.80 | 1.90e-12 | 5.69e-12 | 0.00 |
| pvscountwmh | MCI | adjusted | 0.00 | 1.91 | 1.64 | 2.22 | 0.00 | 0.00 | 0.00 |
| pvscountwmh | AD | adjusted | 0.00 | 1.38 | 1.05 | 1.80 | 0.02 | 0.02 | 0.18 |
| count5 | CU | adjusted | ≤ <=5mm | 1.81 | 1.64 | 2.01 | 2.63e-31 | 9.01e-31 | 0.00 |
| count5 | MCI | adjusted | ≤ <=5mm | 2.08 | 1.85 | 2.35 | 0.00 | 0.00 | 0.00 |
| count5 | AD | adjusted | ≤ <=5mm | 2.10 | 1.66 | 2.65 | 4.99e-10 | 9.97e-10 | 0.00 |
| count10 | CU | adjusted | 5-10mm | 1.36 | 1.24 | 1.50 | 3.07e-10 | 7.37e-10 | 0.00 |
| count10 | MCI | adjusted | 5-10mm | 1.43 | 1.28 | 1.60 | 3.64e-10 | 7.94e-10 | 0.00 |
| count10 | AD | adjusted | 5-10mm | 1.50 | 1.20 | 1.88 | 3.55e-4 | 5.32e-4 | 0.01 |
| count15 | CU | adjusted | 10-15mm | 1.16 | 1.05 | 1.27 | 2.67e-3 | 3.56e-3 | 0.01 |
| count15 | MCI | adjusted | 10-15mm | 1.14 | 1.02 | 1.28 | 0.02 | 0.02 | 0.06 |
| count15 | AD | adjusted | 10-15mm | 1.18 | 0.95 | 1.48 | 0.14 | 0.16 | 0.24 |
| count20 | CU | adjusted | 15-20mm | 0.98 | 0.89 | 1.08 | 0.73 | 0.73 | 0.66 |
| count20 | MCI | adjusted | 15-20mm | 0.95 | 0.85 | 1.06 | 0.32 | 0.35 | 0.31 |
| count20 | AD | adjusted | 15-20mm | 0.94 | 0.76 | 1.18 | 0.60 | 0.63 | 0.61 |
| count25 | CU | adjusted | 20-25mm | 0.74 | 0.67 | 0.82 | 8.26e-10 | 1.53e-9 | 0.00 |
| count25 | MCI | adjusted | 20-25mm | 0.72 | 0.65 | 0.81 | 1.66e-8 | 2.84e-8 | 0.00 |
| count25 | AD | adjusted | 20-25mm | 0.69 | 0.55 | 0.87 | 1.53e-3 | 2.16e-3 | 0.01 |
| count30 | CU | adjusted | 25-30mm | 0.53 | 0.48 | 0.58 | 1.97e-35 | 7.89e-35 | 0.00 |
| count30 | MCI | adjusted | 25-30mm | 0.51 | 0.45 | 0.57 | 0.00 | 0.00 | 0.00 |
| count30 | AD | adjusted | 25-30mm | 0.50 | 0.39 | 0.64 | 2.39e-8 | 3.82e-8 | 0.00 |
| count30plus | CU | adjusted | > 30mm | 0.40 | 0.35 | 0.44 | 2.13e-57 | 1.02e-56 | 0.00 |
| count30plus | MCI | adjusted | > 30mm | 0.38 | 0.34 | 0.44 | 0.00 | 0.00 | 0.00 |
| count30plus | AD | adjusted | > 30mm | 0.36 | 0.26 | 0.48 | 5.76e-12 | 1.54e-11 | 0.00 |

**S6B2: EPVS WMH correlation across distance bins: diagnostic group, interaction effects**

| **Category** | **Group** | **Adjustment** | **Distance** | **p Interact** | **Interact CI Lower** | **Interact CI Upper** | **Interact ES** | **ES CI Lower** | **ES CI Upper** | **FDR p Interact** | **FDR p Interact (APOEe4+edu adj)** |
| --- | --- | --- | --- | --- | --- | --- | --- | --- | --- | --- | --- |
| pvscountwmh | MCI | unadjusted | 0.00 | 0.09 | -0.02 | 0.35 | 1.18 | 0.98 | 1.42 | 0.70 | 0.70 |
| pvscountwmh | AD | unadjusted | 0.00 | 0.41 | -0.41 | 0.17 | 0.89 | 0.66 | 1.19 | 0.73 | 0.73 |
| count5 | MCI | unadjusted | ≤5mm | 0.08 | -0.02 | 0.28 | 1.14 | 0.98 | 1.32 | 0.70 | 0.70 |
| count5 | AD | unadjusted | ≤ 5mm | 0.24 | -0.10 | 0.40 | 1.16 | 0.90 | 1.49 | 0.73 | 0.73 |
| count10 | MCI | unadjusted | 5-10mm | 0.68 | -0.11 | 0.17 | 1.03 | 0.90 | 1.18 | 0.73 | 0.73 |
| count10 | AD | unadjusted | 5-10mm | 0.48 | -0.15 | 0.32 | 1.09 | 0.86 | 1.38 | 0.73 | 0.73 |
| count15 | MCI | unadjusted | 10-15mm | 0.66 | -0.17 | 0.11 | 0.97 | 0.84 | 1.11 | 0.73 | 0.73 |
| count15 | AD | unadjusted | 10-15mm | 0.93 | -0.25 | 0.23 | 0.99 | 0.78 | 1.25 | 0.93 | 0.93 |
| count20 | MCI | unadjusted | 15-20mm | 0.42 | -0.20 | 0.08 | 0.94 | 0.82 | 1.08 | 0.73 | 0.73 |
| count20 | AD | unadjusted | 15-20mm | 0.63 | -0.29 | 0.18 | 0.94 | 0.75 | 1.19 | 0.73 | 0.73 |
| count25 | MCI | unadjusted | 20-25mm | 0.51 | -0.19 | 0.09 | 0.95 | 0.83 | 1.10 | 0.73 | 0.73 |
| count25 | AD | unadjusted | 20-25mm | 0.45 | -0.33 | 0.15 | 0.91 | 0.72 | 1.16 | 0.73 | 0.73 |
| count30 | MCI | unadjusted | 25-30mm | 0.43 | -0.21 | 0.09 | 0.94 | 0.81 | 1.09 | 0.73 | 0.73 |
| count30 | AD | unadjusted | 25-30mm | 0.53 | -0.34 | 0.18 | 0.92 | 0.71 | 1.19 | 0.73 | 0.73 |
| count30plus | MCI | unadjusted | > 30mm | 0.60 | -0.21 | 0.12 | 0.96 | 0.81 | 1.13 | 0.73 | 0.73 |
| count30plus | AD | unadjusted | > 30mm | 0.44 | -0.43 | 0.19 | 0.88 | 0.65 | 1.21 | 0.73 | 0.73 |
| pvscountwmh | MCI | adjusted | 0.00 | 0.05 | -2.68e-3 | 0.37 | 1.20 | 1.00 | 1.45 | 0.52 | 0.91 |
| pvscountwmh | AD | adjusted | 0.00 | 0.34 | -0.43 | 0.15 | 0.87 | 0.65 | 1.16 | 0.84 | 0.91 |
| count5 | MCI | adjusted | ≤ <=5mm | 0.07 | -8.83e-3 | 0.29 | 1.15 | 0.99 | 1.33 | 0.52 | 0.91 |
| count5 | AD | adjusted | ≤ <=5mm | 0.25 | -0.10 | 0.39 | 1.16 | 0.90 | 1.48 | 0.84 | 0.98 |
| count10 | MCI | adjusted | 5-10mm | 0.48 | -0.09 | 0.19 | 1.05 | 0.91 | 1.21 | 0.84 | 0.91 |
| count10 | AD | adjusted | 5-10mm | 0.41 | -0.14 | 0.34 | 1.10 | 0.87 | 1.40 | 0.84 | 0.91 |
| count15 | MCI | adjusted | 10-15mm | 0.88 | -0.15 | 0.13 | 0.99 | 0.86 | 1.14 | 0.88 | 0.91 |
| count15 | AD | adjusted | 10-15mm | 0.85 | -0.21 | 0.26 | 1.02 | 0.81 | 1.30 | 0.88 | 0.91 |
| count20 | MCI | adjusted | 15-20mm | 0.58 | -0.18 | 0.10 | 0.96 | 0.84 | 1.10 | 0.84 | 0.91 |
| count20 | AD | adjusted | 15-20mm | 0.73 | -0.28 | 0.19 | 0.96 | 0.76 | 1.21 | 0.84 | 0.91 |
| count25 | MCI | adjusted | 20-25mm | 0.73 | -0.16 | 0.12 | 0.98 | 0.85 | 1.12 | 0.84 | 0.91 |
| count25 | AD | adjusted | 20-25mm | 0.60 | -0.31 | 0.18 | 0.94 | 0.74 | 1.19 | 0.84 | 0.91 |
| count30 | MCI | adjusted | 25-30mm | 0.61 | -0.19 | 0.11 | 0.96 | 0.83 | 1.12 | 0.84 | 0.91 |
| count30 | AD | adjusted | 25-30mm | 0.68 | -0.31 | 0.21 | 0.95 | 0.73 | 1.23 | 0.84 | 0.91 |
| count30plus | MCI | adjusted | > 30mm | 0.70 | -0.20 | 0.14 | 0.97 | 0.82 | 1.14 | 0.84 | 0.91 |
| count30plus | AD | adjusted | > 30mm | 0.48 | -0.42 | 0.20 | 0.89 | 0.66 | 1.22 | 0.84 | 0.91 |

**S6C1: EPVS WMH correlation across distance bins: amyloid, interaction effect**

| **Category** | **Group** | **Adjustment** | **Distance** | **ES** | **ES CI Lower** | **ES CI Upper** | **p-value** | **FDR p-value** | **FDR p-value (APOEe4+edu adj)** |
| --- | --- | --- | --- | --- | --- | --- | --- | --- | --- |
| pvscountwmh | AB- | unadjusted | 0.00 | 1.85 | 1.64 | 2.08 | 1.00e-24 | 2.01e-24 | 0.00 |
| pvscountwmh | AB+ | unadjusted | 0.00 | 1.56 | 1.34 | 1.81 | 4.49e-9 | 7.18e-9 | 0.00 |
| count5 | AB- | unadjusted | ≤ <=5mm | 2.09 | 1.91 | 2.30 | 2.96e-53 | 9.49e-53 | 0.00 |
| count5 | AB+ | unadjusted | ≤ <=5mm | 1.76 | 1.56 | 1.98 | 0.00 | 0.00 | 0.00 |
| count10 | AB- | unadjusted | 5-10mm | 1.61 | 1.48 | 1.77 | 4.24e-26 | 9.69e-26 | 0.00 |
| count10 | AB+ | unadjusted | 5-10mm | 1.31 | 1.17 | 1.47 | 3.51e-6 | 4.32e-6 | 0.00 |
| count15 | AB- | unadjusted | 10-15mm | 1.31 | 1.20 | 1.43 | 1.62e-9 | 2.89e-9 | 0.00 |
| count15 | AB+ | unadjusted | 10-15mm | 1.09 | 0.97 | 1.22 | 0.15 | 0.16 | 0.15 |
| count20 | AB- | unadjusted | 15-20mm | 1.10 | 1.01 | 1.20 | 0.03 | 0.03 | 0.03 |
| count20 | AB+ | unadjusted | 15-20mm | 0.97 | 0.86 | 1.08 | 0.57 | 0.57 | 0.57 |
| count25 | AB- | unadjusted | 20-25mm | 0.81 | 0.74 | 0.88 | 2.15e-6 | 3.06e-6 | 0.00 |
| count25 | AB+ | unadjusted | 20-25mm | 0.75 | 0.67 | 0.85 | 2.30e-6 | 3.06e-6 | 0.00 |
| count30 | AB- | unadjusted | 25-30mm | 0.55 | 0.50 | 0.60 | 6.25e-37 | 1.67e-36 | 0.00 |
| count30 | AB+ | unadjusted | 25-30mm | 0.57 | 0.50 | 0.65 | 0.00 | 0.00 | 0.00 |
| count30plus | AB- | unadjusted | > 30mm | 0.38 | 0.34 | 0.42 | 1.05e-71 | 4.20e-71 | 0.00 |
| count30plus | AB+ | unadjusted | > 30mm | 0.46 | 0.40 | 0.53 | 0.00 | 0.00 | 0.00 |
| pvscountwmh | AB- | adjusted | 0.00 | 1.74 | 1.54 | 1.97 | 4.01e-18 | 1.07e-17 | 0.00 |
| pvscountwmh | AB+ | adjusted | 0.00 | 1.46 | 1.24 | 1.70 | 2.91e-6 | 4.23e-6 | 0.00 |
| count5 | AB- | adjusted | ≤ <=5mm | 2.03 | 1.83 | 2.24 | 4.25e-43 | 1.70e-42 | 0.00 |
| count5 | AB+ | adjusted | ≤ <=5mm | 1.71 | 1.50 | 1.94 | 2.22e-16 | 5.08e-16 | 0.00 |
| count10 | AB- | adjusted | 5-10mm | 1.48 | 1.34 | 1.62 | 1.01e-15 | 2.03e-15 | 0.00 |
| count10 | AB+ | adjusted | 5-10mm | 1.21 | 1.07 | 1.37 | 1.82e-3 | 2.24e-3 | 0.00 |
| count15 | AB- | adjusted | 10-15mm | 1.21 | 1.10 | 1.33 | 6.33e-5 | 8.44e-5 | 0.00 |
| count15 | AB+ | adjusted | 10-15mm | 1.01 | 0.90 | 1.14 | 0.83 | 0.83 | 0.96 |
| count20 | AB- | adjusted | 15-20mm | 1.01 | 0.92 | 1.11 | 0.80 | 0.83 | 0.86 |
| count20 | AB+ | adjusted | 15-20mm | 0.90 | 0.79 | 1.01 | 0.08 | 0.09 | 0.06 |
| count25 | AB- | adjusted | 20-25mm | 0.75 | 0.69 | 0.83 | 6.14e-9 | 1.09e-8 | 0.00 |
| count25 | AB+ | adjusted | 20-25mm | 0.71 | 0.62 | 0.80 | 4.02e-8 | 6.44e-8 | 0.00 |
| count30 | AB- | adjusted | 25-30mm | 0.51 | 0.46 | 0.57 | 1.38e-38 | 4.41e-38 | 0.00 |
| count30 | AB+ | adjusted | 25-30mm | 0.53 | 0.47 | 0.61 | 0.00 | 0.00 | 0.00 |
| count30plus | AB- | adjusted | > 30mm | 0.37 | 0.33 | 0.42 | 6.00e-65 | 3.20e-64 | 0.00 |
| count30plus | AB+ | adjusted | > 30mm | 0.44 | 0.38 | 0.51 | 0.00 | 0.00 | 0.00 |

**S6C2: EPVS WMH correlation across distance bins: amyloid, interaction effect**

| **Category** | **Group** | **Adjustment** | **Distance** | **p Interact** | **Interact ES** | **ES CI Lower** | **ES CI Upper** | **FDR p Interact** | **FDR p Interact (APOEe4+edu adj)** |
| --- | --- | --- | --- | --- | --- | --- | --- | --- | --- |
| pvscountwmh | AB+ | unadjusted | 0.00 | 0.08 | 0.84 | 0.70 | 1.02 | 0.10 | 0.10 |
| count5 | AB+ | unadjusted | ≤ <=5mm | 0.03 | 0.84 | 0.72 | 0.98 | 0.07 | 0.07 |
| count10 | AB+ | unadjusted | 5-10mm | 5.35e-3 | 0.81 | 0.70 | 0.94 | 0.04 | 0.04 |
| count15 | AB+ | unadjusted | 10-15mm | 0.01 | 0.83 | 0.72 | 0.96 | 0.05 | 0.05 |
| count20 | AB+ | unadjusted | 15-20mm | 0.08 | 0.88 | 0.76 | 1.01 | 0.10 | 0.10 |
| count25 | AB+ | unadjusted | 20-25mm | 0.36 | 0.93 | 0.81 | 1.08 | 0.42 | 0.42 |
| count30 | AB+ | unadjusted | 25-30mm | 0.59 | 1.04 | 0.89 | 1.22 | 0.59 | 0.59 |
| count30plus | AB+ | unadjusted | > 30mm | 0.03 | 1.21 | 1.01 | 1.44 | 0.07 | 0.07 |
| pvscountwmh | AB+ | adjusted | 0.00 | 0.06 | 0.84 | 0.69 | 1.01 | 0.10 | 0.12 |
| count5 | AB+ | adjusted | ≤ <=5mm | 0.03 | 0.84 | 0.72 | 0.98 | 0.07 | 0.06 |
| count10 | AB+ | adjusted | 5-10mm | 8.49e-3 | 0.82 | 0.71 | 0.95 | 0.06 | 0.05 |
| count15 | AB+ | adjusted | 10-15mm | 0.02 | 0.84 | 0.72 | 0.97 | 0.06 | 0.05 |
| count20 | AB+ | adjusted | 15-20mm | 0.10 | 0.89 | 0.77 | 1.02 | 0.13 | 0.12 |
| count25 | AB+ | adjusted | 20-25mm | 0.39 | 0.94 | 0.81 | 1.09 | 0.44 | 0.34 |
| count30 | AB+ | adjusted | 25-30mm | 0.61 | 1.04 | 0.89 | 1.22 | 0.61 | 0.65 |
| count30plus | AB+ | adjusted | > 30mm | 0.06 | 1.18 | 0.99 | 1.42 | 0.10 | 0.12 |

**S6D1: EPVS WMH correlation across distance bins: biomarker groups, main effect**

| **Category** | **Group** | **Adjustment** | **Distance** | **ES** | **ES CI Lower** | **ES CI Upper** | **p-value** | **FDR p-value** | **FDR p-value (APOEe4+edu adj)** |
| --- | --- | --- | --- | --- | --- | --- | --- | --- | --- |
| pvscountwmh | CU AB- | unadjusted | 0.00 | 1.60 | 1.41 | 1.82 | 3.96e-13 | 1.58e-12 | 0.00 |
| pvscountwmh | CU_AB+ | unadjusted | 0.00 | 1.71 | 1.36 | 2.15 | 4.31e-6 | 1.06e-5 | 0.00 |
| pvscountwmh | MCI_AB+ | unadjusted | 0.00 | 1.64 | 1.30 | 2.08 | 2.84e-5 | 5.05e-5 | 0.00 |
| pvscountwmh | AD_AB+ | unadjusted | 0.00 | 1.17 | 0.84 | 1.62 | 0.37 | 0.42 | 0.37 |
| count5 | CU AB- | unadjusted | ≤ <=5mm | 1.97 | 1.78 | 2.18 | 3.61e-39 | 5.77e-38 | 0.00 |
| count5 | CU_AB+ | unadjusted | ≤ <=5mm | 1.92 | 1.61 | 2.28 | 2.14e-13 | 9.80e-13 | 0.00 |
| count5 | MCI_AB+ | unadjusted | ≤ <=5mm | 1.73 | 1.44 | 2.07 | 3.27e-9 | 1.05e-8 | 0.00 |
| count5 | AD_AB+ | unadjusted | ≤ <=5mm | 1.64 | 1.23 | 2.18 | 6.71e-4 | 1.02e-3 | 0.00 |
| count10 | CU AB- | unadjusted | 5-10mm | 1.58 | 1.45 | 1.73 | 9.70e-24 | 1.03e-22 | 0.00 |
| count10 | CU_AB+ | unadjusted | 5-10mm | 1.49 | 1.29 | 1.72 | 2.75e-8 | 7.33e-8 | 0.00 |
| count10 | MCI_AB+ | unadjusted | 5-10mm | 1.34 | 1.13 | 1.60 | 7.42e-4 | 1.08e-3 | 0.00 |
| count10 | AD_AB+ | unadjusted | 5-10mm | 1.25 | 0.95 | 1.65 | 0.12 | 0.15 | 0.12 |
| count15 | CU AB- | unadjusted | 10-15mm | 1.35 | 1.24 | 1.47 | 7.36e-12 | 2.62e-11 | 0.00 |
| count15 | CU_AB+ | unadjusted | 10-15mm | 1.35 | 1.18 | 1.54 | 8.85e-6 | 2.02e-5 | 0.00 |
| count15 | MCI_AB+ | unadjusted | 10-15mm | 1.05 | 0.89 | 1.25 | 0.56 | 0.60 | 0.56 |
| count15 | AD_AB+ | unadjusted | 10-15mm | 1.07 | 0.81 | 1.41 | 0.64 | 0.66 | 0.64 |
| count20 | CU AB- | unadjusted | 15-20mm | 1.19 | 1.10 | 1.30 | 4.02e-5 | 6.77e-5 | 0.00 |
| count20 | CU_AB+ | unadjusted | 15-20mm | 1.27 | 1.11 | 1.44 | 4.14e-4 | 6.63e-4 | 0.00 |
| count20 | MCI_AB+ | unadjusted | 15-20mm | 0.91 | 0.77 | 1.08 | 0.27 | 0.32 | 0.27 |
| count20 | AD_AB+ | unadjusted | 15-20mm | 0.90 | 0.68 | 1.19 | 0.46 | 0.51 | 0.46 |
| count25 | CU AB- | unadjusted | 20-25mm | 0.94 | 0.87 | 1.03 | 0.17 | 0.22 | 0.17 |
| count25 | CU_AB+ | unadjusted | 20-25mm | 0.98 | 0.86 | 1.11 | 0.76 | 0.76 | 0.76 |
| count25 | MCI_AB+ | unadjusted | 20-25mm | 0.78 | 0.65 | 0.92 | 4.18e-3 | 5.57e-3 | 0.00 |
| count25 | AD_AB+ | unadjusted | 20-25mm | 0.62 | 0.46 | 0.82 | 1.02e-3 | 1.42e-3 | 0.00 |
| count30 | CU AB- | unadjusted | 25-30mm | 0.69 | 0.64 | 0.76 | 8.86e-17 | 7.09e-16 | 0.00 |
| count30 | CU_AB+ | unadjusted | 25-30mm | 0.76 | 0.67 | 0.86 | 2.67e-5 | 5.04e-5 | 0.00 |
| count30 | MCI_AB+ | unadjusted | 25-30mm | 0.60 | 0.50 | 0.71 | 2.01e-8 | 5.85e-8 | 0.00 |
| count30 | AD_AB+ | unadjusted | 25-30mm | 0.49 | 0.36 | 0.68 | 1.08e-5 | 2.31e-5 | 0.00 |
| count30plus | CU AB- | unadjusted | > 30mm | 0.46 | 0.42 | 0.51 | 1.99e-59 | 6.38e-58 | 0.00 |
| count30plus | CU_AB+ | unadjusted | > 30mm | 0.58 | 0.51 | 0.66 | 1.33e-15 | 8.53e-15 | 0.00 |
| count30plus | MCI_AB+ | unadjusted | > 30mm | 0.46 | 0.37 | 0.56 | 3.82e-14 | 2.04e-13 | 0.00 |
| count30plus | AD_AB+ | unadjusted | > 30mm | 0.43 | 0.29 | 0.63 | 1.51e-5 | 3.03e-5 | 0.00 |
| pvscountwmh | CU AB- | adjusted | 0.00 | 1.51 | 1.31 | 1.73 | 4.08e-9 | 1.45e-8 | 0.00 |
| pvscountwmh | CU_AB+ | adjusted | 0.00 | 1.61 | 1.27 | 2.04 | 6.77e-5 | 1.35e-4 | 0.00 |
| pvscountwmh | MCI_AB+ | adjusted | 0.00 | 1.54 | 1.21 | 1.95 | 4.84e-4 | 8.16e-4 | 0.00 |
| pvscountwmh | AD_AB+ | adjusted | 0.00 | 1.04 | 0.75 | 1.46 | 0.80 | 0.85 | 0.59 |
| count5 | CU AB- | adjusted | ≤ <=5mm | 1.91 | 1.72 | 2.13 | 1.09e-31 | 1.74e-30 | 0.00 |
| count5 | CU_AB+ | adjusted | ≤ <=5mm | 1.86 | 1.56 | 2.22 | 9.59e-12 | 4.39e-11 | 0.00 |
| count5 | MCI_AB+ | adjusted | ≤ <=5mm | 1.67 | 1.38 | 2.01 | 8.11e-8 | 2.60e-7 | 0.00 |
| count5 | AD_AB+ | adjusted | ≤ <=5mm | 1.57 | 1.18 | 2.09 | 2.09e-3 | 3.19e-3 | 0.00 |
| count10 | CU AB- | adjusted | 5-10mm | 1.48 | 1.34 | 1.62 | 2.31e-15 | 1.48e-14 | 0.00 |
| count10 | CU_AB+ | adjusted | 5-10mm | 1.40 | 1.22 | 1.62 | 3.24e-6 | 7.98e-6 | 0.00 |
| count10 | MCI_AB+ | adjusted | 5-10mm | 1.25 | 1.04 | 1.49 | 0.01 | 0.02 | 0.02 |
| count10 | AD_AB+ | adjusted | 5-10mm | 1.14 | 0.86 | 1.51 | 0.37 | 0.42 | 0.34 |
| count15 | CU AB- | adjusted | 10-15mm | 1.27 | 1.16 | 1.40 | 2.97e-7 | 8.64e-7 | 0.00 |
| count15 | CU_AB+ | adjusted | 10-15mm | 1.28 | 1.12 | 1.46 | 3.79e-4 | 6.74e-4 | 0.00 |
| count15 | MCI_AB+ | adjusted | 10-15mm | 0.99 | 0.83 | 1.18 | 0.88 | 0.88 | 0.80 |
| count15 | AD_AB+ | adjusted | 10-15mm | 1.02 | 0.77 | 1.35 | 0.88 | 0.88 | 0.82 |
| count20 | CU AB- | adjusted | 15-20mm | 1.12 | 1.03 | 1.23 | 0.01 | 0.02 | 0.01 |
| count20 | CU_AB+ | adjusted | 15-20mm | 1.20 | 1.05 | 1.37 | 7.70e-3 | 0.01 | 0.01 |
| count20 | MCI_AB+ | adjusted | 15-20mm | 0.86 | 0.72 | 1.02 | 0.09 | 0.11 | 0.07 |
| count20 | AD_AB+ | adjusted | 15-20mm | 0.85 | 0.64 | 1.12 | 0.25 | 0.30 | 0.34 |
| count25 | CU AB- | adjusted | 20-25mm | 0.90 | 0.82 | 0.98 | 0.02 | 0.03 | 0.03 |
| count25 | CU_AB+ | adjusted | 20-25mm | 0.94 | 0.83 | 1.07 | 0.38 | 0.42 | 0.30 |
| count25 | MCI_AB+ | adjusted | 20-25mm | 0.74 | 0.62 | 0.88 | 8.79e-4 | 1.41e-3 | 0.00 |
| count25 | AD_AB+ | adjusted | 20-25mm | 0.58 | 0.44 | 0.78 | 2.73e-4 | 5.14e-4 | 0.00 |
| count30 | CU AB- | adjusted | 25-30mm | 0.66 | 0.60 | 0.73 | 5.75e-18 | 6.13e-17 | 0.00 |
| count30 | CU_AB+ | adjusted | 25-30mm | 0.73 | 0.64 | 0.83 | 2.04e-6 | 5.44e-6 | 0.00 |
| count30 | MCI_AB+ | adjusted | 25-30mm | 0.56 | 0.47 | 0.68 | 1.77e-9 | 7.07e-9 | 0.00 |
| count30 | AD_AB+ | adjusted | 25-30mm | 0.47 | 0.34 | 0.65 | 3.57e-6 | 8.15e-6 | 0.00 |
| count30plus | CU AB- | adjusted | > 30mm | 0.46 | 0.41 | 0.51 | 1.78e-52 | 5.69e-51 | 0.00 |
| count30plus | CU_AB+ | adjusted | > 30mm | 0.57 | 0.50 | 0.65 | 1.11e-15 | 8.88e-15 | 0.00 |
| count30plus | MCI_AB+ | adjusted | > 30mm | 0.44 | 0.36 | 0.54 | 1.58e-14 | 8.41e-14 | 0.00 |
| count30plus | AD_AB+ | adjusted | > 30mm | 0.41 | 0.28 | 0.61 | 8.15e-6 | 1.74e-5 | 0.00 |

**S6D2: EPVS WMH correlation across distance bins: biomarker groups, interaction**

| **Category** | **Group** | **Adjustment** | **Distance** | **p Interact** | **Interact ES** | **ES CI Lower** | **ES CI Upper** | **FDR p Interact** | **FDR p Interact (APOEe4+edu adj)** |
| --- | --- | --- | --- | --- | --- | --- | --- | --- | --- |
| pvscountwmh | CU_AB+ | unadjusted | 0.00 | 0.63 | 1.07 | 0.82 | 1.39 | 0.79 | 0.79 |
| pvscountwmh | MCI_AB+ | unadjusted | 0.00 | 0.86 | 1.02 | 0.79 | 1.34 | 0.93 | 0.93 |
| pvscountwmh | AD_AB+ | unadjusted | 0.00 | 0.08 | 0.73 | 0.51 | 1.04 | 0.23 | 0.23 |
| count5 | CU_AB+ | unadjusted | ≤ <=5mm | 0.78 | 0.97 | 0.79 | 1.19 | 0.89 | 0.89 |
| count5 | MCI_AB+ | unadjusted | ≤ <=5mm | 0.21 | 0.88 | 0.71 | 1.08 | 0.39 | 0.39 |
| count5 | AD_AB+ | unadjusted | ≤ <=5mm | 0.23 | 0.83 | 0.61 | 1.12 | 0.39 | 0.39 |
| count10 | CU_AB+ | unadjusted | 5-10mm | 0.49 | 0.94 | 0.80 | 1.11 | 0.68 | 0.68 |
| count10 | MCI_AB+ | unadjusted | 5-10mm | 0.10 | 0.85 | 0.70 | 1.03 | 0.25 | 0.25 |
| count10 | AD_AB+ | unadjusted | 5-10mm | 0.11 | 0.79 | 0.59 | 1.06 | 0.25 | 0.25 |
| count15 | CU_AB+ | unadjusted | 10-15mm | 0.99 | 1.00 | 0.86 | 1.17 | 0.99 | 0.99 |
| count15 | MCI_AB+ | unadjusted | 10-15mm | 0.01 | 0.78 | 0.65 | 0.94 | 0.06 | 0.06 |
| count15 | AD_AB+ | unadjusted | 10-15mm | 0.11 | 0.79 | 0.59 | 1.06 | 0.25 | 0.25 |
| count20 | CU_AB+ | unadjusted | 15-20mm | 0.46 | 1.06 | 0.91 | 1.24 | 0.68 | 0.68 |
| count20 | MCI_AB+ | unadjusted | 15-20mm | 4.97e-3 | 0.76 | 0.63 | 0.92 | 0.06 | 0.06 |
| count20 | AD_AB+ | unadjusted | 15-20mm | 0.06 | 0.75 | 0.56 | 1.01 | 0.20 | 0.20 |
| count25 | CU_AB+ | unadjusted | 20-25mm | 0.61 | 1.04 | 0.89 | 1.21 | 0.79 | 0.79 |
| count25 | MCI_AB+ | unadjusted | 20-25mm | 0.05 | 0.82 | 0.68 | 1.00 | 0.19 | 0.19 |
| count25 | AD_AB+ | unadjusted | 20-25mm | 5.59e-3 | 0.66 | 0.49 | 0.88 | 0.06 | 0.06 |
| count30 | CU_AB+ | unadjusted | 25-30mm | 0.25 | 1.09 | 0.94 | 1.28 | 0.40 | 0.40 |
| count30 | MCI_AB+ | unadjusted | 25-30mm | 0.13 | 0.86 | 0.70 | 1.05 | 0.26 | 0.26 |
| count30 | AD_AB+ | unadjusted | 25-30mm | 0.04 | 0.71 | 0.51 | 0.98 | 0.19 | 0.19 |
| count30plus | CU_AB+ | unadjusted | > 30mm | 7.89e-3 | 1.25 | 1.06 | 1.47 | 0.06 | 0.06 |
| count30plus | MCI_AB+ | unadjusted | > 30mm | 0.91 | 0.99 | 0.79 | 1.23 | 0.95 | 0.95 |
| count30plus | AD_AB+ | unadjusted | > 30mm | 0.70 | 0.92 | 0.62 | 1.37 | 0.84 | 0.84 |
| pvscountwmh | CU_AB+ | adjusted | 0.00 | 0.61 | 1.07 | 0.82 | 1.39 | 0.74 | 0.81 |
| pvscountwmh | MCI_AB+ | adjusted | 0.00 | 0.89 | 1.02 | 0.78 | 1.33 | 0.92 | 0.95 |
| pvscountwmh | AD_AB+ | adjusted | 0.00 | 0.04 | 0.69 | 0.49 | 0.99 | 0.16 | 0.18 |
| count5 | CU_AB+ | adjusted | ≤ <=5mm | 0.78 | 0.97 | 0.80 | 1.19 | 0.85 | 0.84 |
| count5 | MCI_AB+ | adjusted | ≤ <=5mm | 0.20 | 0.87 | 0.71 | 1.07 | 0.34 | 0.27 |
| count5 | AD_AB+ | adjusted | ≤ <=5mm | 0.20 | 0.82 | 0.61 | 1.11 | 0.34 | 0.36 |
| count10 | CU_AB+ | adjusted | 5-10mm | 0.56 | 0.95 | 0.81 | 1.12 | 0.74 | 0.67 |
| count10 | MCI_AB+ | adjusted | 5-10mm | 0.09 | 0.85 | 0.70 | 1.03 | 0.22 | 0.18 |
| count10 | AD_AB+ | adjusted | 5-10mm | 0.08 | 0.77 | 0.58 | 1.03 | 0.21 | 0.18 |
| count15 | CU_AB+ | adjusted | 10-15mm | 0.96 | 1.00 | 0.86 | 1.17 | 0.96 | 0.90 |
| count15 | MCI_AB+ | adjusted | 10-15mm | 8.66e-3 | 0.77 | 0.64 | 0.94 | 0.05 | 0.04 |
| count15 | AD_AB+ | adjusted | 10-15mm | 0.14 | 0.80 | 0.60 | 1.07 | 0.27 | 0.27 |
| count20 | CU_AB+ | adjusted | 15-20mm | 0.42 | 1.07 | 0.91 | 1.24 | 0.63 | 0.76 |
| count20 | MCI_AB+ | adjusted | 15-20mm | 5.16e-3 | 0.76 | 0.63 | 0.92 | 0.05 | 0.04 |
| count20 | AD_AB+ | adjusted | 15-20mm | 0.06 | 0.75 | 0.56 | 1.01 | 0.17 | 0.18 |
| count25 | CU_AB+ | adjusted | 20-25mm | 0.51 | 1.05 | 0.90 | 1.22 | 0.72 | 0.80 |
| count25 | MCI_AB+ | adjusted | 20-25mm | 0.05 | 0.82 | 0.68 | 1.00 | 0.16 | 0.11 |
| count25 | AD_AB+ | adjusted | 20-25mm | 4.76e-3 | 0.65 | 0.48 | 0.88 | 0.05 | 0.04 |
| count30 | CU_AB+ | adjusted | 25-30mm | 0.24 | 1.10 | 0.94 | 1.28 | 0.39 | 0.52 |
| count30 | MCI_AB+ | adjusted | 25-30mm | 0.11 | 0.85 | 0.69 | 1.04 | 0.24 | 0.18 |
| count30 | AD_AB+ | adjusted | 25-30mm | 0.04 | 0.71 | 0.51 | 0.98 | 0.16 | 0.18 |
| count30plus | CU_AB+ | adjusted | > 30mm | 8.95e-3 | 1.24 | 1.06 | 1.47 | 0.05 | 0.04 |
| count30plus | MCI_AB+ | adjusted | > 30mm | 0.75 | 0.96 | 0.77 | 1.21 | 0.85 | 0.80 |
| count30plus | AD_AB+ | adjusted | > 30mm | 0.60 | 0.90 | 0.60 | 1.34 | 0.74 | 0.76 |

**S7A: WMH comparisons: diagnostic**

| **Outcome** | **Measure** | **Comparison** | **ES Unadj** | **ES CI Lower Unadj** | **ES CI Upper Unadj** | **p Unadj** | **CI Adj** | **ES Adj** | **ES CI Lower Adj** | **ES CI Upper Adj** | **p Adj** | **FDR p Unadj** | **FDR p Adj** | **FDR p (APOEe4+edu adj)** |
| --- | --- | --- | --- | --- | --- | --- | --- | --- | --- | --- | --- | --- | --- | --- |
| volume | wmhVOLnormlog | CU vs MCI | 0.15 | 0.09 | 0.21 | 3.20e-6 | 0.10 | 0.10 | 0.04 | 0.15 | 7.12e-4 | 4.80e-6 | 1.07e-3 | 0.00 |
| volume | wmhVOLnormlog | CU vs AD | 0.22 | 0.15 | 0.29 | 1.78e-9 | 0.22 | 0.13 | 0.06 | 0.19 | 8.43e-5 | 5.35e-9 | 2.53e-4 | 0.00 |
| volume | wmhVOLnormlog | MCI vs AD | 0.15 | 0.06 | 0.24 | 1.03e-3 | 0.04 | 0.10 | 0.01 | 0.18 | 0.02 | 1.03e-3 | 0.02 | 0.01 |

**S7B: WMH comparisons: amyloid**

| **Outcome** | **Measure** | **Comparison** | **Coef Unadj** | **SE Unadj** | **CI Lower Unadj** | **CI Upper Unadj** | **CI Unadj** | **ES Unadj** | **ES CI Lower Unadj** | **ES CI Upper Unadj** | **p Unadj** | **ES Adj** | **Coef Adj** | **SE Adj** | **CI Lower Adj** | **CI Upper Adj** | **CI Adj** | **ES CI Lower Adj** | **ES CI Upper Adj** | **p Adj** | **FDR p Unadj** | **FDR p Adj** | **FDR p (APOEe4+edu adj)** |
| --- | --- | --- | --- | --- | --- | --- | --- | --- | --- | --- | --- | --- | --- | --- | --- | --- | --- | --- | --- | --- | --- | --- | --- |
| volume | wmhVOLnormlog | AB- vs AB+ | 0.46 | 0.08 | 0.30 | 0.62 | 0.30 | 0.18 | 0.12 | 0.25 | 1.10e-8 | 0.12 | 0.29 | 0.07 | 0.15 | 0.43 | 0.15 | 0.06 | 0.17 | 4.22e-5 | 1.10e-8 | 4.22e-5 | 0.00 |

**S7C: WMH comparisons: biomarker**

| **Outcome** | **Measure** | **Comparison** | **ES Unadj** | **ES CI Lower Unadj** | **ES CI Upper Unadj** | **p Unadj** | **ES Adj** | **ES CI Lower Adj** | **ES CI Upper Adj** | **p Adj** | **FDR p Unadj** | **FDR p Adj** | **FDR p (APOEe4+edu adj)** |
| --- | --- | --- | --- | --- | --- | --- | --- | --- | --- | --- | --- | --- | --- |
| volume | wmhVOLnormlog | CU_AB- vs CU_AB+ | 0.12 | 0.04 | 0.21 | 5.05e-3 | 0.06 | -0.01 | 0.14 | 0.11 | 7.58e-3 | 0.15 | 0.21 |
| volume | wmhVOLnormlog | CU_AB- vs MCI_AB+ | 0.22 | 0.14 | 0.30 | 2.43e-7 | 0.14 | 0.07 | 0.21 | 1.73e-4 | 7.28e-7 | 5.20e-4 | 0.00 |
| volume | wmhVOLnormlog | CU_AB- vs AD_AB+ | 0.27 | 0.19 | 0.36 | 7.76e-10 | 0.17 | 0.09 | 0.25 | 2.95e-5 | 4.66e-9 | 1.77e-4 | 0.00 |
| volume | wmhVOLnormlog | CU_AB+ vs MCI_AB+ | 0.10 | -0.01 | 0.22 | 0.08 | 0.08 | -0.02 | 0.18 | 0.13 | 0.08 | 0.15 | 0.14 |
| volume | wmhVOLnormlog | CU_AB+ vs AD_AB+ | 0.21 | 0.08 | 0.34 | 1.60e-3 | 0.14 | 0.02 | 0.26 | 0.03 | 3.20e-3 | 0.05 | 0.10 |
| volume | wmhVOLnormlog | MCI_AB+ vs AD_AB+ | 0.14 | 9.75e-3 | 0.27 | 0.04 | 0.09 | -0.03 | 0.20 | 0.15 | 0.04 | 0.15 | 0.14 |
