# Supplementary figures and images for: Spatial coupling of enlarged perivascular spaces and white matter lesions across the Alzheimer's disease continuum
Source: Front Neurosci. 2026 Apr 1;20:1772024. doi: 10.3389/fnins.2026.1772024 (PMC13079328; doi:10.3389/fnins.2026.1772024)

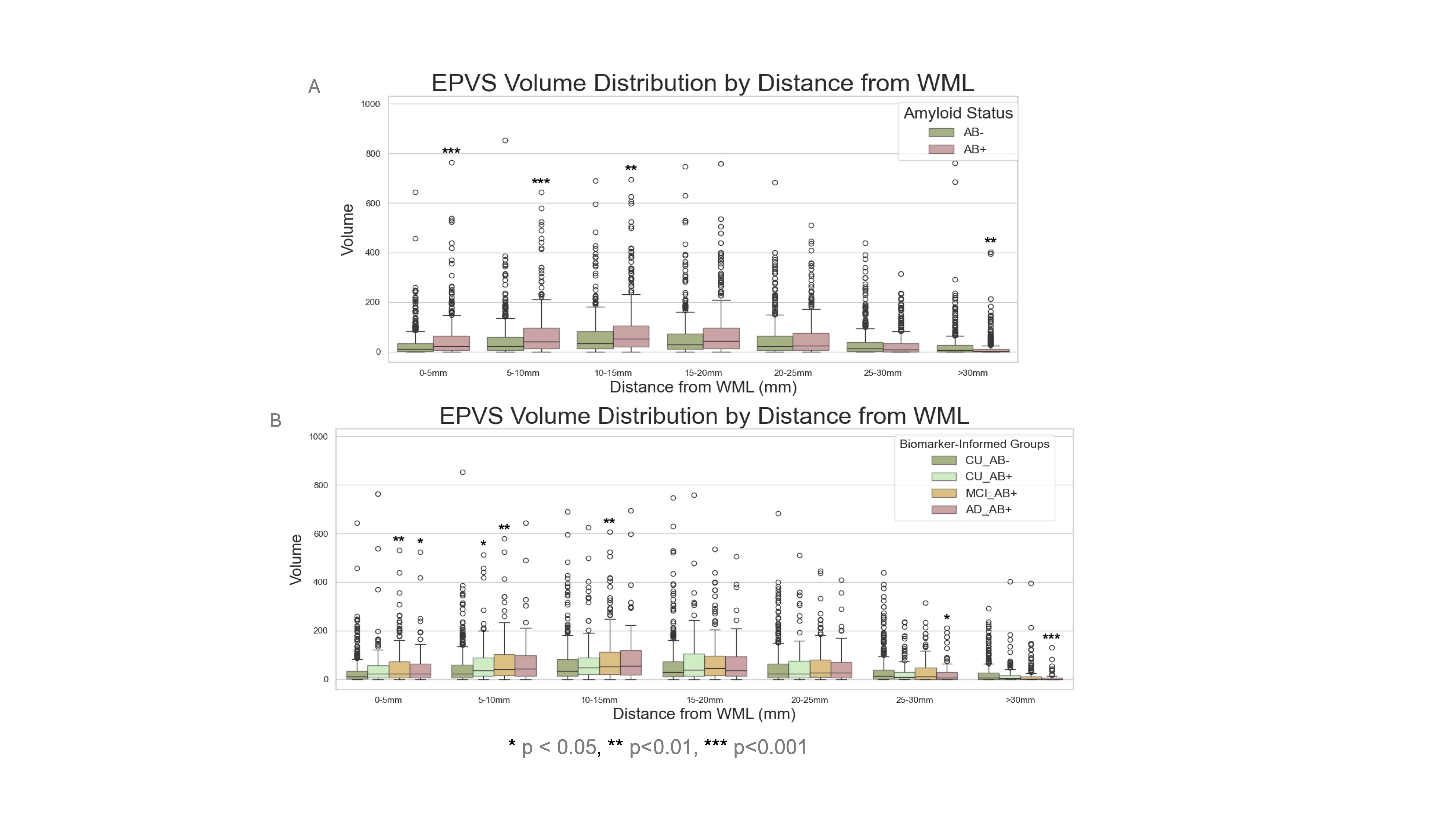

Supplement: Supplementary file 2 [file Image_1.jpeg]

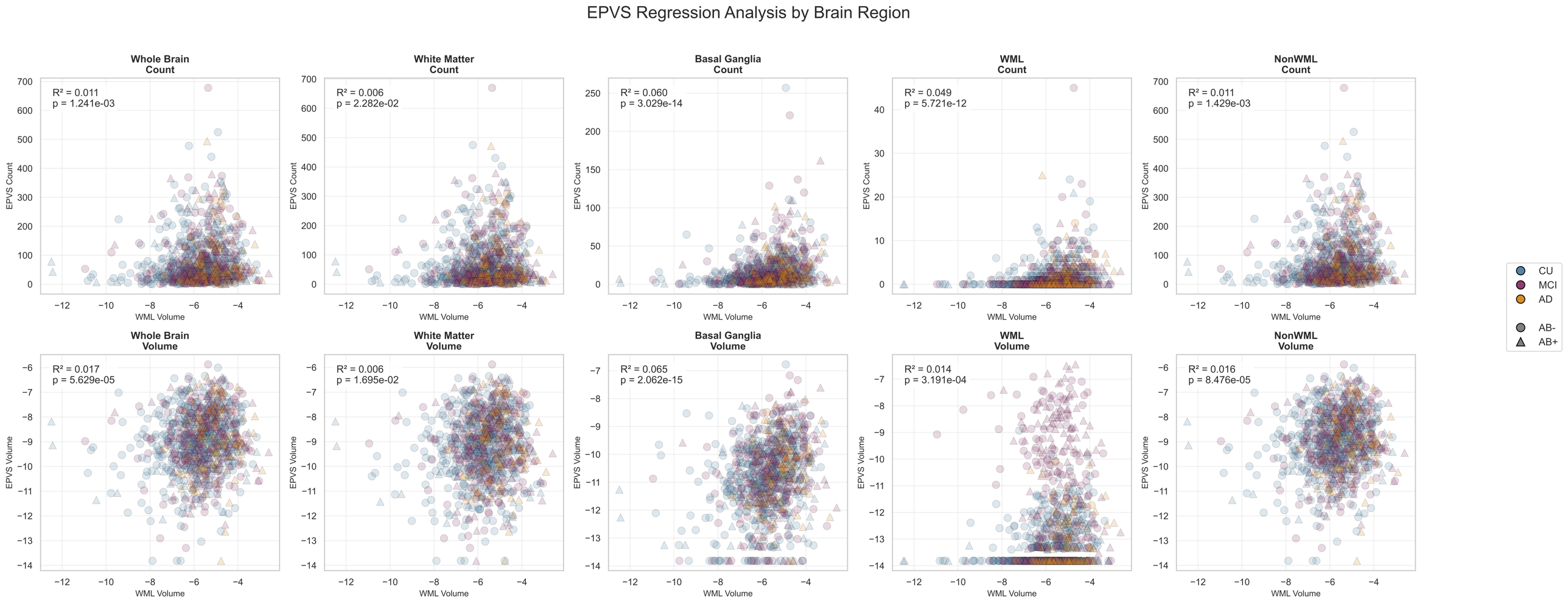

Supplement: Supplementary file 3 [file Image_2.jpeg]
